# Early ctDNA and Survival in Metastatic Colorectal Cancer Treated With Immune Checkpoint Inhibitors: A Secondary Analysis of the SAMCO-PRODIGE 54 Randomized Clinical Trial

**DOI:** 10.1001/jamaoncol.2025.1646

**Published:** 2025-06-18

**Authors:** Julien Taïeb, Francesco Giulio Sullo, Aurélie Lecanu, Camille Bourreau, Emilie Barbier, Annalice Gandini, Jérémie Bez, Claire Mulot, Frederic Di Fiore, Farid Elhajbi, Christophe Borg, Olivier Bouché, Thomas Aparicio, Aziz Zaanan, Thierry André, David Tougeron, Valerie Taly, Pierre Laurent-Puig

**Affiliations:** 1 Service de gastroenterologie et d’oncologie digestive, Paris CARPEM (Cancer research for personalized medicine) institute, Hopital Européen Georges Pompidou, Assistance Publique Hopitaux de Paris (AP-HP), Université Paris Cité, Paris, France; 2 Centre de Recherche des Cordeliers, Institut National de la Santé et de la Recherche Médicale, Université Paris Cité, Sorbonne Université, Equipe labellisée Ligue Nationale Contre le Cancer, Paris, France; 3 Department of Medical Oncology, Istituto di Ricovero e Cura a Carattere Scientifico, Istituto Romagnolo per lo Studio dei Tumori Dino Amadori, Meldola, Italy.; 4 Épidémiologie et Prévention Intégrées des Cancers de l’Appareil Digestif (Integrated Epidemiology and Prevention of Digestive Tract Cancers), Université de Bourgogne et Franche Comté, Dijon, France; 5 Service de gastroenterology, Centre Hospitalier Universitaire, hôpitaux de Rouen-Charles Nicolle, Rouen, France; 6 Medical Oncology Department, Oscar Lambret Center, Lille, France; 7 Department of Medical Oncology, University Hospital of Besançon, Besançon, France; 8 Department of Digestive Oncology, Centre Hospitalier Universitaire, Reims, Université Reims Champagne-Ardenne, Reims, France; 9 Department of Gastroenterology and Digestive Oncology, Saint Louis Hospital, Site de Recherche Intégrée sur le Cancer In Situ, Université Paris Cité, Paris, France; 10 Sorbonne Université, Hôpital Saint Antoine, AP-HP, and Unité Mixte de Recherche Scientifique 938, and Site de Recherche Intégrée sur le Cancer, Centre universitaire de Recherche actuelle molule sur le cancer, Paris, France; 11 Department of Gastroenterology and Hepatology, Poitiers University Hospital, Poitiers, France; 12 METHYS Dx, Paris, France; 13 European Liquid Biopsy Society, Hamburg, Germany; 14 Institut du Cancer Paris CARPEM, AP-HP, Université Paris Cité, Department of Biology, Hôpital Européen Georges Pompidou, Paris, France

## Abstract

**Importance:**

Immune checkpoint inhibitors (ICIs) have dramatically transformed the therapeutic landscape of deficient mismatch repair/microsatellite unstable−high (dMMR/MSI-H) metastatic colorectal cancer (mCRC); however, ICI use is challenged by primary resistance and timing of discontinuation. Whether circulating tumor DNA (ctDNA) may be predictive of progression-free survival (PFS) and overall survival (OS) in this treatment context remains unknown.

**Objective:**

To assess the prognostic and predictive role of ctDNA, detected by tumor-specific methylation markers, in patients with dMMR/MSI-H mCRC treated with ICIs.

**Design, Setting, and Participants:**

This prespecified secondary analysis of the SAMCO-PRODIGE 54 randomized clinical trial evaluated ctDNA in patients with dMMR/MSI-H mCRC treated with avelumab or standard chemotherapy, with or without a targeted agent in the second-line setting, to assess its prognostic role. Plasma samples were obtained prospectively for ctDNA analysis, and digital droplet polymerase chain reaction amplification of bisulfite-converted cell-free DNA (cfDNA) for *WIF1* and *NPY* genes was used to quantify ctDNA levels. These samples were collected from April 2018 to April 2021 at 49 sites in France at baseline (V1) and 1-month posttreatment initiation (V2) during. Data analyses were performed from October 1 to November 1, 2024.

**Intervention:**

Avelumab or standard chemotherapy with or without targeted agents.

**Main Outcomes and Measures:**

PFS and OS according to baseline ctDNA positivity or concentration, and early ctDNA variation (ΔctDNA = [V1−V2] ÷ V1).

**Results:**

The predictive analysis included 99 patients (mean [SD] age, 66 [13] years; 51 female [51.5%]) with plasma samples available for ctDNA assessment at V1, of which 74 had samples available also at V2 for Change in ctDNA assessment. In the 99 patients with available V1 plasma samples, baseline ctDNA positivity or concentration were not associated with clinical outcomes. Change in ctDNA (cutoff at median value) was significantly associated with both PFS (hazard ratio [HR], 2.98; 95% CI, 1.77-5.01; *P* < .001) and OS (HR, 3.61; 95% CI, 1.81-7.17; *P* < .001). This association was evident in patients treated with avelumab (PFS HR, 4.22; 95% CI, 1.77-10.1; *P* = .001; OS HR, 17.40; 95% CI, 3.82-79.70; *P* < .001) than in those receiving chemotherapy (PFS HR, 2.09; 95% CI, 1.03-4.21; *P* = .04; OS HR, 1.51; 95% CI, 0.61-3.72; *P* = .38). Avelumab (vs chemotherapy) improved PFS in favorable ctDNA responders (HR, 0.33; 95% CI, 0.14-0.77; log-rank *P* = .008) but not in poor responders (HR, 1.32; 95% CI, 0.67-2.62; log-rank *P* = .42) Combined ctDNA response and RECIST, version 1.1, assessment accurately predicted long-term OS. In the multivariable analysis, lack of ctDNA response was associated with an increased risk of disease progression and death in the avelumab group (HR, 7.27; 95% CI, 2.23-23.7; *P* = .001) but not in the chemotherapy group (HR, 1.61; 95% CI, 0.66-3.93; *P* = .30).

**Conclusions:**

The findings of this secondary analysis of an RCT found that change in ctDNA at 1-month posttreatment can predict long-term outcomes in patients with dMMR/MSI-H mCRC treated with ICIs.

**Trial Registration:**

ClinicalTrials.gov Identifier: NCT03186326

## Key Points

**Question:**

Can early circulating tumor DNA (ctDNA) variation predict long-term outcomes in patients with deficient mismatch repair/microsatellite instability-high (dMMR/MSI-H) metastatic colorectal cancer treated with immune checkpoint inhibitors (ICIs)?

**Findings:**

This prespecified secondary analysis of a randomized clinical trial assessed ctDNA in 99 patients with dMMR/MSI-H metastatic colorectal cancer who were treated with avelumab or standard chemotherapy and found that early ctDNA variation predicted progression-free and overall survival.

**Meaning:**

These findings indicate that early ctDNA monitoring may assist in determining ICI resistance and long-term survival in patients with dMMR/MSI-H metastatic colorectal cancer to support more tailored approaches to treatment.

## Teaser

This prespecified secondary analysis of a randomized clinical trial assesses the role of circulating tumor DNA in predicting progression-free and overall survival in patients with deficient mismatch repair/microsatellite instability−high metastatic colorectal cancer treated with immune checkpoint inhibitors.

## Introduction

Immune checkpoint inhibitors (ICIs) have dramatically transformed the therapeutic landscape of deficient mismatch repair/microsatellite unstable−high (dMMR/MSI-H) metastatic colorectal cancer (mCRC) and have led to improvements in response rates and progression-free survival (PFS).^1,2,3,4^ However, ICI use is still facing 2 substantial challenges. The first is primary resistance—that is, progressive disease (PD) at first disease assessment, usually 2 months—observed in 15% to 30% of patients with dMMR/MSI-H.^4,5^ The second challenge is determining when to discontinue treatment in patients with disease control, given that ICI is associated with long-term toxic effects and sizable health care costs. Furthermore, retrospective studies^6,7^ suggest that early treatment discontinuation can be an option in only selected cases.

In addressing these challenges, liquid biopsy may be a valuable tool, allowing treatment response assessment through circulating tumor DNA (ctDNA) monitoring.^8,9,10^ Analysis of hypermethylated genes by digital droplet polymerase chain reaction offers a cost-effective and tumor-agnostic approach, eliminating the need to identify specific pathogenic variants in tumor tissue.^11^ In particular, the detection of hypermethylated WNT inhibitor factor 1 and neuropeptide Y genes allows ctDNA quantification and has demonstrated association with long-term outcomes, both in the localized and metastatic settings.^12,13,14^

Given these findings, we decided to explore the predictive value of ctDNA in patients with dMMR/MSI mCRC treated with ICI. To achieve this, we used biological samples and clinical data from the SAMCO-PRODIGE 54 randomized clinical trial (RCT),^15^ which is to our knowledge the only RCT to compare avelumab to standard chemotherapy with and without targeted agents in patients with dMMR/MSI mCRC in the second-line setting. Results of the SAMCO-PRODIGE 54 demonstrated a significant advantage in PFS among patients treated with avelumab. Moreover, SAMCO-PRODIGE 54 was setup from the onset as a translational program, allowing discovery of predictive biomarkers of ICI efficacy or resistance to ICIs. Therefore, in this ancillary analysis, we sought to assess the prognostic and predictive role of ctDNA, detected by tumor-specific methylation markers, in the PFS and overall survival (OS) of patients with dMMR/MSI-H mCRC treated with ICIs.

## Methods

This prespecified secondary analysis used data from the SAMCO-PRODIGE 54,^15^ an RCT conducted in accordance with the principles of the Declaration of Helsinki and for which all participants provided written informed consent. The trial protocol, including its translational research program, received ethics-committee approval from the *Comité de Protection des Personnes Sud Méditerranée III* (Nîmes, France), and is available in Supplement 1. This study followed the Reporting Recommendations for Tumour Marker Prognostic Studies (REMARK) guidelines.

### Study Population

**Figure 1.**
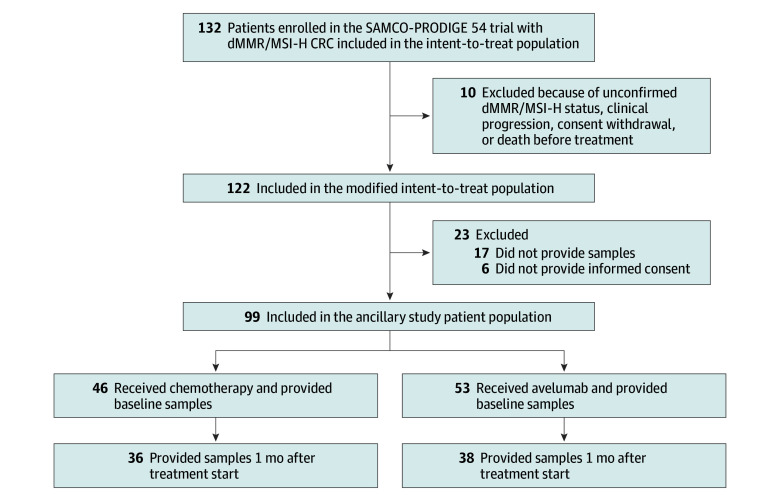
Study Flow Diagram of Included Patients

A total of 132 patients with mCRC and confirmed dMMR and MSI-H status were enrolled in SAMCO-PRODIGE 54 RCT^15^ and randomized in a 1:1 ratio to receive either avelumab (n = 65) every 15 days or standard chemotherapy with or without targeted therapy (n = 67). Our secondary analysis included patients who had previously provided informed consent for translational research and had supplied plasma samples for the assessment of ctDNA at baseline before starting treatment (V1) and before the third cycle of treatment (V2; 1 month after treatment start). Demographic information was collected from the case report forms of the clinical trial. A total of 173 plasma samples were available from the SAMCO-PRODIGE 54 RCT^15^; however, of 116 patients with a signed informed consent for the ancillary study, 17 did not provide a baseline (V1) sample, although 1 provided a V2 sample. Of the 99 patients for whom a V1 sample had undergone ctDNA testing, only 74 patients had a V2 sample for assessment of change in ctDNA. The study flowchart is shown in Figure 1.

### Procedures

After plasma collection in cell-free DNA tubes (Roche), centrifugated plasma samples were stored at −80 °C until DNA extraction. Cell-free DNA extraction and analysis was performed as described previously (eMethods in Supplement 2).^11,12,14^ All digital droplet polymerase chain reaction assays were performed according to current dMIQE guidelines.^16^

### Statistical Analysis

Overall response rate was defined as the proportion of patients achieving complete response (CR) or partial response (PR). Disease control rate was defined as the absence of PD (CR, PR, and stable disease) at 8 weeks; best response was defined as the best radiologic response observed at any time during treatment (PD, stable disease, PR, or CR). Radiographic follow-up was performed every 8 weeks. PFS was defined as the time from randomization to first disease progression, as assessed by investigators according to Response Evaluation Criteria in Solid Tumors (RECIST, version 1.1)^17^ or death. OS was defined as the time elapsed from randomization until death from any cause. Cox proportional hazards models were used to estimate hazard ratios (HRs) with 95% CIs for PFS and OS. Kaplan-Meier survival curves were generated and compared using the log-rank test. Change in ctDNA was calculated as a normalized difference ([V1−V2] ÷V1) and analyzed as categorical variables, using the median cutoff for categorization.

Favorable ctDNA responders were defined as those who demonstrated a change in ctDNA reduction equal to the median or greater; poor ctDNA responders showed a reduction less than the median. Considering (1) the low numbers of patients enrolled in the translational program and sizable attrition; and (2) the recent data of ctDNA in the adjuvant setting showing that patients who are ctDNA positive (postoperative) and then negative (postadjuvant chemotherapy) had similar outcomes as patients who are ctDNA negative remaining negative,^18^ we decided to classify the study patients with negative ctDNA at V1 and V2 as favorable ctDNA responders.

Baseline characteristics and response were described and compared between patients with baseline ctDNA-positive and ctDNA-negative profiles using Wilcoxon test and χ^2^ test (or Fisher exact test, if appropriate). Associations of baseline parameters with PFS and OS were first assessed using univariable Cox proportional hazard models; then, parameters with a *P* value < .10 were entered in a Cox regression multivariable model.

All statistical analyses were performed using R, version 4.3.1 (the R Foundation for Statistical Computing) from October 1 to November 1, 2024. *P* values < .05 were considered statistically significant; tests were 2-tailed.

## Results

### Baseline Characteristics of Study Participants

The analysis included 99 patients (mean [SD] age, 66 [13] years; 51 females [51.5%] and 48 males [48.5%]) with V1 samples. ctDNA was detectable in 83 patients (83.8%) and undetectable in 16 (16.2%). Baseline characteristics are shown in Table 1. Patients with liver metastases and more than 2 metastatic sites were more common in the ctDNA-positive group. Overall, disease control rates were similar between patients who were ctDNA-positive (60 patients [77%]) and ctDNA-negative (12 [75%]). Similar results were found for overall response rate (3 patients [19%] and 27 patients [35%]).

**Table 1.** Baseline Characteristics per Circulating Tumor DNA (ctDNA) Status at Baseline in Patients With Deficient Mismatch Repair/Microsatellite Instability-High Metastatic Colorectal Cancer

Characteristic	All patients (N = 99), No. (%)			Group, No. (%)					
				Avelumab			Chemotherapy		
	ctDNA negative	ctDNA positive	*P* value^a^	ctDNA negative	ctDNA positive	*P* value^b^	ctDNA negative	ctDNA positive	*P* value^b^
Patients, No.	16	83	NA	11	42	NA	5	41	NA
Sex									
Female	5 (31)	46 (55)	.08	4 (36)	26 (62)	.20	1 (20)	20 (49)	.40
Male	11 (69)	37 (45)		7 (64)	16 (38)		4 (80)	21 (51)	
Age, median (IQR), y	71 (56-75)	66 (59-77)	.80	71 (53-76)	68 (59-77)	.70	71 (58-74)	65 (59-76)	.90
WHO score									
0	8 (53)	36 (49)	.70	5 (50)	18 (49)	>.99	3 (60)	18 (49)	>.99
1-2	7 (47)	38 (51)		5 (50)	19 (51)		2 (40)	19 (51)	
Unknown No.	1	9		1	5		0	4	
Treatment									
Chemotherapy	5 (31)	41 (49)	.20	0	0	>.99	5 (100)	41 (100)	>.99
Avelumab	11 (69)	42 (51)		11 (100)	42 (100)		0	0	
Primary tumor resection	13 (81)	71 (86)	.70	9 (82)	35 (83)	>.99	4 (80)	36 (88)	.50
Metastatic sites, No.									
≤2	7 (50)	24 (31)	.20	5 (50)	12 (30)	.30	2 (50)	12 (32)	.60
>2	7 (50)	53 (69)		5 (50)	28 (70)		2 (50)	25 (68)	
Unknown	2	6		1	2		1	4	
Liver metastases									
Yes	2 (13)	40 (48)	.008	1 (9)	18 (43)	.07	1 (20)	22 (54)	.30
No	14 (88)	43 (52)		10 (91)	24 (57)		4 (80)	19 (46)	
*BRAF* V600E variant	7 (44)	37 (45)	>.99	4 (36)	19 (45)	.70	3 (60)	18 (44)	.60
Best response									
Complete response	1 (6)	11 (14)	.70	1 (9)	6 (15)	.90	0	5 (14)	.60
Partial response	2 (13)	16 (21)		1 (9)	7 (17)		1 (20)	9 (24)	
Stable disease	9 (56)	33 (42)		5 (45)	18 (44)		4 (80)	15 (41)	
Progressive disease	4 (25)	18 (23)		4 (36)	10 (24)		0	8 (22)	
Disease control rate	12 (75)	60 (77)		7 (63)	31 (76)		5 (100)	29 (79)	
Overall response rate	3 (19)	27 (35)		2 (18)	13 (32)		1 (20)	14 (38)	
Unknown No.	0	5		0	1		0	4	

Abbreviations: NA, not applicable; WHO, World Health Organization.

^a^ Pearson χ^2^ test; Wilcoxon rank sum test.

^b^ Fisher exact test; Wilcoxon rank sum test.

### Survival Outcomes

A total of 34 PFS events were observed in the chemotherapy group, compared to 29 in the avelumab group. Regarding OS, 19 events occurred in the chemotherapy group and 21 in the avelumab group.

First, we analyzed survival outcomes in relation to ctDNA positivity at baseline (V1). Although numerically higher survival was observed in patients who were ctDNA-negative, there were no statistically significant differences at baseline between those who were ctDNA-positive vs ctDNA-negative regarding regarding PFS (4.5 vs 8.2 months; HR, 1.32; 95% CI, 0.73-2.38; *P* = .36) or OS (20.0 months vs not reached; HR, 1.79; 95% CI, 0.81-3.94; *P* = .14) (eFigure 1 in Supplement 2). Then, we analyzed the association between PFS and baseline ctDNA concentration (median concentration cutoff = 1.7 ng/mL). No significant differences were noticed between high and low levels of ctDNA for PFS (5.0 vs 4.9 months; HR, 1.02; 95% CI, 0.66-1.55; *P* = .94). Better but not significant OS (33.0 vs 13.0 months; HR, 1.56; 95% CI, 0.93-2.62; *P* = .09) was observed in patients with low ctDNA concentration (eFigure 2 in Supplement 2).

The median value of change in ctDNA (–86%) was used as the cutoff to classify patients into favorable ctDNA responders and poor ctDNA responders. As recommended by the REMARK guidelines, the relation of the marker to standard prognostic variables for PFS is summarized in eTable 1 in Supplement 2.

**Figure 2.**
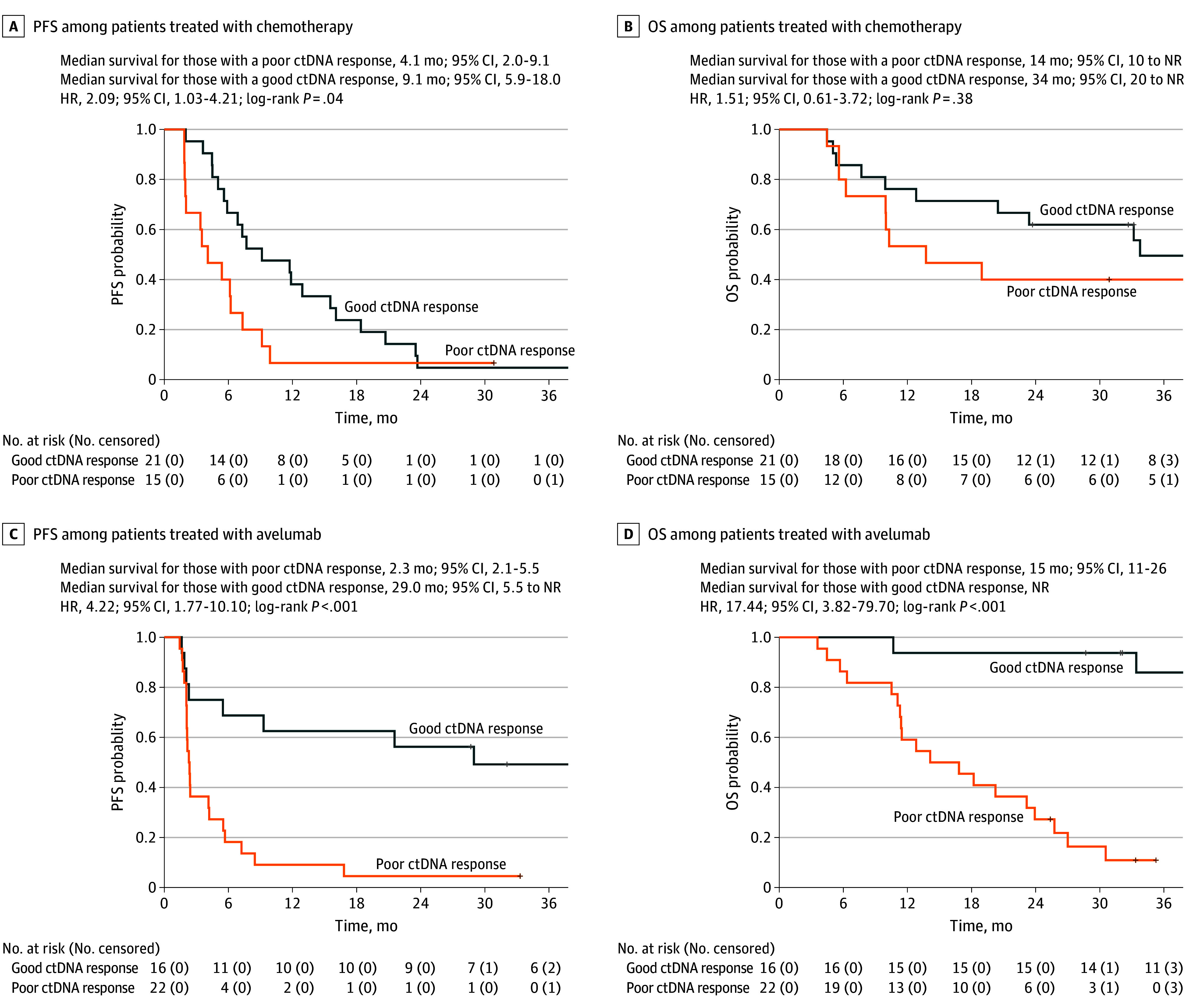
Progression-Free Survival (PFS) and Overall Survival (OS) per Circulating Tumor DNA (ctDNA) Response in Patients Treated With Chemotherapy vs Abelumab In patients with deficient mismatch repair/microsatellite instability-high metastatic colorectal cancer, favorable responders were those with a ctDNA reduction greater than 86%, and poor responders, reduction of 86% or less. HR indicates hazard ratio; NR, not reached.

A significant difference in median PFS was identified between favorable and poor ctDNA responders in the overall population, with median PFS of 12.0 and 2.4 months, respectively (HR, 2.98; 95% CI, 1.77-5.01; *P* < .001; eFigure 3 in Supplement 2). Similarly, in the chemotherapy group, median PFS was 9.1 and 4.1 months (HR, 2.09, 95% CI, 1.03-4.21; *P* = .04; Figure 2A) and in the avelumab group, median PFS was 29.0 and 2.3 months (HR, 4.22; 95% CI, 1.77-10.1; *P* = .001; Figure 2C).

OS analyses also revealed significant differences between favorable and poor ctDNA responders in the overall population, with median OS not reached and 14.0 months, respectively (HR, 3.61; 95% CI, 1.81-7.17; *P* < .001; eFigure 3 in Supplement 2). In the chemotherapy group, median OS was 34.0 and 14.0 months (HR, 1.51; 95% CI, 0.61-3.72; *P* = .40) with no statistical difference between the 2 groups (Figure 2B). In the avelumab group, median OS was not reached for favorable ctDNA responders and 15.0 (95% CI, 11.0-26.0) months for poor ctDNA responders (HR, 17.40; 95% CI, 3.82-79.70; *P* < .001; Figure 2D).

**Figure 3.**
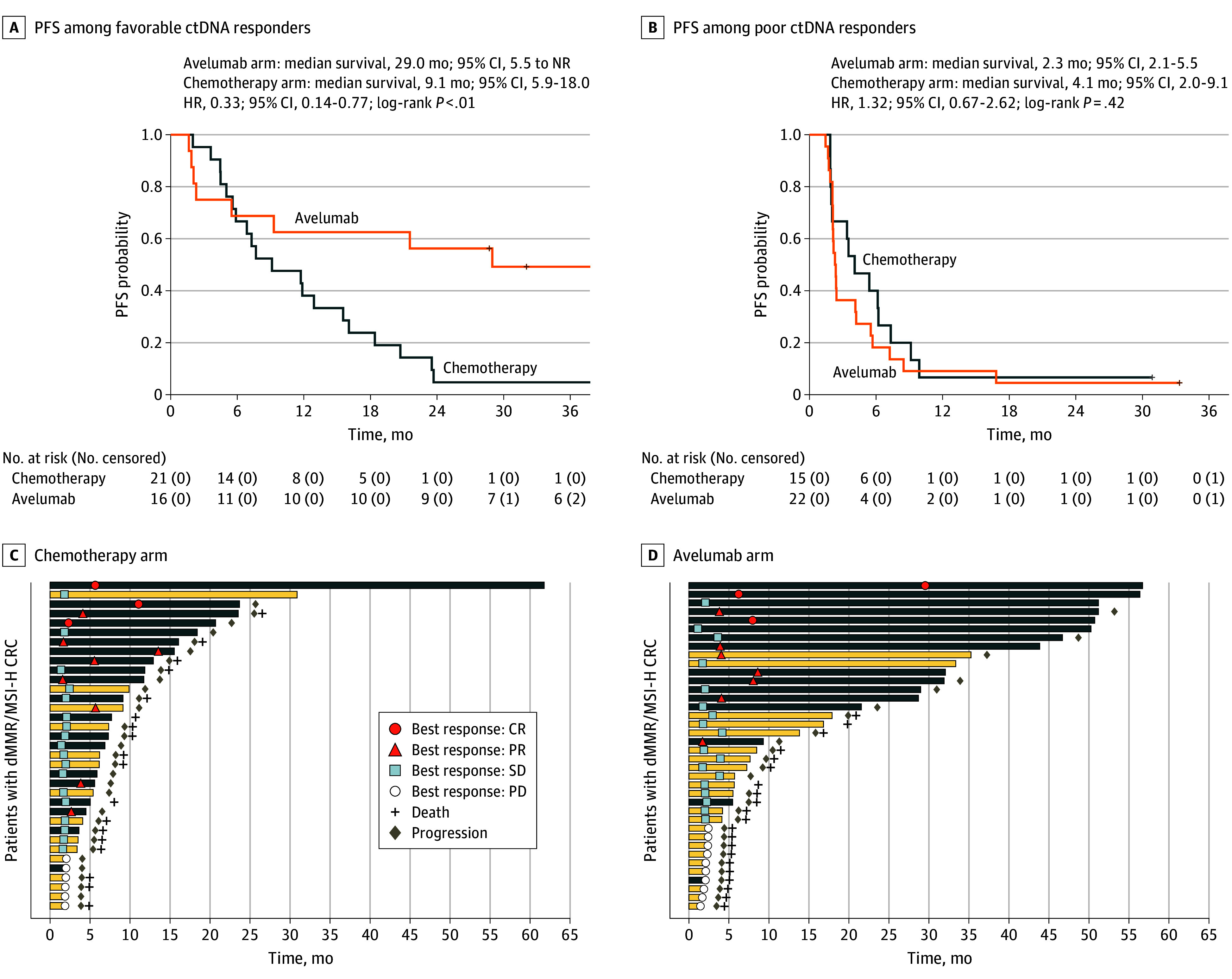
Progression-Free Survival (PFS) in Patients With Deficient Mismatch Repair/Microsatellite Instability-High Metastatic Colorectal Cancer Treated With Chemotherapy vs Avelumab A and B, Favorable responders had a ctDNA reduction greater than 86%, and poor responders, reduction of 86% or less. C and D, Early change in ctDNA variations between baseline and on-treatment assessable ctDNA levels, RECIST response, and PFS in patients treated with chemotherapy (n = 35) vs avelumab (n = 38). Patients with both V1 and V2 measures are shown in the swimmer plots according to PFS, with total length of bar indicating PFS duration. CR indicates complete response; ctDNA, circulating tumor DNA; HR, hazard ratio; NR, not reached; PD, progressive disease; PR, partial response; RECIST, Response Evaluation Criteria in Solid Tumors, version 1.1; SD, stable disease.

We also compared avelumab to chemotherapy separately in favorable vs poor ctDNA responders (Figure 3A and B). The results show an improvement in PFS in patients treated with avelumab compared with those treated with chemotherapy in favorable ctDNA responders (HR, 0.33; 95% CI, 0.14-0.77; *P* = .008) but not in poor ctDNA responders (HR, 1.32; 95% CI, 0.67-2.62; *P* = .42).

To enhance the predictive power of change in ctDNA determination, we evaluated it in combination with radiological and clinical response. As shown in the 2 swimmer plots (Figure 3C and D), at total of 6 patients reached CR—all of them were favorable ctDNA responders. In addition, 13 of 15 patients with PR were favorable ctDNA responders. Stable disease was recorded in 36 patients among whom 15 were favorable ctDNA responders and 21 were poor ctDNA responders. Lastly 14 of 16 patients with PD were poor ctDNA responders. Among the patients who were progression-free at 20 months in the avelumab group, 13 (87%) were favorable responders. The distribution of baseline ctDNA and change in ctDNA according to RECIST^17^ best response in both treatment groups is displayed in eFigure 4 in Supplement 2.

eFigure 5 in Supplement 2 shows that OS varied significantly among patient groups based per RECIST^17^ and ctDNA responses. Patients with disease control and favorable ctDNA response exhibited the longest OS (median survival, not reached), whereas patients with PD and poor ctDNA response exhibit the shortest OS (11.0 months).

### Univariable and Multivariable Analyses

To evaluate the risk of progression or death, we first identified parameters with a *P* value < .10 in the univariable analysis (eTable 2 in Supplement 2) for inclusion in the multivariable Cox regression models (Table 2). We observed a significant interaction term between treatment group and change in ctDNA (95% CI, 1.14-9.83; *P* = .03), allowing the analysis of each treatment group independently. We integrated this interaction term in our final multivariable model including all patients.

**Table 2.** Multivariable Analyses for Progression-Free Survival per Clinical and Molecular Variables

Variable	Both groups		Chemotherapy		Avelumab	
	HR (95% CI)	*P* value	HR (95% CI)	*P* value	HR (95% CI)	*P* value
Liver metastases						
No	1 [Reference]	.60	1 [Reference]	.10	1 [Reference]	.30
Yes	1.18 (0.65-2.17)		1.96 (0.88-4.35)		0.60 (0.23-1.56)	
Metastatic site, No.						
≤2	1 [Reference]	.70	1 [Reference]	.70	1 [Reference]	.60
>2	1.15 (0.61-2.18)		1.17 (0.49-2.78)		1.32 (0.51-3.42)	
Log transform CEA	1.62 (1.13-2.32)	.009	2.10 (1.31-3.36)	.002	1.44 (0.82-2.54)	.20
Neutrophil to lymphocyte ratio	0.97 (0.85-1.10)	.60	1.08 (0.92-1.27)	.30	0.87 (0.72-1.06)	.20
Change in ctDNA^a^						
Favorable ctDNA responder	1 [Reference]	.13	1 [Reference]	.30	1 [Reference]	.001
Poor ctDNA responder	1.83 (0.83-4.05)		1.61 (0.66-3.93)		7.27 (2.23-23.7)	
Treatment group						
Chemotherapy	1 [Reference]	.02	1 [Reference]	NA	1 [Reference]	NA
Avelumab	0.35 (0.14-0.86)		1 [Reference]		1 [Reference]	
Change in ctDNA treatment group						
Poor ctDNA responder treated with avelumab	4.66 (1.41-15.30)	.01	1 [Reference]	NA	1 [Reference]	NA

Abbreviations: CEA, carcinoembryonic antigen; HR, hazard ratio; NA, not applicable.

^a^ The 2 groups favorable and poor ctDNA responders were separated based on a median threshold of 86% ctDNA decrease.

For the whole study population, carcinoembryonic antigen (CEA) levels and treatment with avelumab were confirmed as independent predictors of PFS in the overall population. In patients treated with chemotherapy, CEA level was independently associated with PFS but not with change in ctDNA. At the opposite, change in ctDNA was confirmed to be independently associated with PFS in the patients treated with avelumab (HR, 7.27; 95% CI, 2.23-23.7; *P* = .001), but not CEA level. Taken together, these findings underscore the role of change in ctDNA variation as a dynamic prognostic biomarker that complements static baseline indicators, particularly in patients receiving avelumab therapy.

## Discussion

Biomarker-directed use of ICI is an important frontier in precision medicine, and there is an ongoing need to better understand the potential role of ctDNA in advanced dMMR/MSI-H mCRC, including its prognostic value and its role for monitoring patients and predict long-term outcomes. This prespecified secondary analysis of a phase 2 randomized clinical trial addressed these points in a carefully designed exploratory analysis to evaluate patients selected from the avelumab monotherapy and chemotherapy groups of the SAMCO-PRODIGE 54 study.^15^

The findings of this study showed that ctDNA was detectable in 83.8% of the patients with dMMR/MSI-H mCRC. Liver metastases were notably associated with ctDNA positivity, consistent with prior findings.^19,20^ We found that for both treatment regimens, a moderate prognostic value of baseline ctDNA with baseline ctDNA positivity was associated with shorter median PFS (4.5 vs 8.2 months) and OS (20.0 months vs not reached), without reaching significance. When considering ctDNA concentration at baseline, PFS was very similar between patients with high and low ctDNA concentrations (4.9 vs 5.0 months), but OS was shorter in patients with high baseline ctDNA concentrations (13.0 vs 33.0 months), without reaching significance, possibly due to the limited number of patients included in this analysis. High concentrations may reflect a higher disease burden and a more aggressive disease profile, potentially limiting access to subsequent treatments at disease progression, and thus, affecting OS but not PFS.

Conversely, early change in ctDNA variations significantly associated with PFS and OS, with differences between chemotherapy and avelumab. The distribution of change in ctDNA was different for groups defined by treatment group and RECIST^17^ response. Among 21 patients with CR or PR (per RECIST), 19 (90.5%) were favorable ctDNA responders. Conversely, among 16 patients with PD (per RECIST), 14 (87.5%) were poor ctDNA responders, which demonstrates that those who have primary resistance seemed to be mostly poor ctDNA responders. Altogether, despite a clear association between change in ctDNA and tumor assessment per RESIST, several patients with poor ctDNA response experienced long-lasting stable disease (Figure 3C and D), making ctDNA informative, but not fully able to drive clinical decision of early treatment discontinuation. Similarly, change in ctDNA did not identify patients who had primary resistance with the sensitivity and specificity levels needed in routine practice. Lastly, the combination of change in ctDNA and RECIST seemed superior to RECIST alone for predicting OS (eFigure 5 in Supplement 2), showing that ctDNA could provide complementary information to RECIST.

In addition to RECIST tumor assessment, we found an association between change in ctDNA and survival, both OS and PFS. This association was greater in the avelumab group, likely reflecting its therapeutic efficacy that offers durable, long-term diseases control compared to short-lived chemotherapy effects. Lastly, chemotherapy and ICIs have different modes of action and effects on the dynamics of tumor growth and tumor-cell elimination, which may also explain the differences observed.

Until now, few putative biomarkers have shown predictive value in patients with dMMR/MSI-H cancer treated with ICI. Tumor mutational burden, inactivation of antigen presentation, programmed death-ligand 1 (PD-L1) expression, and β2-microglobulin have failed to identify patients with dMMR/MSI-H mCRC who are resistant to ICI.^4^ In patients with dMMR/MSI-H mCRC treated with ICI, associations with survival outcomes (PFS and OS) have been reported for Eastern Cooperative Oncology Group Performance Status (ECOG-PS), number of metastatic sites, neutrophil to lymphocyte ratio, and liver metastases.^5,21,22,23^ Our multivariable analysis included all these factors (except for ECOG-PS because only 2 patients had an ECOG-PS score of 2 in this series), as well as additional factors with a *P* < .10 in univariable analysis. We found that only CEA levels and change in ctDNA were associated with shorter PFS in patients treated with avelumab in univariable analyses, and that only change in ctDNA remained significant in the final multivariable model, with a high HR of 7.27 (95% CI, 2.23-23.7; *P* = .001). Conversely, when evaluating patients treated with chemotherapy, only CEA level (HR, 2.10; 95% CI, 1.31-3.36; *P* = .002) was associated with PFS. Interestingly, this ability of change in ctDNA to predict long-term outcomes in patients treated with ICI, but not chemotherapy, has been recently reported in patients with metastatic urothelial cancers treated in the Keynote 361 study.^24^ Another work performed on 5 distinct cohorts of patients with solid tumors treated with pembrolizumab^25^ also suggested that ctDNA kinetics during treatment were strongly associated with PFS and OS.

In developing predictive markers, tissue-based analyses providing transcriptomic signatures have shown potential^26^ but are limited by spatial heterogeneity and invasive tissue sampling. Liquid biopsy provides real-time, comprehensive insights into tumor status, with fewer spatial limitations.

Monitoring of ctDNA dynamics in patients treated with ICI may pave the way to broader application of biomarker-directed ICI treatments. ctDNA can be noninvasively assessed serially during treatment and has recently improved in availability and use. The methylation-based assay used in this analysis is a tumor-agnostic approach that uses plasma-only ctDNA universal CRC-methylated markers.^11,14,19^ This approach shows a limit of detection of 0.2% and is relevant for the metastatic setting where ctDNA concentration is generally sufficiently elevated. Personalized tumor-informed tests detect very low ctDNA levels, which is ideal for early-stage disease and minimal residual disease detection. However, these assays may not be necessary in the metastatic setting, particularly in a well-shedding tumor type such as mCRC. In late-stage disease, limited tumor tissue from initial biopsy specimens often makes tumor-informed tests impractical for many patients.

Our findings suggest that change in ctDNA may theoretically be useful for early prediction of ICI efficacy. Therefore, the added value of change in ctDNA is having an earlier assessment and providing additional information on the long-term outcome of the disease compared with a classic RECIST assessment.^17^ change in ctDNA may also be useful for patients in which RECIST evaluation is not helpful, such as in patients with nonmeasurable disease, mixed response, or pseudo-progression; however, these patients were not present in our study population. Clinical validation studies conducted in the routine community setting should be encouraged to assess the usefulness of change in ctDNA in specific clinical contexts.

### Limitations

Our study has several limitations. Although we strategically aimed to select a representative set of patients for this work, our findings are limited by a moderate sample size. In particular, trends in subgroups created by segregating multiple biomarkers should be interpreted with caution, and larger prospective studies designed to compare change in ctDNA patterns in the context of ICI treatment are needed to confirm our results. A potential limitation is also that the study end points (PFS and OS) are defined as time from randomization to the event, but the patients are not at risk for an event until at least 4 weeks, for analyses with nonmissing change in ctDNA, which may have introduced a potential risk for immortal-time bias. Another limitation is the single ctDNA monitoring time point given that delayed clearance, which has already been reported in the literature,^25^ may need additional points for better outcome predictions. Other limitations are the use of median change as the cutoff value for our analyses and the use of an anti−PD-L1 molecule, although only programmed cell death 1 protein inhibitors are currently approved for patients with dMMR/MSI mCRC.

## Conclusions

This secondary analysis of the SAMCO-PRODIGE 54 RCT suggests that ctDNA-based surveillance offers a noninvasive strategy to predict clinical benefit and survival in patients with dMMR/MSI-H affected by mCRC. Further studies possibly involving multiple ctDNA time points together with a predefined threshold for ctDNA decrease are thus warranted. This may drive the design of future clinical trials to assess, for example, whether long-term survivors achieving a favorable ctDNA response at 1 month, or even earlier, could benefit from early treatment discontinuation, or whether those achieving a poor ctDNA response may benefit from an early treatment switch. Putative biomarkers could implement the selection of patients who could benefit from ICI treatment. Future studies should validate ctDNA kinetics to guide ICI treatment decisions in this population.

## Notes

Supplement 1. Trial Protocol and Statistical Analysis Plan

Supplement 2. eMethods. Cell free DNA extraction and analysis eFigure 1. PFS (A) and OS (B) according to ctDNA status at baseline (positive versus negative) eFigure 2. PFS (A) and OS (B) according to ctDNA concentration at baseline (above and below median; median= 1.7ng/mL) eFigure 3. PFS (A) and OS (B) according to ctDNA response eFigure 4. DNA concentrations in ng/mL at baseline (V1) and after one month of treatment (V2) according to treatment group and treatment response e Figure 5. Early ΔctDNA variations between V1 and V2, disease control according to RECIST and overall survival eTable 1. Relation of ctDNA response to standard prognostic variables for PFS in MSI mCRC patients treated with immunotherapy eTable 2. Univariable analysis of PFS based on key clinical and molecular variables

Supplement 3. Data Sharing Statement
