# Supplementary material for: Early ctDNA and Survival in Metastatic Colorectal Cancer Treated With Immune Checkpoint Inhibitors: A Secondary Analysis of the SAMCO-PRODIGE 54 Randomized Clinical Trial
Source: JAMA Oncol. 2025 Jun 18;11(8):874–82. doi: 10.1001/jamaoncol.2025.1646 (PMC12177728; doi:10.1001/jamaoncol.2025.1646)
Supplement: Supplement 1. — Trial Protocol and Statistical Analysis Plan [file jamaoncol-e251646-s001.pdf]

PRODIGE 54 (FFCD 1603) – SAMCO

MULTICENTER RANDOMIZED PHASE II STUDY COMPARING THE EFFECTIVENESS AND TOLERANCE OF AVELUMAB VERSUS STANDARD 2nd LINE TREATMENT CHEMOTHERAPY IN PATIENTS WITH COLORECTAL METASTATIC CANCER WITH MICROSATELLITE INSTABILITY (MSI)

Multicenter randomized phase 2

EudraCT No. 2016-004575-49

Intergroup study FFCD - UNICANCER GI - GERCOR

COORDINATOR (FFCD)
Prof. Julien TAIEB
HOPITAL EUROPEEN G. POMPIDOU
Department of Hepato-Gastroenterology
20 Rue Leblanc
75015 PARIS
Tel.: 01 56 09 50 42 - Fax: 01 56 09 35 29
Email: julien.taieb@egp.aphp.fr

CO-COORDINATOR (FFCD)
Prof. David TOUGERON
CHU LA MILETRIE
Service d'Hépato-gastro-entérologie
2 Rue de la Milétrie - BP 577
86021 POITIERS
Tel.: 05 49 44 37 51 - Fax: 05 49 44 38 35
Email:david.tougeron@chu-poitiers.fr

CO-COORDONNATOR (UNICANCER):
Dr Christelle de la Fouchardière
CENTRE LEON BERARD - Service de Médecine
28 Rue Laennec
69373 LYON
Courriel : christelle.delafouchardiere@lyon.unicancer.fr

CO-COORDONNATOR (GERCOR):
Pr Thierry ANDRE
HOPITAL SAINT ANTOINE
Service d'Oncologie Médicale
184 rue du Faubourg Saint Antoine
Paris 75012, France.
Courriel :Thierry.Andre@sat.aphp.fr

EDITORIAL COMMITTEE
Julien TAIEB, David TOUGERON, Emilie Barbier, Marie MOREAU, Jérémie BEZ Jean-Marc PHELIP, Astrid LIEVRE, Thierry LECOMTE

BIOLOGICAL COMMITTEE
Pierre LAURENT-PUIG, Jean-François EMILE

SPONSOR AND RANDOMIZATION-MANAGEMENT-ANALYSIS CENTER (RMAC):
Fédération Francophone de Cancérologie Digestive **(FFCD)**, Faculty of Medicine, 7 boulevard Jeanne d'Arc, BP 87900, 21079 Dijon Cedex

Administrative Director: **Cécile GIRAULT**
Tel.: 03 80 66 80 13 – Fax: 03 80 38 18 41
Email: cecile.girault@u-bourgogne.fr

Statistician: **Emilie BARBIER**
Tel: +33 (0)3 80 39 34 81 – Fax: +33 (0) 3 80 38 18 41
Email: emilie.barbier@u-bourgogne.fr

Project Leader: **Jérémie BEZ**
Tel.: 03 80 39 34 83 – Fax: 03 80 38 18 41
Email: jeremie.bez@u-bourgogne.fr

Confidential
This document is the property of the Fédération Francophone de Cancérologie Digestive and cannot be transmitted, duplicated, published or used - in full or in part - without the specific permission of the Fédération Francophone de Cancérologie Digestive.

CONTENTS

TRIAL OBJECTIVES **12**
Main objective 12
Secondary objectives 12
PATIENT SELECTION FOR REGISTRATION **12**
Inclusion criteria 12
Non-inclusion criteria 13
INCLUSION REPORT **14**
RANDOMIZATION **14**
TRIAL SCHEME **16**
TREATMENTS **16**
6.1 Arm A FOLFOX or FOLFIRI +/- targeted therapy (standard treatment) 16
Arm B - Avelumab (experimental arm) 17
DOSE ADJUSTMENT ACCORDING TO TOXICITIES **18**
FOLFOX OR FOLFIRI AND TARGETED THERAPIES 18
BEVACIZUMAB 22
PANITUMUMAB 23
CETUXIMAB 24
AFLIBERCEPT 26
AVELUMAB 28
Severe Hypersensitivity Reactions and Flu-Like Symptoms *29*
Immune-Related Adverse Events *30*
PREMEDICATION, CONCOMITANT TREATMENTS AND CONTRAINDICATED TREATMENTS ... 34
LOGISTICS OF THE BIOLOGICAL STUDY **35**
PATIENT MONITORING **36**
Before each administration of treatment 36
Evaluation every 8 weeks 36
After discontinuation of the treatment: 36
Within 30 days 36
After premature discontinuation for reason other than radiological progression* 37
After radiological progression 37
1ST LINE TREATMENTS AND SUBSEQUENT TREATMENTS **37**
MANAGEMENT OF SERIOUS ADVERSE EVENTS (SAE) **37**
STATISTICAL ANALYSIS **39**
Judgement criteria 39
Main efficacy criterion 39
Secondary criteria 40
Calculating the number of subjects required, statistical hypotheses 41
STUDY COMMITTEES **42**
Independent committee 42
Steering committee 42
Medical journal 42
Biological research committee 42

BASIC INFORMATION AND JUSTIFICATION FOR THE STUDY 42
ADMINISTRATIVE CONSIDERATIONS 43
RULES OF PUBLICATION 45
APPENDICES 45
APPENDIX 1: CLINCIAL INFORMED CONSENT 46
APPENDIX 2: BIOLOGICAL INFORMED CONSENT 47
APPENDIX 3: BIOLOGICAL STUDY 48
APPENDIX 4: WHO PERFORMANCE INDEX – CLEARANCE CALCULATION 50
APPENDIX 5: RECIST CRITERIA VERSION 1.1 AND IMMUNE RELATED RECIST 51
APPENDIX 6: EVALUATION OF TOXICITY (NCI CTC V4.0) 53
APPENDIX 7: SUMMARY PRODUCT CHARACTERISTICS OF THE STUDY (OXALIPLATIN, IRINOTECAN, 5FU, AF, TARGETED TREATMENTS) 54
APPENDIX 8: QUALITY OF LIFE QLQ-C30 55
APPENDIX 9: SERIOUS ADVERSE EVENT DECLARATION FORM 57
APPENDIX 10: RULES OF PUBLICATION OF PRODIGGE TRIALS 63
11: INSURANCE ATTESTATION 65
APPENDIX 12: FAVORABLE DECISION BY THE CPP 66
APPENDIX 13: ANSM AUTHORISATION 67

LIST OF ABBREVIATIONS

ADR	Adverse Drug Reaction
CEA	Carcinoembryonic antigen
ALAT	Alanine-aminotransferase (or SGPT: serum glutamic pyruvic transaminase)
ANSM	Agence nationale de sécurité du médicament et des produits de santé (French national agency of medecines and health products safety)
CRA	Clinical Research associate
ASAT	Aspartate-aminotransferase (or SGOT: serum glutamic oxalo-acetic transaminase)
BMI	Body mass index
CPP	Comité de Protection des Personnes (Protection of Persons Committee)
CI	Contraindication
CT	Chemotherapy
CTC	Common toxicity criteria
DPD	Dihydropyrimidideshydorgenase
AE	Adverse event
SAE	Serious adverse event
5FU	5-fluorouracil
FFCD	Fédération Francophone de Cancérologie Digestive (French-speaking federation of digestive oncology)
GGT	Gamma-glutamyl transpeptidase
Hb	Hemoglobin
HR	Hazard ratio
HBP	High blood pressure
IHC	Immunohistochemistry
INR	International Normalized Ratio
MRI	Magnetic resonance imaging
IRECIST	*Immune-related* RECIST
ITT	Intention to treat
IV	Intravenous
D	Day
KM	Kaplan Meier
LDH	Lactate dehydrogenase
UNL	Upper normal limit
mTNS	Modified Total Neuropathy Score
N	Normal
NCI-CTCAE	National Cancer Institute – Common Terminology Criteria for Adverse Events (Critères de toxicité courants pour les événements indésirables)
CBC	Complete blood count
WHO	World Health Organization
ALP	Alkaline phosphatases
PNN	Polynuclear neutrophil
Q1-Q3	Quartiles
RECIST	Response Evaluation Criteria In Solid Tumors
CR	Complete response:
PR	Partial response:
OR	Objective response
OS	Overall survival
PFS	Progression-free survival
SD	Stable disease
TAP	Thoracolumbar Abdomino-Pelvic
TDM	Tomodensitometry
PT	Prothrombin time (level)
UICC	Union for International Cancer Control

CONTACTS AT FFCD FOR THE STUDY

NAME	FUNCTION	TELEPHONE	FAX	EMAIL
**CONTACTS AT FFCD ADMINISTRATIVE**				
Cécile GIRAULT	Executive Director	03 80 39 33 87	03 80 38 18 41	cecile.girault@u-bourgogne.fr
**RANDOMISATION-MANAGEMENT-ANALYSIS CENTRE**				
Marie MOREAU	Clinical operations manager	03 80 39 34 04	03 80 38 18 41	marie.moreau@u-bourgogne.fr
Jérémie BEZ	Project manager	03 80 39 34 83	03 80 38 18 41	jeremie.bez@u-bourgogne.fr
Emilie BARBIER	Biostatistician	07.82.81.97.61	03 80 38 18 41	emilie.barbier@u-bourgogne.fr
Francis DIEDHIOU	Data Manager	03 80 39 34 05	03 80 38 18 41	Francis.Diedhiou@u-bourgogne.fr
Géraldine VAUDRIT	Assistant Project Leader	03 80 39 33 86	03 0 38 18 41	Geraldine.Vaudrit@u-bourgogne.fr
Caroline CHOINE-POURRET	CRA Coordinator	04 69 18 19 02	09 74 44 22 47	carolinechoine@u-bourgogne.fr
Camille FLECK	Pharmacovigilance leader	03 80 29 55 80		vigilance-ffcd@hotmail.com

PROTOCOL ACCEPTANCE FORM

PRODIGE STUDY 54 (FFCD 1603) – SAMCO

MULTICENTER RANDOMIZED PHASE II STUDY COMPARING THE EFFECTIVENESS AND TOLERANCE OF AVELUMAB VERSUS STANDARD 2nd LINE TREATMENT CHEMOTHERAPY IN PATIENTS WITH COLORECTAL METASTATIC CANCER WITH MICROSATELLITE INSTABILITY (MSI)

A prospective, multicenter, open-label, randomized, active-controlled, phase 2 study to compare the efficacy and safety of avelumab versus standard second line treatment, in patients with MSI+ metastatic colorectal cancer after first line treatment failure.

EudraCT No2016-004575-49- Version 5.0 31/07/2020

This version of the protocol was approved by:

The Sponsor: Mrs Cécile GIRAULT Date: 31.07.2020 Signature:

The Coordinator: Prof. Julien TAIEB Date: 31.07.2020 Signature:

I,the undersigned, Dr: ....

After reading the prerequisites for this research, the protocol and its appendices, I certify that I shall carry out this trial in respect of Good Clinical Practices and in agreement with the applicable provisions of the Public Health Code.

I specifically undertake to:
respect protocol and any modifications notified by the Sponsor
agree to supervise the research in the center and train my colleagues in the procedure, and to provide a nominative list of my colleagues
ask the town halls where births are registered, in the case of patients lost to follow-up, the condition of the patients at the time of analysis or when the Sponsor asks me
have each patient sign an informed consent form after explaining the information sheet given to patients and before carrying out any act linked to the research
declare serious adverse events or new occurrences within 24 hours of being informed of them, in accordance with the research protocol
respect inclusion and non-inclusion criteria and the study start and end dates
take part in the biological part of the study and send samples in accordance with recommendations
fill in all the items in the case report form, check the quality of data collection and ensure that the products are managed properly
preserve the data and documents related to the research for a period of 15 years after the end of the study
inform the Sponsor of any situation of conflict of interest that may affect my scientific independence as part of the research
inform the Sponsor immediately of any action, friendly or contentious, taken by someone taking part in the research or their beneficiaries, which is likely to infringe on the liability of the Sponsor
accept periodic visits by the Sponsor's representatives; provide them with all source documents and equipment relative to the research for the purposes of ensuring quality control of the data recorded in the case report form.
agree to a check in the form of an audit by the Sponsor or one of their representatives and/or inspection by the health authorities.
answer requests for corrections or details concerning the case report form, by phone or post
allow the necessary time for the FFCD CRA to sign the sheets, answer any questions and take any required action

Date: Signature:

STAMP of the CENTER:

Send the original to the FFCD RMAC – 7 boulevard Jeanne d'Arc – BP 87900 – 21079 Dijon Cedex

SYNOPSIS

**Title**	**PRODIGE 54 (FFCD 1603) – SAMCO**<br>**MULTICENTER RANDOMIZED PHASE II STUDY COMPARING THE EFFECTIVENESS AND TOLERANCE OF AVELUMAB VERSUS STANDARD 2nd LINE TREATMENT CHEMOTHERAPY IN PATIENTS WITH COLORECTAL METASTATIC CANCER WITH MICROSATELLITE INSTABILITY (MSI)**<br>**A prospective, multicenter, open-label, randomized, active-controlled, phase 2 study to compare the efficacy and safety of second line treatment with Avelumab versus standard therapy, in patients with MSI-H metastatic COlorectal cancer, the SAMCO trial**
**Sponsor**	Fédération Francophone de Cancérologie Digestive (FFCD) (French-speaking federation of digestive oncology)
**Design**	Multicenter randomized phase II comparative open study
**Trial objectives**	**Principal objective:**<br>Compare between the two treatment arms (arm A: 2nd line chemotherapy, arm B: avelumab) progression-free survival assessed by the investigator according to RECIST v1.1 criteria<br><br>**Secondary objectives:**<br>Time to progression assessed by investigator<br>Overall survival (median)<br>Time to best response<br>Objective Response rate<br>Best response under treatment<br>Toxicity according to NCI-CTC v4.0<br>Secondary resection rate (R0 and R1)<br>Histological response in case of secondary resection (TRG criteria and mTRG)<br>Evolution of tumor markers (CEA)<br>Quality of life QLQ-C30<br><br>By **central review** in RECIST v1.1 and iRECIST criteria:<br>Time to progression<br>Time to best response<br>Objective Response rate<br>Best response under treatment<br>Depth of response<br>Early tumor shrinkage 8 weeks<br>Progression free survival
**Inclusion criteria**	Histologically proven colorectal adenocarcinoma with metastasis(es) non-resectable<br>MSI-H determined by immunohistochemistry (loss of expression of MLH1, MSH2, MSH6 and/or PMS2) and by molecular biology<br>At least one measurable target (primary tumour or metastasis) according to RECIST v1.1<br>Mutational status RAS and BRAF<br>Age ≥ 18<br>WHO ≤ 2<br>Life expectancy ≥ 3 months<br>Patient failure (progression or unacceptable toxicity) of chemotherapy containing fluoropyrimidine (capecitabine or 5FU) +/- irinotecan +/- oxaliplatin with or without cetuximab, bevacizumab ,panitumumab or aflibercept (patients in progression during or within 6 months after discontinuation of adjuvant chemotherapy are eligible)<br>PNN ≥ 1500/mm^3, platelets ≥ 100 000/mm^3, Hb ≥ 9 g/dL<br>Total bilirubin ≤ 25 µmol/L, ASAT ≤ 3 x LSN, ALAT ≤ 3 x LSN (ASAT , ALAT ≤ 5 x LSN in case of hepatic metastasis) , PT >60% , PAL<2.5 x LSN (≤ 5 x LSN in case of hepatic metastasis)<br>Creatinine clearance ≥ 50 ml/min according to MDRD formula<br>Patient belonging to a social security scheme<br>Patient information and signature of the informed consent<br>Tumor block available
**Non-inclusion criteria**	Patient immediately eligible for a curative therapy (surgical and/or percutaneous) after discussion in CPR<br>Patient having progressed under 1st line treatment with FOLFIRINOX or FOLFOXIRI<br>Cerebral metastasis<br>Previous treatment with anti-PD1 or anti-PDL1

	Autoimmune disease that might be aggravated during treatment with an immuno-stimulating agent (patients with type I diabetes, vitiligo, psoriasis, hypo- or hyperthyroid disease not requiring immunosuppressive treatment are eligible)<br>Immunosuppressive long-term treatment (patients necessitating a corticotherapy are eligible if they are administered in doses < or = to the equivalent of 10 mg of prednisone daily, administration of steroids by a route resulting in minimal systemic exposure (local, intra-anal, intraocular or inhalation) are eligible).<br>Transplant patients (including stem cell transplants), HIV positive or other immune deficiency syndromes<br>Active infection by HBV or HCV<br>Known severe hypersensitivity to monoclonal antibodies or history of anaphylactic shock, or uncontrolled asthma<br>Any known specific contraindication or allergy to the treatments used in the study (oxaliplatin, irinotecan, leucovorin, 5-fluorouracil and targeted therapy of choice [bevacuzimab, aflibercept, cetuximab or panitumumab]. In order to check the contraindications, please refer to the updated versions of the SmPCs presented in Appendix 8 of the protocol.<br>Peripheral sensory neuropathy with functional impairment<br>Persistence of toxicities related to 1st line chemotherapy grade $\geq$ 2 (NCI-CTC v4.0) (except alopecia and neuropathy sequelae of oxaliplatin)<br>Vaccination during the 4 weeks preceding the start of treatment<br>QT/QTc interval > 450 msec for men and > 470 msec for women<br>K^+ < LIN, Mg^{2+} < LIN, Ca^{2+} < LIN<br>Following alterations in the 6 months prior to inclusion: myocardial infarction, angina, severe/unstable angina, coronary artery bypass surgery, congestive heart failure NYHA class II, III or IV, stroke or transient ischemic attack<br>Any progressive pathology not stabilised over the past 6 months: hepatic failure, renal failure, respiratory failure<br>Patient with interstitial pneumonitis or pulmonary fibrosis or any other known severe respiratory insufficiency<br>History of inflammatory bowel disease or unresolved occlusion or sub-occlusion in symptomatic treatment<br>History of malignant pathologies during the past 5 years except basocellular skin carcinoma or *in situ* cervical carcinoma, properly treated<br>Patient already included in another clinical trial during treatment with an experimental molecule for L2 or treatment ended in the last 4 weeks before inclusion<br>Lack of effective contraception in patients (men and/or women) of childbearing age, pregnant or breastfeeding women, women of childbearing age not having had a pregnancy test<br>Persons deprived of liberty or under supervision<br>Impossibility of undergoing medical monitoring during the trial for geographic, social or psychological reasons<br>Active tuberculosis<br>Partial or complete DPD deficiency (Uracilemia ≥ 16 ng/ml)
**Study treatment**	**Arm A (reference arm): choice of the investigator**<br>*Chemotherapy*:<br>FOLFIRI (if the patient was treated with FOLFOX in 1st line) or FOLFOX if the patient was treated in 1st line by FOLFIRI and left at the investigator decision if the patient received fluoropyrimidine alone in first line<br>Oxaliplatin: 85 mg/m² IV over 2 hours **OR** Irinotecan: 180 mg/m² IV over 1 hour 30<br>Folinic acid: 400 mg/m² (or 200 mg/m² if Elvorine) IV 2 hours<br>5Fu bolus: 400 mg/m² IV bolus over 10 minutes<br>5Fu continuous: 2400 mg/m² IV over 46 hours<br><br>*+/- targeted treatment at choice of the investigator*<br>Cetuximab: 500 mg/m²<br>Or Panitumumab: 6 mg/Kg<br>Or Bevacizumab: 5 mg/Kg<br>Or Aflibercept: 4 mg/Kg<br>1 treatment every 14 days until unacceptable progression or toxicity or patient refusal<br><br>**Arm B (experimental arm)**<br>Avelumab: 10 mg/Kg<br><br>1 treatment every 14 days until unacceptable progression or toxicity or patient refusal
**Randomization**	Randomization (1:1) of the patient will be done according to a minimization technique and will be stratified according to the following stratification factors:

	Center<br>WHO: 0-1 vs 2<br>BRAF status: non-mutated BRAF vs. mutated BRAF<br>Age : <70 vs ≥70
**Calculating the sample size**	The hypotheses used to calculate the number of subjects necessary are:<br>H_0: The progression-free survival median is not different between the 2 arms.<br>H_1: The progression-free survival median is different between the 2 arms.<br>An improvement of 5 months is expected in favor of arm B (Avelumab) (change from 7 to 12 months, HR = 0.58)<br>Using a fixed design by the Schoenfeld method and considering a bilateral alpha risk of 5% and a power of 80%, 106 events (progression or death) are needed to demonstrate this difference.<br>With an estimated recruitment rate of 3 patients per month, a follow-up period for each patient of 24 months, and a percentage of lost to follow-up of or not evaluable 15%, **132 patients** must be randomized..
**Statistical analysis**	Safety analyses will be done on the ITT population defined as patients randomized whatever eligibility criteria are.<br><br>Analyses of primary and secondary efficacy endpoints will be conducted in the modified intention-to-treat (mITT) population i.e. all CCRm patients with double checked MSI regardless of their eligibility criteria and who have had received at least one dose of treatment in the study. Patients will be analyzed according to treatment received.<br><br>A Per-Protocol (PP) analysis of the primary endpoint will also be done. Per-protocol population is defined as all CCRm patients with double checked MSI with all eligibility criteria, who will receive at least one dose of treatment and who will have at least one tumor evaluation.<br>Safety analyses will also be performed on the modified intention to treat (mITT) population.<br><br>Microbiota ancillary study (stool sampling) with the objective of studying the relationship between the composition of the intestinal microbiota (before treatment and during treatment) and the antitumor response to avelumab or chemotherapy. The results of this study could open new perspectives on the manipulation of the intestinal microbiota (for example: fecal transplantation or microbiota complementation), with the possibility of improving the identification of immune checkpoint inhibitor responders and also of increasing their efficacy and tolerance.
**Ancillary study**	Ancillary study on blood and tumor samples (primary and/or metastasic) to investigate predictive and prognostic factors for response to treatment.<br>As a minimum, this study includes the determination of an immunoscore at tumor tissue level, as well as tumor DNA and RNA analyses and ctDNA analyses (baseline and variation under therapy).<br>The main objective is to generate hypotheses for future biomarkers predictive of response to immune checkpoint inhibitors, notably based on ctDNA (at baseline and ctDNA decrease under treatment) but also on tumor IHC (PD-L1, PD-1, PD-L2, CD8, CD4, CD3, FoxP3), analysis of mutational load and hypermethylated status. in addition to somatic mutations and immunoscore.<br>Microbiota ancillary study (stool sampling) with the aim of studying the relationship between the composition of the intestinal microbiota (before and during treatment) and the antitumor response to avelumab or chemotherapy. The results of this study could open up new prospects for the manipulation of intestinal microbiota (e.g. fecal transplantation or microbiota supplementation), with the potential to improve the identification of responders to immune checkpoint inhibitors, and to enhance their efficacy and tolerability.
**Number of patients**	132 patients
**Duration of inclusion and participation of each patient**	Pace of theoretical inclusions: 3 patients per theoretical month<br>Number of theoretical centers: 50 centers<br>Theoretical beginning of the inclusions: Q3 2017<br>Theoretical end of the inclusions: 39 months after the start of the inclusions, i.e. Q4 2020<br>Theoretical end of the study: Q4 2024

TIMETABLE OF THE EXAMINATIONS AND FOLLOW-UP

	BEFORE TREATMENT	DURING TREATMENT and if treatment is stopped without radiological progression (E.g. toxicity or patient refusal)		AFTER DISCONTINUATION OF THE TREATMENT for radiological progression (failure of strategies)
	During the 15 days preceding the start of treatment	before each course of treatment	Every 8 weeks regardless of the arm	Every 2 to 3 months up to death
**Clinical and biological informed consent**	X			
**CLINICAL EXAMINATION**				
Weight, body area	X	X	X	
Size	X			
General condition WHO	X	X	X	X
Evaluation of toxicity NCI-CTC Version 4.0		X	X and within 30 days after discontinuation of treatment	
QLQ-C30	X		X	
**BIOLOGICAL TESTS**				
Biological test	X*	X***	X*	
Pregnancy test	X		X (every month)	
CEA marker	X		X	X
DPD screening (uracilemia)	X			
**PARACLINICAL REVIEWS**				
Thoraco-abdominal-pelvic CT-scan or MRI	X**		X****	
ECG	X**	X (and at the end of each oxaliplatin infusion)		
**ANCILLARY BIOLOGICAL STUDY**				
2 **STRECK** tubes of 10 ml of blood	X		X	
Biopsies or tumor block, fixed in paraffin	X			
Fecal samples	X	X (Before 3rd administration and at disease progression		
**FUTURE LINES**				
Start and end dates of treatment and the type of treatment of the subsequent lines will be completed in the CRF				X

*: NFS, platelets, PT, sodium, potassium, calcium, magnesium, bilirubin (total and conjugated), GGT, ALT, AST, alkaline phosphatase, LDH, TSH, serum creatinine, creatinine clearance (MDRD - Appendix 4), albumin. For patients treated with aflibercept or bevacizumab: urinary protein (+ protein in 24-hour urine if >2+)
**: During the 3 weeks prior to randomization
*****:** CBC, platelets, creatinine, sodium, potassium, magnesium, AST, ALT, GGT, ALP, total and conjugated bilirubin For patients treated with aflibercept or bevacizumab: urinary protein (+ protein in 24-hour urine if >2+)
******: To achieve until radiological progression**

Send an anonymized copy of the imaging on CD ROM to the FFCD, 7 bd Jeanne d'Arc, BP 87900, 21079 Dijon Cedex (centralised review for principal endpoint and ancillary study)

The study treatment will be stopped in case of decision of the investigator, major toxicity requiring discontinuation of therapy (despite the adaptations provided in the protocol), serious or unexpected event requiring discontinuation of protocol treatment, progression of the disease, patient refusal or withdrawal of consent

TRIAL OBJECTIVES

Main objective

The main objective of this study is to compare between the two arms, of PFS assessed by the investigator according to RECIST v1.1 criteria

Secondary objectives

Time to progression assessed by investigator
Overall survival (median)
Time to best response
Response rate
Toxicity according to NCI-CTC v4.0
Secondary resection rate (R0 and R1)
Histological response in case of secondary resection
Quality of life (QLQ-C30)
Evolution of tumor markers (CEA)

By **central review** in RECIST v1.1 and iRECIST criteria:
Time to progression
Time to best response
Objective Response rate
Best response under treatment
Depth of response
Early tumor shrinkage 8 weeks
Progression free survival

Ancillary biological study on:

Ancillary study on blood and tumor samples (primary and/or metastasic) to investigate predictive and prognostic factors for response to treatment.
As a minimum, this study includes the determination of an immunoscore at tumor tissue level, as well as tumor DNA and RNA analyses and ctDNA analyses (baseline and variation under therapy).
The main objective is to generate hypotheses for future biomarkers predictive of response to immune checkpoint inhibitors, notably based on ctDNA (at baseline and ctDNA decrease under treatment) but also on tumor IHC (PD-L1, PD-1, PD-L2, CD8, CD4, CD3, FoxP3), analysis of mutational load and hypermethylated status. in addition to somatic mutations and immunoscore.
Microbiota ancillary study (stool sampling) with the aim of studying the relationship between the composition of the intestinal microbiota (before and during treatment) and the antitumor response to avelumab or chemotherapy. The results of this study could open up new prospects for the manipulation of intestinal microbiota (e.g. fecal transplantation or microbiota supplementation), with the potential to improve the identification of responders to immune checkpoint inhibitors, and to enhance their efficacy and tolerability.

PATIENT SELECTION FOR REGISTRATION

Inclusion criteria

Histologically proven colorectal adenocarcinoma with metastasis(es) non-resectable
MSI-H determined by immunohistochemistry (loss of expression of MLH1, MSH2, MSH6 and/or PMS2) and by molecular biology
At least one measurable target (primary tumour or metastasis) according to RECIST v1.1
Mutational status RAS and BRAF

Age ≥ 18
WHO ≤ 2
Life expectancy ≥ 3 months
Patient failure (progression or unacceptable toxicity) of chemotherapy containing fluoropyrimidine (capecitabine or 5FU) +/- irinotecan +/- oxaliplatin with or without cetuximab, bevacizumab ,panitumumab or aflibercept (patients in progression during or within 6 months after discontinuation of adjuvant chemotherapy are eligible)
PNN > 1500/mm3, platelets > 100 000/mm3, Hb > 9 g/dL
Total bilirubin < 25 µmol/L, ASAT < 3 x LSN, ALAT < 3 x LSN (ASAT , ALAT < 5 x LSN in case of hepatic metastasis) , PT >60% , PAL<2.5 x LSN (< 5 x LSN in case of hepatic metastasis)
Creatinine clearance > 50 ml/min according to MDRD formula
Patient belonging to a social security scheme
Patient information and signature of the informed consent
Tumor block available

Non-inclusion criteria

patient eligible for curative therapy (surgical and/or percutaneous) after discussion in CPR
Patient having progressed under 1st line treatment with FOLFIRINOX or FOLFOXIRI
Cerebral metastasis
Previous treatment with anti-PD1 or anti-PDL1
Autoimmune disease that might be aggravated during treatment with an immuno-stimulating agent (patients with type I diabetes, vitiligo, psoriasis, hypo- or hyperthyroid disease not requiring immunosuppressive treatment are eligible)
Immunosuppressive long-term treatment. Patients necessitating hormone replacement corticosteroids are eligible if the steroids are administered to target hormone therapy and the dose < or = 10 mg or the equivalent of 10 mg of prednisone by daily administration of steroids by pathway resulting in minimal systemic exposure (local, intra-anal, intraocular or inhalation) are eligible
Transplant patients (including stem cell transplants), HIV positive or other immune deficiency syndromes
Active infection by HBV or HCV
Known severe hypersensitivity to monoclonal antibodies or history of anaphylactic shock, or uncontrolled asthma
Any known specific contraindication or allergy to the treatments used in the study (oxaliplatin, irinotecan, leucovorin, 5-fluorouracil and targeted therapy of choice [bevacuzimab, aflibercept, cetuximab or panitumumab]. In order to check the contraindications, please refer to the updated versions of the SmPCs presented in Appendix 8 of the protocol.
Peripheral sensory neuropathy with functional impairment
Persistence of toxicities related to chemotherapy 1st line ≥ 2 (NCI-CT v4.0) (except alopecia and neuropathy sequelae of oxaliplatin)
Vaccination during the 4 weeks preceding the start of treatment
QT/QTc interval > 450 msec for men and > 470 msec for women
K^+ < LIN, Mg^{2+} < LIN, Ca^{2+} < LIN
Following alterations in the 6 months prior to inclusion: myocardial infarction, angina, severe/unstable angina, coronary artery bypass surgery, congestive heart failure NYHA class II, III or IV, stroke or transient ischemic attack
Any progressive pathology not stabilised over the past 6 months: hepatic failure, renal failure, respiratory failure
Patient with interstitial pneumonitis or pulmonary fibrosis or any other known severe respiratory insufficiency
History of inflammatory bowel disease, or unresolved occlusion or sub-occlusion in symptomatic treatment
History of malignant pathologies during the past 5 years except basocellular skin carcinoma or *in situ* cervical carcinoma, properly treated
Patient already included in another clinical trial with an experimental molecule for L2 or treatment during the last 4 weeks before inclusion
Lack of effective contraception in patients (men and/or women) of childbearing age, pregnant or breastfeeding women, women of childbearing age not having had a pregnancy test
Persons deprived of freedom or under guardianship.
Impossibility of undergoing medical monitoring during the trial for geographic, social or psychological reasons
Active tuberculosis
Partial or complete DPD deficiency (Uracilemia ≥ 16 ng/ml)

INCLUSION REPORT

The inclusion review must be made within 15 days preceding the start of treatment, except the imaging studies, mutational status of the genes RAS and BRAF and ECG, which will be done within 3 weeks prior to the randomization.
. The MSI and MMR IHC status must be obtained by the 2 techniques of assessment (molecular biology or IHC) before randomization.

Clinical examination:
Measurement of weight, height and body surface area
General condition according to WHO scale

Laboratory tests:
CBC, platelets
Total and conjugated bilirubin, ALT, AST, GGT, ALP, PT, LDH,
Serum electrolytes (sodium, potassium, calcium, magnesium), creatinine, creatinine clearance (MDRD - Appendix 4)
Albumin, TSH
For patients treated with bevacizumab or aflibercept: Determination of urinary protein using test strips and, if the result is positive, determination of protein in 24-hour urine.
Marker: CEA
Pregnancy test if woman of childbearing age
DPD deficiency according to the recommendations of INCa and HAS (Opinion n ° 2018.0053 / AC / SEAP of November 28, 2018)

Quality of life questionnaire:
QLQ-C30 to be completed by the patient before randomization (same day or within 15 days of randomization but before the 1st course of treatment)

Determination of RAS and BRAF status
Determination of mutational status of the genes RAS and BRAF. This information is required for randomization (stratification criteria: BRAF status: non-mutated BRAF vs. mutated BRAF)

Morphological examinations and ECG **within 3 weeks prior to randomization:**
Thoraco-abdominal-pelvic scan (TDM TAP or abdominal MRI + thoracic TDM scan without injection if injected scan contraindicated)
ECG

Send an anonymized copy of the imaging on CD ROM to the FFCD, 7 bd Jeanne d'Arc, BP 87900, 21079 Dijon Cedex (centralised review for secondary endpoint and ancillary study)

Ancillary biological study (Appendix 3 and Chapter 8):
Sampling of 2 STRECK tubes of 10 ml of blood before the 1st, before the 3rd cure and at progression.
Retrieving a tumor block or by default 15 thick white blades.

Stool collection before the 1st cure, before the 3rd cure and progression of the disease.

The rationale and logistics of these studies are reciprocally described in Appendix 3 and Chapter 8.

RANDOMIZATION

After signing the consent form and validating the results of the initial baseline assessment, eligible patients will be randomized at the **Randomization - Management - Analysis Center (CRGA) [Centre de Randomisation – Gestion – Analyse] of the FFCD.**

The investigator will fax the completed and signed randomization form with the proof of MSI status to the FFCD RMAC:

Monday to Friday from 9am to 6pm
Fax: + 33 (0)3 80 38 18 41/Tel: + 33 (0)3 80 66 80 13

A randomization confirmation will be faxed back to the investigator and to the pharmacist with the patient registration number and the arm allocated by the randomization.

After randomization of the patient in the study, treatment should begin as soon as possible and within a maximum period of 10 days.

A case report form will be sent when the center opens. A new case report will then be sent after each patient is randomized.

Stratification

The randomization (1:1) of the patients will be done according to the technique of minimization according to the following stratification factors:
Center
WHO: 0-1 vs 2
BRAF status: non-mutated BRAF vs. mutated BRAF
Age : <70 vs ≥70

TRIAL SCHEME

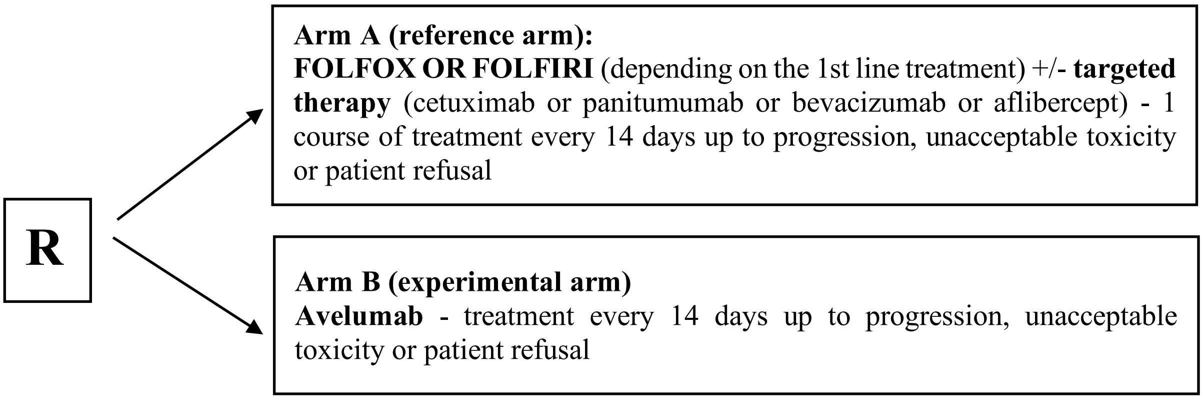

TREATMENTS

6.1 Arm A FOLFOX or FOLFIRI +/- targeted therapy (standard treatment)

The patient will either receive FOLFOX if they received FOLFIRI in 1st line, or FOLFIRI if they received FOLFOX in 1st line and left at the investigator decision if patient has received fluoropyrimidine alone in first line. The investigator will choose whether to administer a targeted therapy such as cetuximab or panitumumab or bevacizumab or aflibercept. The prescription of targeted therapies must be made in the context of their MAs. Please refer to the references to the updated SmPCs in the MAs for the products used for issues of patient management, particularly with respect to contraindications, warnings and precautions for use, dose adjustment in the event of toxicity, monitoring of patients, duration of contraception and medicinal products that are forbidden or to be used with precautions. The links to updated versions of the SmPCs are provided in Appendix 8 of this protocol.

Chemotherapy:

Oxaliplatin: 85 mg/m² IV over 2 hours
OR
Irinotecan: 180 mg/m² IV over 1H30
Folinic acid: 400 mg/m² (or 200 mg/m² if Elvorine) IV over 2 hours
5FU bolus: 400 mg/m² IV bolus over 10 minutes in 100 ml 0.9% NaCl
5FU continuous: 2,400 mg/m² in NaCl 0.9% in IV over 46 hours

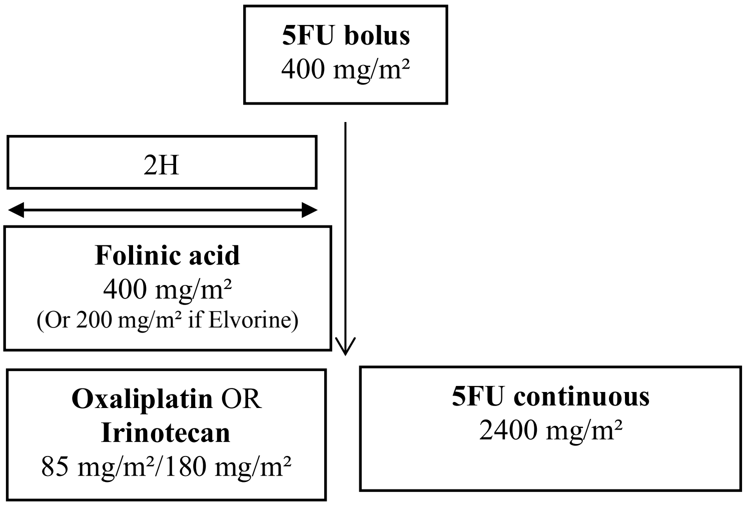

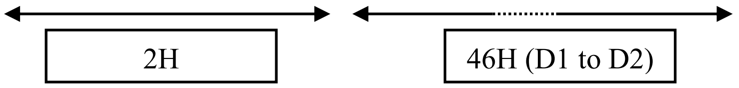

Recommendation on dose capping. The center will perform this according to its usual practices. It may be advisable not to cap the dose at 2 m² if the patient has a large muscular mass. However if the patient has higher fat percentage, a ceiling of 2 m² can be considered.

The treatment and courses of treatments will be repeated until radiological or clinical disease progression according to the investigator, unacceptable toxicity, patient refusal or decision of the investigator.

Targeted therapy :

A targeted therapy can be used in addition to chemotherapy. The choice of targeted therapy is left at the investigator decision and must not be changed during treatment period. It will be administered according to local practice.

Cetuximab:

500 mg/m²over 120 minutes for cycle 1 then 60 minutes for the next cycle in case of good tolerance. A premedication with antihistaminic is recommended in order to reduce risk of allergic reaction or hypersensibility. Dilution in 100 mL NaCl 0.9%

Panitumumab:

6 mg/kg over 60 minutes for the first perfusion then 30 to 60 minutes in case of good tolerance of administration. In case of total dose > 1000 mg, it should be administered over 90 minutes. in a sodium chloride 9 mg / mL (0.9%) solution for injection, the final concentration should not exceed 10 mg / mL

Bevacizumab:

5 mg/Kg over 90 minutes for cycle 1 and in case of good tolerance cycle 2 should be administered over 60 minutes. Next cycles should be administered in 30 minutes in case of good tolerance during cycle 2.

Aflibercept:

Aflibercept will be administered at 4 mg / kg and should be diluted directly in the infusion bag with 0.9% sterile sodium chloride or G5%. Dilute solutions should be administered using infusion sets with a 0.2 micron polyethersulfone filter. Infusion sets should be made of one of the following materials: polyvinyl chloride (PVC) containing bis (2 ethylhexyl) phthalate (DEHP), DEHP-free PVC containing trioctyl trimellitate (TOTM), polypropylene, PVC coated internally Of polyethylene or polyurethane. Note: Filters made of polyvinylidene fluoride (PVDF) or nylon should not be used.

The solution should be prepared in a sterile medium. Aflibercept will be administered within 1 hour. The preparation should not exceed 2 hours at room temperature (25 ° C).

Arm B - Avelumab (experimental arm)

A course of treatment every 14 days.
Premedication obligatory with antihistamines and paracetamol (example: 25-50 mg diphenhydramine and 500-650 mg paracetamol) IV, approximately 30 to 60 minutes before each dose of avelumab.
Then:
Avelumab: 10 mg/kg IV in 1 hour diluted with 0.9% saline solution
The treatment and courses of treatments will be repeated until disease progression, unacceptable toxicity, patient refusal or decision of the investigator.
The treatment will be supplied by Merck Serono until progression and/or unacceptable toxicity, even in the event of premature closure of the trial and even if the trial is negative and some patients are still on avelumab. For patients included with an MSI status determined by a single technique, Avelumab will be provided as described above even if MSI status is not subsequently confirmed by the second technique.

Avelumab can be administered to the chair but with immediate access to intensive care or an equivalent unit (resuscitation equipment) to manage anaphylactic shock.
Treatments to manage these cases should also be accessible quickly (steroids, adrenaline, anti-allergic / antihistamine treatment, bronchodilator (or equivalent), oxygen).

Patients should be followed for 30minute s after the end of the avelumab infusion to detect any reactions related to avelumab infusion.

DOSE ADJUSTMENT ACCORDING TO TOXICITIES

The toxicities requiring dose adjustments will all be evaluated according to the scale NCI-CTCAE v4.0 (Appendix 7) except neurological toxicities to oxaliplatin (paragraph 7.1.2).

Definition of febrile neutropenia: fever occurred in periods of myelosuppression (ANC <500/mm$^{3)}$ with fever > 38.5 °C.

Criteria required before implementation of any new course of treatment

The indication for primary prophylaxis by G-CSF in arm A will be at the discretion of the investigator and according to the hematological toxicity of the first line of treatment and the patient's clinical characteristics. In arm B, prophylactic by G-CSF is not necessary.

Dosage adjustment based on toxicities observed between courses of treatment

Dosage adjustments are needed depending on the maximum grade of toxicity observed between courses of treatment.

The treatments will only be begun when the criteria required before implementation of any new treatment is obtained (see paragraph on toxicities). Dose adjustments are proposed but left at the decision of the investigator.

The occurrence of grade 4 toxicity (excluding haematologic toxicities or other manageable toxicity) shall require the permanent discontinuation of the study treatments unless the investigator considers that there is an interest for the patient to continue with the rest of the treatment when the alleged responsibility of the toxicity observed is not deducted. The recourse treatments will be at the discretion of the investigator. In all cases, the patient will continue to be monitored as part of the protocol according to the protocol pace.

FOLFOX OR FOLFIRI AND TARGETED THERAPIES

<u>Please refer to the references to the updated SmPCs in the MAs for the products used for issues of patient management, particularly with respect to contraindications, warnings and precautions for use, dose adjustment in the event of toxicity, monitoring of patients, duration of contraception and medicinal products that are forbidden or to be used with precautions. The links to updated versions of the SmPCs are provided in Appendix 8 of this protocol.</u>

<u>FOLFIRI</u>

Modification of the dose of FOLFIRI according to the maximum haematological toxicity on the day of treatment

Grade of Toxicity (NCI-CTC)	5-fluorouracil	Irinotecan
Neutropenia, thrombopenia		
2 [a]	- Bolus reduced by 50%	- No modification
3 [a]	- Bolus eliminated, 5FU continuous reduced by 25%	- No modification

4	- Bolus eliminated, 5FU continuous reduced by 25%	- 25% reduction
Febrile neutropenia	- Bolus eliminated, 5FU continuous reduced by 25%	- 25% reduction

[a] Discuss the prescription of G-CSF if neutropenia persists < 1500 after 1 week of treatment delay

Modification of the dose of FOLFIRI according to the maximum toxicity between courses of treatment

Grade of Toxicity (NCI-CTC)	5-fluorouracil	Irinotecan
Neutropenia, thrombopenia		
2 [a]	- No modification	- No modification
3 [a]	- No modification	- No modification
4	- Bolus eliminated,	- No modification
Febrile neutropeniab	- Bolus eliminated, 5FU continuous reduced by 25%	- 25% reduction

[a] Discuss the prescription of G-CSF if neutropenia persists < 1500 after 1 week of treatment delay

Other toxicities

Diarrhea in spite of maximum treatment		
2	- Bolus reduced by 50%	- 25% reduction
3	- Bolus reduced by 50%, 5FU continuous reduced by 50%	- 25% reduction
4	- Discontinuation of chemotherapy	-Discontinuation of chemotherapy
Mucositis		
2	- Bolus reduced by 50%	- No modification
3	- Bolus eliminated, 5FU continuous reduced by 25%	- No modification
4	- Discontinuation of chemotherapy	- Discontinuation of chemotherapy
Vomiting		
3	- 25% reduction in 5FU continuous	- 25% reduction
4	- Discontinuation of chemotherapy	- Discontinuation of chemotherapy
Hand-foot syndrome		
2	- 25% reduction in 5FU continuous	- No modification
3	- Bolus reduced by 50%, 5FU continuous reduced by 50%	- No modification
Non-haematological toxicity apart from alopecia		
3	- 25% reduction in bolus and 5FU continuous	- 25% reduction
4	- Discontinuation of chemotherapy	- Discontinuation of chemotherapy

[a] Discuss the prescription of G-CSF if neutropenia persists < 1500 after 1 week of treatment delay

<u>FOLFOX</u>

<u>Toxicity observed on the day of the course of treatment</u>

	CURE REPORT	DOSE REDUCTION

**Hematologic toxicity on the day of the course of treatment**		**Oxaliplatin**	**5FU bolus:**	**5FU continuous:**
PNN ≥ 1500/ mm ^{3} and platelets ≥ 100,000/mm^3	No course of treatment report	No dose reduction		
PNN < 1500/mm^3	Postpone treatment until a figure is obtained of PNN ≥ 1500/mm^3 (up to D35 if necessary) and resume course of treatment with administration of G-CSF for secondary prophylaxis.<br><br>If no recovery at D35, discuss discontinuation of treatment, growth factor	no dose reduction	1st episode: No dose reduction<br>2nd episode: Discontinuation of bolus and GCSF recommended<br>3rd episode: Discontinuation of bolus and GCSF recommended	1st episode: No dose reduction<br>2nd episode: 100% of 5FU continuous and GCSF recommended<br>3rd episode: 75% of the theoretical dose (i.e. 25% reduction) of 5FU continuous
Isolated febrile neutropenia<br>-Neutropenia grade 4 > 7 days<br>-Infection with neutropenia of grade 3-4 concomitant	Postpone treatment until a figure is obtained of PNN ≥ 1500/mm^3 and infection cured (up to D35 if necessary) and resume course of treatment with G-CSF.<br>If no recovery at D35, discuss discontinuation of treatment, growth factor		1st episode: Discontinuation of bolus and GCSF recommended<br><br>2rd episode: Discontinuation of bolus and GCSF recommended	1st episode: 100% of 5FU continuous and GCSF recommended<br><br>2rd episode: 75% of the theoretical dose (i.e. 25% reduction) of 5FU
Platelets < 100 000/mm^3	Until recovery (platelets ≥ 100 000/mm^3). If no recovery at D35, discuss **discontinuation of treatment**	1st episode: No dose reduction<br><br>2nd episode: dose reduction to 65mg/m^2	1st episode: No dose reduction<br>2nd episode: Discontinuation of 5FU bolus<br>3rd episode: Discontinuation 5FU bolus	1st episode: No dose reduction<br>2nd episode: 100% of 5FU continuous<br>3rd episode: 75% of the theoretical dose (i.e. 25% reduction) of 5FU
Platelets < 50 000/mm^3<br>Thrombopeniab Grade 3-4	Until recovery (platelets ≥ 100 000/mm^3). If no recovery at D35, discuss **discontinuation of treatment**	1st episode: Dose reduction to 65mg/m^2<br><br>2nd episode: Maintaining the reduced dose<br><br>3rd episode: Discontinuation of treatment to be discussed	1st episode: No dose reduction<br>2nd episode: Discontinuation of 5FU bolus<br>3rd episode: Discontinuation of 5FU bolus	1st episode: No dose reduction<br>2nd episode: 100% of 5FU continuous<br>3rd episode: 75% of the theoretical dose (i.e. 25% reduction) of 5FU continuous

Maximum toxicity observed between courses of treatment

**EVENTS**		**REDUCTION RATE TO THE NEXT CURE**	
	Oxaliplatin	5FU bolus	5FU continuous
Isolated febrile neutropenia<br>-Neutropenia grade 4 > 7 days<br>-Infection with neutropenia of grade 3-4 concomitant	No dose reduction of oxaliplatin regardless of the number of episodes	1st episode: Discontinuation of 5FU bolus and GCSF recommended<br>2rd episode: Discontinuation of 5FU bolus	1st episode: 100% of 5FU continuous and GCSF recommended<br>2rd episode: 75% of the theoretical dose (i.e. 25% reduction) of 5FU continuous and GCSF recommended
Thrombopeniab Grade 3-4	1st episode: No dose reduction<br>2nd episode: oxaliplatin decreased to $65mg/m^2$<br>3rd episode: oxaliplatin decreased to $65mg/m^2$	1st episode: No dose reduction<br>2nd episode: Discontinuation of 5FU bolus<br>3rd episode: Discontinuation 5FU bolus	1st episode: No dose reduction<br>2nd episode: No dose reduction<br>3rd episode: 75% of the theoretical dose (i.e. 25% reduction) of 5FU continuous

Gastrointestinal toxicity

**EVENTS**	**REDUCTION RATE TO THE NEXT CURE**	
	**5FU bolus**	**5FU continuous**
- Mucositis of grade 3 isolated diarrhea	**No change to 5FU bolus**	**75% of the dose of 5FU continuous (25% decrease)**
- Mucositis or grade 4 isolated diarrhea<br>- Diarrhea + fever and/or grade 3-4 neutropenia	1st episode: No change of 5FU bolus<br>2nd episode: No change of 5FU bolus<br>3rd episode: discontinuation 5FU bolus	1st episode: 75% of 5FU continuous (reduce the dose of 5FU continuous by 25%)<br>2nd episode: keep the same adaptation of previous dose<br>3rd episode: discontinuation 5FU continuous

In case of occurrence of haemorrhagic gastrointestinal ulceration or not, treatment with 5FU should be discontinued until symptoms disappear.

Other toxicities

"Hand-foot" syndrome: grade 3-4, reduce the 5FU continuous by 25% for the following courses of treatment.

Toxicity specific to oxaliplatin

Peripheral neuropathy

**Toxicity**	**Duration of toxicity**		
	**≤ 7 days**	**> 7 days and**	**Persistent between**

		< 14 days	**Courses of treatment**
Paresthesia/dysesthesia without functional impairment (NCI grade 1)	No modification	No modification	No modification
Paresthesia/dysesthesia with functional impairment but not hindering the activities of daily life (grade 2 NCI)	No modification	No modification	**65 mg/m^2**
Paresthesia/dysesthesia with pain or functional impairment causing problems in the activities of daily life (grade 3 NCI)	**65 mg/m^2**	**65 mg/m^2**	Discontinuation
Paresthesia/dysesthesia persistent, disabling	NA	NA	Discontinuation
Acute dysesthesia Laryngopharyngeal	Extend the duration of the next infusion to 6 hours.<br>Recommendation/Add (if this has not already been done) 1g of Gluconate 1g and 1g of Calcium<br>magnesium sulphate 15 minutes before the oxaliplatin infusion, infusions to be renewed at the end of the oxaliplatin infusion		

If oxaliplatin is discontinued due to neurotoxicity, LV5FU2 will be continued with or without targeted therapy.

Cardiotoxicity

In view of the cardiotoxicity data for oxaliplatin treatment (risk of QTc prolongation, increased risk of ventricular arrhythmia, including potentially fatal torsade de pointes, the QT interval must be strictly and regularly monitored before and after oxaliplatin administration. Patients with a history of or tendency to prolongation of the QT interval, those taking medicinal products known to prolong the QT interval and those with electrolyte imbalances such as hypokalaemia, hypocalcaemia or hypomagnesaemia must be subjected to special monitoring.

In the case of extension of the QT/QTc interval > 500 msec: Discontinuation of treatment by oxaliplatin with close monitoring by ECG and continuously adapted in hospital, until advice of a cardiologist.

BEVACIZUMAB

High blood pressure:

BP taken after at least 5 minutes of rest
if systolic BP ≥ 140mm Hg and/or Diastolic BP ≥ 90 mm Hg, repeat after another 5 minutes of rest

Action to take in the event of HBP:
Grade 1 HBP: asymptomatic, transient (< 24h) up to 150/100 mm Hg
=> *No treatment, continuation of bevacizumab*
Grade 2 HBP: recurrent or persistent (> 24 h) or symptomatic with diastolic BP increased by 20 mm Hg or SBP/DBP > 150/100 mm Hg
=> *Continuation of bevacizumab, antihypertension treatment, monotherapy and do not suspend the anti-angiogenic*
Grade 3 HBP: not controlled by monotherapy (or by double therapy for patients already treated for HBP before being treated with bevacizumab)
=> *Permanent discontinuation of bevacizumab until BP is balanced (PAS/D < 150/100 mm Hg)*
Grade 4 HBP: Life-threatening HBP (hypertensive crisis)
=> *Permanent discontinuation of bevacizumab*

Thromboembolic event

Permanent discontinuation of bevacizumab if an **arterial thromboembolic accident** occurs

In the event of a **venous thromboembolic accident**: suspend bevacizumab for 2 weeks, then restart it after obtaining an effective anticoagulant treatment

Proteinuria

Patients should be tested for urinary protein at enrolment and then after each administration.
If proteinuria ++ or +++ on the urine strip taken before treatment:

- Administer the bevacizumab without altering the dose
- Perform a proteinuria test after 24h before the next cycle:
    - if proteinuria ≤ 2 g/24H: administer bevacizumab without altering the dose
    - if proteinuria > 2 g/24H:
        - do not administer bevacizumab
        - repeat a proteinuria test after 24H before the next cycle and apply:

      the same adaptation rules

- perform a proteinuria test after 24H at each cycle for as long as proteinuria > 1 g/24h

Permanent discontinuation of bevacizumab if nephrotic syndrome

Intestinal perforation

=> *Permanent discontinuation of bevacizumab*

Haemorrhage

Grade 3 or 4

=> *Permanent discontinuation of bevacizumab*

Posterior reversible encephalopathy syndrome (PRES)

It has been reported rarely that patients treated with bevacizumab develop signs and symptoms consistent with Posterior Reversible Encephalopathy Syndrome (PRES), a rare neurological disorder that can manifest itself, among others, by the following signs and symptoms: attacks, headache, altered mental status, visual disturbances, cortical blindness, with or without associated hypertension. The PRES diagnostic requires confirmation by brain imaging, preferably by magnetic resonance imaging (MRI). In patients who develop RPLS, specific treatment of symptoms including control of hypertension, is recommended along with discontinuation of bevacizumab. The consequences in terms of tolerance of a reinitiation of the treatment with bevacizumab in patients who developed SEPR are not known.

Osteonecrosis of the jaw

Osteonecrosis of the jaw has been reported in cancer patients treated with bevacizumab, the majority of which had received prior or concomitant treatment with bisphosphonates administered intravenously, and which had a known risk of osteonecrosis of the jaw. Special attention is recommended in case of prior or concomitant administration of bevacizumab with bisphosphonates administered intravenously. Invasive dental procedures are known to be a risk factor. A dental examination with appropriate preventive dentistry should be considered prior to initiating the treatment with bevacizumab. For patients who have previously received or are receiving treatment with bisphosphonates administered intravenously, invasive dental procedures should be avoided if possible.

PANITUMUMAB

Dermal toxicity

In the event of suspension of administration of panitumumab, continuation of the other treatments

**Development of skin symptoms: grade ≥ 3* (NCI-CTCAE v4.0)**	**Administration of Panitumumab**	**Evolution**	**Dosage adjustment**
1st appearance	Suspend administration	Improvement (grade <3)	Continue the infusions at 100% of the initial dose
		No recovery after 35 days	Discontinuation of treatment
2nd appearance	Suspend administration	Improvement (grade <3)	Continue the infusions at 80% of the initial dose

		No recovery after 35 days	Discontinuation of treatment
At the 3th appearance	Suspend administration	Improvement (grade <3)	Continue the infusions at 60% of the initial dose
		No recovery after 35 days	Discontinuation of treatment
At the 4th appearance	Discontinuation of treatment	-	-

*Grade Reactions ≥ 3 are defined as severe or life-threatening reactions

It is appropriate to suspend or discontinue treatment with panitumumab in case of dermatological or mucosal toxicity, accompanied by severe inflammatory or infectious implications or posing a threat to life.

Preventive treatment of dermatological reactions is systematic. It is recommended to treat patients all along study treatment period in order to reduce treatment interruptions due to severe rash. It combines a moisturiser and sun protection: wearing a hat and limit sun exposure and sunscreen (IP>15 UVA and UVB) and oral antibiotics (e.g. cyclin 100mg). It is allowed to use thick cream, emollient without alcohol. It should be avoided hard detergents and any other local factor that may worsen rashes (friction, traumatism, manipulation…). Treatment of rash/dermatological toxicities leave on dermatocorticoids cream.

Infusion-related reactions

Reduce the infusion rate in patients with a reaction related to a mild or moderate infusion (CTCAE v4.0 grade 1 and 2) for the duration of that infusion. Maintain this decreased infusion rate for all subsequent infusions.
If a reaction that is severe or life-threatening occurs during an infusion or at any time after infusion, panitumumab must be permanently discontinued.

CETUXIMAB

Dermatologic toxicity

Preventive treatment of dermatological reactions is systematic. It is recommended to treat patients all along study treatment period in order to reduce treatment interruptions due to severe rash. It combines a moisturiser and sun protection: wearing a hat and limit sun exposure and sunscreen (IP>15 UVA and UVB) and oral antibiotics (e.g. cyclin 100mg). It is allowed to use thick cream, emollient without alcohol. It should be avoided hard detergents and any other local factor that may worsen rashes (friction , traumatism, manipulation…).Treatment of rash/dermatological toxicities leave on dermatocorticoids cream.

Proposal for topical treatment:
Emollient twice a day on body surface areas where skin rash occurs (e.g.: DEXERYL, CICALFATE)
Hydrocortisone cream and lotion (1% or 2.5%) (e.g. DIPROSONE)

Systematic pre-emptive treatment of cetuximab -induced skin toxicity in both arms with systemic antibiotics such as doxycycline 50 to 200mg/day during 1 or 2 month (e.g.: TOLEXINE 100mg/d) [Jatoi A, Rowland K, Sloan JA et al. Tetracycline to prevent epidermal growth factor receptor inhibitor-induced skin rashes: results of a placebo-controlled trial from the North Central Cancer Treatment Group (N03CB). Cancer 2008 Aug 15;113(4):847-53; Scope A, Agero AL, Dusza SW et al. Randomized double-blind trial of prophylactic oral minocycline and topical tazarotene for cetuximab-associated acne-like eruption. J Clin Oncol 2007 Dec 1;25(34):5390-6]

Antihistamines
Oral prednisone (short term i.e., <14 days treatment) may be added at Investigator’s discretion

**ARM A**	**Dermatologic treatment**	**cetuximab**
Grade 0.1	Topical treatment +/- systemic treatment	Performed as planned
Grade 2	Topical treatment + Systemic treatment	Performed as planned

Grade 2 for ≥ 7 consecutive days<br>Grade 3<br>Patient poor tolerance	1st occurrence	Topical treatment and systemic treatment	Hold infusion until recovery to CTCAE ≤ grade 2.<br>Resume treatment at dose 500mg/m^2
	2nd occurrence	Topical treatment and systemic treatment	Hold infusion until recovery to CTCAE ≤ grade 2 or baseline in the individual treatment course<br>Resume treatment at reduced dose 400mg/m^2
	3th occurrence	Topical treatment and systemic treatment	Hold infusion until recovery to CTCAE ≤ grade 2 or baseline in the individual treatment course<br>Resume treatment at reduced dose 300mg/m^2
	4th occurrence	Topical treatment and systemic treatment	Treatment should be permanently discontinued

<u>Allergic/hypersensitivity reaction</u>

In each case of a hypersensitivity reaction, the investigator should institute treatment measures according to the best available medical practice. Based on previous experience with cetuximab hypersensitivity reactions, the following treatment guidelines may be applicable:

CTCAE grade 1 allergic reaction/hypersensitivity

Description: mild transient reaction (transient flushing or rash, drug fever <38°C)

Treatment: decrease the cetuximab infusion rate by 50% and monitor closely for any worsening. The infusion rate may be reduced by 50% again, but stability limits should not be exceeded.

CTCAE grade 2 allergic reaction/hypersensitivity

Description: flushing, urticaria, dyspnea, drug fever ≥ 38°C and/or bronchospasm, promptly responsive to interruption of infusion and symptomatic treatment.

Treatment:

1) Discontinue cetuximab infusion
2) Administer bronchodilators, oxygen, antihistamines etc. as medically indicated
3) Resume infusion at 50% of previous rate once allergic/hypersensitivity reaction has resolved or decreased to grade 1 in severity, and monitor closely for any worsening. Prolongation of infusion duration should be performed as described for grade 1 reactions.

CTCAE grade 3 or 4 allergic reaction/hypersensitivity

Description:

A grade 3 reaction consists of: symptomatic bronchospasm, requiring parenteral medication(s), with or without urticaria; allergy-related edema/angioedema). Not rapidly responsive to brief interruption of infusion, and/or to symptomatic medication; recurrence of symptoms following initial improvement; hospitalization required.

A grade 4 hypersensitivity reaction is a life-threatening event characterized by rapid onset (often within minutes) of any of the following:

Airway obstruction/respiratory distress (bronchospasm, stridor, hoarseness, difficulty speaking, etc.)

Vascular collapse or shock

Cutaneous manifestations (pruritus, urticaria)

Angioedema

Gastrointestinal manifestations, including dysphagia, cramping, nausea, diarrhea.

A grade 4 hypersensitivity reaction may be complicated by symptomatic hypotension or oxygen saturation of 70% or less.

Treatment:

1) Discontinue cetuximab infusion immediately and disconnect infusion tubing from the patient
2) Administer epinephrine, bronchodilators, antihistamines, glucocorticoids, intravenous fluids, vasopressor agents, oxygen, etc. as medically necessary.

For a CTCAE grade 3 or 4 allergic reaction/hypersensitivity, the patient should not receive further cetuximab treatment.

<u>Electrolytes disorder</u>

Hypomagnesemia

Cetuximab treatment can compromise renal magnesium retention capacity and lead to persistently low serum magnesium levels. Early symptoms of hypomagnesemia are fatigue, paresthesias and muscle cramps. Magnesium can be administered either orally in an oxide, chloride or gluconate form or parenterally as a sulphate salt.

Suggested guidelines for management of hypomagnesemia are as follows (R09-1587):
Grade 1 hypomagnesemia (Mg<LLN-1.2 mg/dl), magnesium chloride starting at 2 tablets PO three times a day, titrating up to 4 tablets PO three times a day as needed.
Investigators may also consider weekly magnesium monitoring without replacement for grade 1 hypomagnesemia in asymptomatic patients without cardiac history or cardiac risks.
Grade 2 hypomagnesemia (Mg<1.2-0.9 mg/dl) weekly intravenous replacement with magnesium sulfate 4 g for patients with magnesium levels of 0.9 to 1.0 mg/dL.
Grade 3/4 hypomagnesemia (Mg<0.9-0.7, Mg<0.7): magnesium sulfate 6 to 10 g IV twice weekly, dependent on the patient. An initial strategy of IV replacement and every-other-day serum magnesium monitoring is helpful to guide the frequency of replacement until a steady state is reached. In a patient with normal renal function start amiloride 5 mg PO daily and titrate up to 10 mg PO daily.

Hypocalcemia

Secondary hypocalcemia is associated with hypomagnesemia.
Correction of the hypomagnesemia usually results in normalization of serum calcium levels.

<u>Pulmonary toxicity</u>

For patients who present acute pulmonary symptoms or worsening of preexisting pulmonary symptoms, investigational treatments should be discontinued until symptoms resolve.
Search for ILD
No retreatment if evidence of ILD

Interstitial pneumonitis

Interstitial lung disease (ILD) events have been reported in patients treated with gefitinib. Up to the present, no increased risk of developing interstitial lung disease has been observed with cetuximab. However, as a precaution, patients should have a CT-scan before the start of cetuximab.
If, a patient presents respiratory symptoms, the investigator will conduct pulmonary function tests and diagnostic work specializing in search of pulmonary fibrosis or underlying interstitial lung disease.
In addition, patients should be regularly examined for signs of lung during the study.

<u>Other toxicities</u>

For any other toxicity Grade ≥ 3 (except alopecia):
Cetuximab
Hold injection until recovery to ≤ grade 1 for cetuximab-related CTCAE
Resume treatment at same dose of 500mg/m² (1st occurrence), 400mg/m² (2nd occurrence), 300mg/m² (3rd occurrence), treatment should be permanently discontinued (4th occurrence)

<u>AFLIBERCEPT</u>

In case of aflibercept-related toxicity, dose adjustments should be made based on the highest grade of toxicity observed according to the NCI-CTCAE version 4.0 (Appendix 6).
If the patient has several toxicities, the adaptation will be done according to the highest toxicity.
Once the dose is decreased, it is not allowed to re-increase it.
Aflibercept will be administered if PNN> 1.500 / mm3 and platelets> 100.000 / mm3 and after recovery to a grade <1 for any other toxicity (excluding alopecia). In case of febrile neutropenia or neutropenic septicemia: in case of recurrence after reduction of the doses of irinotecan and 5FU, it is possible to reduce the dose of aflibercept to 2 mg / kg.

**Toxicities**	**Grade (NCI-CTCAE version 4.0)**	**Management**
**Hypertension**	**Grade < 2**	Start antihypertensive therapy or modify antihypertensive therapy if necessary.<br>No dose modification, no postponement of treatment.
	**Grade 3**<br>(Requiring more than one antihypertensive treatment or requiring intensified antihypertensive therapy)	Repeat administration of FOLFIRI and aflibercept (up to 2 weeks) until recovery of blood pressure <140/90 or PAS <160 if PAD <90 for patients with known history of Isolated systolic hypertension::<br>If arterial pressure (AP) is checked within 2 weeks of postponement<br>1st event: re-administer FOLFIRI and aflibercept at the same dose<br>2nd episode: re-administer FOLFIRI at the same dose and re-administer aflibercept at a dose of 2 mg / kg<br>3rd event: final stop of aflibercept, takeover of FOLFIRI<br>If, after 2 weeks of postponement, AP is still not controlled despite the antihypertensive treatment: resume FOLFIRI at the same dose and stop aflibercept during a FOLFIRI cycle (14 days). Reassess AP at the next cycle and resume aflibercept at 2 mg / kg if AP is controlled.<br>In the case of reappearance of a grade 3 despite optimal antihypertensive treatment and reduction of dose of aflibercept, or if the PA is still not controlled despite the postponement of aflibercept by 2 weeks (4 weeks after last Administration): DEFINITIVE STOP of aflibercept. The FOLFIRI can be continued.
	**Grade 4**	When hypertension is accompanied by symptoms of organ failure such as hypertensive retinopathy, impairment of renal function (such as an increase in proteinuria), symptoms of cardiovascular morbidity or the central nervous system, Aflibercept should be stopped.<br>DEFINITIVE STOP of aflibercept and cardiological advice
**Arterial thromboembolic event**	**Whatever grade**	DEFINITIVE STOP of aflibercept
**Venous thromboembolic event**	**Grade 3**	1st episode: treatment with heparin and continuation of ablibercept[1]<br>2nd episode despite appropriate anticoagulant treatment: DEFINITIVE STOP of aflibercept
	**Grade 4**	DEFINITIVE STOP of aflibercept[2]
**Hemorrhage**	**Grade 3-4**	DEFINITIVE STOP of aflibercept
**Intestinal perforation / Intestinal fistula**	**Whatever grade**	DEFINITIVE STOP of aflibercept
**Syndrome de Reversible**	**Whatever grade**	DEFINITIVE STOP of aflibercept

**posterior leucoencephal a-lopathy syndrome[3]**		

1 After evaluation of the risk of extension and / or embolism according to the judgment of the investigator
2 Continuation of aflibercept may be considered, depending on the benefit / risk balance, in the case of secondary discovery of asymptomatic pulmonary embolism
3 Appearance of vasogenic edema of the white substance predominant in parieto-occipital posterior regions: symptoms: acute and sudden HTA (TAD> 120 mmHg), psychomotor slowdown, headache, confusion, agitation, lethargy, nausea, vomiting, convulsions (Initially focal), transient coma, amnestic disorders, visual disturbances (blurred vision, scintillating scotoma, visual neglect, hemianopsia, cortical blindness)

Proteinuria
A urine test should be performed before each administration of aflibercept (proteins, erythrocytes, leukocytes):
- If proteinuria is <2+ and in the absence of hematuria, aflibercept may be administered
- If proteinuria is> 2+, do not administer aflibercept and reinstate it as soon as 24-hour proteinuria is <2 g. If recurrence of proteinuria> 2 g / 24, aflibercept should be suspended until 24 hours proteinuria <2 g and reintroduced to 2 mg / kg
Aflibercept should be permanently suspended if the patient develops a nephrotic syndrome or thrombotic microangiopathy, suspected of proteinuria-hematuria.

Hypersensitivity reaction to aflibercept

Symptom severity recommendation :
Light and moderate
Example: Grade <2: skin reaction, pruritus, flush, rash, dyspnea, tachycardia, hypotension, anxiety, headache, myalgia, edema, nausea SUSPEND aflibercept infusion
- Administer diphenydramine 50 mg IV and / or dexamethasone 10 mg IV
- Resume aflibercept infusion after recovery

Severe
Example: symptomatic bronchospasm, generalized urticaria, PAS <80 mmHg, angioedema, anaphylaxis STOP aflibercept infusion
- Administer diphenydramine 50 mg IV and / or dexamethasone 10 mg IV and / or epinephrine if necessary
- DEFINITIVE STOP of aflibercept
Unhealed wound / surgery
The half-life of aflibercept is approximately 20 days. Suspend aflibercept at least 4 weeks before surgery.
Aflibercept should be administered at least 4 weeks after surgery and after complete healing.
For small interventions (implantable chamber laying, biopsies, dental extraction), aflibercept can be reintroduced as soon as the wound healing is complete.

Aflibercept should be permanently discontinued if there is a wound opening or non-healing of a wound requiring medical intervention.

AVELUMAB

Table 1 : Treatment Modification for Symptoms of Infusion-Related Reactions

NCI-CTCAE Grade	Treatment Modification for Study Drug
**Grade 1 – mild**<br>Mild transient reaction; infusion interruption not indicated; intervention not indicated.	Decrease the study drug infusion rate by 50% and monitor closely for any worsening.<br>The total infusion time for study drug should not exceed 120 minutes.
**Grade 2 – moderate**<br>Therapy or infusion interruption indicated but responds promptly to symptomatic treatment (for example, antihistamines, NSAIDs, narcotics, IV fluids); prophylactic medications indicated for ≤ 24H.	Stop study drug infusion.<br>Resume infusion at 50% of previous rate once infusion-related reaction has resolved or decreased to at least Grade 1 in severity, and monitor closely for any worsening.
**Grade 3 or Grade 4 – severe or life-threatening**<br>Grade 3: Prolonged (for example, not rapidly responsive to symptomatic medication and/or brief interruption of infusion); recurrence of symptoms following initial improvement; hospitalization indicated for clinical sequelae.<br>Grade 4: Life-threatening consequences; urgent intervention indicated.	Stop the study drug infusion immediately and disconnect infusion tubing from the subject.<br>Subjects have to be withdrawn immediately from study drug treatment and must not receive any further study drug treatment.

IV = intravenous; NCI-CTCAE = National Cancer Institute-Common Terminology Criteria for Adverse Event; NSAIDs = nonsteroidal anti-inflammatory drugs.

If avelumab infusion rate has been decreased by 50% or interrupted due to an infusion reaction, it must remain decreased for the next scheduled infusion. If no infusion reaction is observed in the next scheduled infusion, the infusion rate may be returned to baseline at the subsequent infusions based on investigator's medical judgment.- If hypersensitivity reaction occurs, the subject must be treated according to the best available medical practice. If a subject experiences a Grade 3 or 4 infusion-related reaction at any time, the subject must discontinue study drug.

The following ADRs (adverse drug reaction) require permanent treatment discontinuation of avelumab:

Any Grade 4 ADRs require treatment discontinuation with avelumab except for single laboratory values out of normal range that are unlikely related to study treatment as assessed by the Investigator, do not have any clinical correlate, and resolve within 7 days with adequate medical management

Any Grade 3 ADRs require treatment discontinuation with avelumab except for any of the following:

Transient (≤ 6 hours) Grade 3 flu-like symptoms or fever, which is controlled with medical management

Transient (≤ 24 hours) Grade 3 fatigue, local reactions, headache, nausea, emesis that resolves to Grade ≤ 1

Single laboratory values out of normal range (excluding Grade ≥ 3 liver function test increase) that are unlikely related to study treatment according to the Investigator, do not have any clinical correlate, and resolve to Grade ≤ 1 within 7 days with adequate medical management

Tumor flare phenomenon defined as local pain, irritation, or rash localized at sites of known or suspected tumor

Change in ECOG PS to ≥ 3 that does not resolve to ≤ 2 within 14 days (infusions should not be given on the following cycle, if the ECOG PS is ≥ 3 on the day of study drug administration)

Any Grade 2 ADR should be managed as follows:

If a Grade 2 ADR resolves to Grade ≤ 1 by the last day of the current cycle, treatment may continue.

If a Grade 2 ADR does not resolve to Grade ≤ 1 by the last day of the current cycle, infusions should not be given on the following cycle. If at the end of the following cycle the event has not resolved to Grade 1, the subject should permanently discontinue treatment with a avelumab ADR (except for hormone insufficiencies, that can be managed by replacement therapy; for these hormone insufficiencies, up to 2 subsequent doses may be omitted).

Upon the second occurrence of the same Grade 2 ADR (except for hormone insufficiencies that can be managed by replacement therapy) in the same subject, treatment with avelumab has to be permanently discontinued.

Infusion-related reactions, hypersensitivity reactions (Grades 1 to 4), and tumor lysis syndrome should be handled according to guidelines provided.

Severe Hypersensitivity Reactions and Flu-Like Symptoms
If hypersensitivity reaction occurs, the subject must be treated according to the best available medical practice. Subjects should be instructed to report any delayed reactions to the Investigator immediately.

For prophylaxis of flu-like symptoms, 25 mg of indomethacin or comparable nonsteroidal anti-inflammatory drug (NSAID) dose (for example, ibuprofen 600 mg, naproxen sodium 500 mg) may be administered 2 hours before and 8 hours after the start of each dose of avelumab IV infusion. Alternative treatments for fever (for example, paracetamol) may be given to subjects at the discretion of the Investigator.

Immune-Related Adverse Events

Table 2: Management of Immune-Related Adverse Events

**Gastrointestinal irAEs**		
**Severity of Diarrhea/Colitis (NCI-CTCAE v4.03)**	**Management**	**Follow-up**
**Grade 1**<br>Diarrhea: < 4 stools/day over Baseline<br>Colitis: asymptomatic	Continue avelumab therapy<br>Symptomatic treatment (for example, loperamide)	Close monitoring for worsening symptoms<br>Educate subject to report worsening immediately<br>If worsens:<br>Treat as Grade 2 or 3/4
**Grade 2**<br>Diarrhea: 4 to 6 stools per day over Baseline; IV fluids indicated < 24 hours; not interfering with ADL<br>Colitis: abdominal pain; blood in stool	Withhold avelumab therapy<br>Symptomatic treatment	If improves to Grade ≤ 1:<br>Resume avelumab therapy<br>If persists > 5-7 days or recurs:<br>Treat as Grade 3 or 4.
**Grade 3 to 4**<br>Diarrhea (Grade 3): ≥ 7 stools per day over Baseline; incontinence; IV fluids ≥ 24 hrs; interfering with ADL<br>Colitis (Grade 3): severe abdominal pain, medical intervention indicated, peritoneal signs<br>Grade 4: life-threatening, perforation	Withhold avelumab for Grade 3.<br>Permanently discontinue avelumab for Grade 4 or recurrent Grade 3.<br>1.0 to 2.0 mg/kg/day prednisone IV or equivalent<br>Add prophylactic antibiotics for opportunistic infections<br>Consider lower endoscopy	If improves:<br>Continue steroids until Grade ≤ 1, then taper over at least 1 month; resume avelumab therapy following steroids taper (for initial Grade 3).<br>If worsens, persists > 3 to 5 days, or recurs after improvement:<br>Add infliximab 5mg/kg (if no contraindication).<br>Note: infliximab should not be used in cases of perforation or sepsis.
**Dermatologic irAEs**		
**Grade of Rash (NCI-CTCAE v4)**	**Management**	**Follow-up**
**Grade 1 to 2**<br>Covering ≤ 30% body surface area	Continue avelumab therapy<br>Symptomatic therapy (for example, antihistamines, topical steroids)	If persists > 1 to 2 weeks or recurs:<br>Consider skin biopsy<br>Delay avelumab therapy<br>Consider 0.5 to 1.0 mg/kg/day methylprednisolone IV or oral equivalent. Once improving, taper steroids over at least 1 month, consider prophylactic antibiotics for opportunistic infections, and resume avelumab therapy<br>If worsens:<br>Treat as Grade 3 to 4
**Grade 3 to 4**<br>Covering > 30% body surface area; life threatening consequences	Withhold avelumab for Grade 3.<br>Permanently discontinue for Grade 4 or recurrent Grade 3.<br>Consider skin biopsy<br>Dermatology consult<br>1.0 to 2.0 mg/kg/day	If improves to Grade ≤ 1:<br>Taper steroids over at least 1 month; resume avelumab therapy following steroids taper (for initial Grade 3).

	prednisone or equivalent<br>Add prophylactic antibiotics for opportunistic infections	

Pulmonary irAEs

Grade of Pneumonitis (NCI-CTCAE v4)	Management	Follow-up
**Grade 1**<br>Radiographic changes only	Consider withholding avelumab therapy<br>Monitor for symptoms every 2 to 3 days<br>Consider Pulmonary and Infectious Disease consults	Re-assess at least every 3 weeks<br>If worsens:<br>Treat as Grade 2 or Grade 3 to 4.
**Grade 2**<br>Mild to moderate new symptoms	Withhold avelumab therapy<br>Pulmonary and Infectious Disease consults<br>Monitor symptoms daily; consider hospitalization<br>1.0 to 2.0 mg/kg/day prednisone or equivalent<br>Add prophylactic antibiotics for opportunistic infections<br>Consider bronchoscopy, lung biopsy	Re-assess every 1 to 3 days<br>If improves:<br>When symptoms return to Grade ≤ 1, taper steroids over at least 1 month, and then resume avelumab therapy following steroids taper<br>If not improving after 2 weeks or worsening or for recurrent Grade 2:<br>Treat as Grade 3 to 4.
**Grade 3 to 4**<br>Severe new symptoms; New/worsening hypoxia; life-threatening	Permanently discontinue avelumab therapy.<br>Hospitalize.<br>Pulmonary and Infectious Disease consults.<br>1.0 to 2.0 mg/kg/day prednisone or equivalent<br>Add prophylactic antibiotics for opportunistic infections<br>Consider bronchoscopy, lung biopsy	If improves to Grade ≤ 1:<br>Taper steroids over at least 1 month<br>If not improving after 48 hours or worsening:<br>Add additional immunosuppression (for example, infliximab, cyclophosphamide, IV immunoglobulin, or mycophenolate mofetil)

Renal irAEs

Grade of Liver Test Elevation (NCI-CTCAE v4)	Management	Follow-up
Grade 1<br>Creatinine increased > ULN to 1.5 x ULN	Continue avelumab therapy	Continue renal function monitoring<br>If worsens:<br>Treat as Grade 2 to 3 or 4.
Grade 2 to 3<br>Creatinine increased > 1.5 and ≤ 6 x ULN	Withhold avelumab therapy<br>Increase frequency of monitoring to every 3 days<br>1.0 to 2.0 mg/kg/day prednisone or equivalent.<br>Add prophylactic antibiotics for opportunistic infections<br>Consider renal biopsy	If returns to Grade ≤1:<br>Taper steroids over at least 1 month, and resume avelumab therapy following steroids taper.<br>If worsens:<br>Treat as Grade 4.
Grade 4<br>Creatinine increased > 6 x ULN	Permanently discontinue avelumab Therapy<br>Monitor creatinine daily<br>1.0 to 2.0 mg/kg/day prednisone or equivalent.	If returns to Grade ≤1:<br>Taper steroids over at least 1 month.

	Add prophylactic antibiotics for opportunistic infections<br>Consider renal biopsy<br>Nephrology consult	

**Hepatic irAEs**		
**Grade of Liver Test Elevation (NCI-CTCAE v4)**	**Management**	**Follow-up**
**Grade 1**<br>Grade 1 AST or ALT > ULN to 3.0 x ULN and/or total bilirubin > ULN to 1.5 x ULN	Continue avelumab therapy	Continue liver function monitoring<br>If worsens:<br>Treat as Grade 2 or 3 to 4
**Grade 2**<br>AST or ALT > 3.0 to ≤ 5 x ULN and/or total bilirubin > 1.5 to ≤ 3 x ULN	Withhold avelumab therapy<br>Increase frequency of monitoring to every 3 days.	If returns to Grade ≤ 1:<br>Resume routine monitoring; resume avelumab therapy.<br>If elevation persists > 5 to 7 days or worsens:<br>Treat as Grade 3 to 4.
**Grade 3 to 4**<br>AST or ALT > 5 x ULN and/or total bilirubin > 3 x ULN	Permanently discontinue avelumab therapy<br>Increase frequency of monitoring to every 1 to 2 days<br>1.0 to 2.0 mg/kg/day prednisone or equivalent<br>Add prophylactic antibiotics for opportunistic infections<br>Consult gastroenterologist/ hepatologist<br>Consider obtaining MRI/CT scan of liver and liver biopsy if clinically warranted	If returns to Grade ≤ 1:<br>Taper steroids over at least 1 month<br>If does not improve in > 3 to 5 days, worsens<br>or rebounds:<br>Add mycophenolate mofetil 1 gram (g) twice<br>daily<br>If no response within an additional 3 to 5 days, consider other immunosuppressants per local guidelines.

**Cardiac irAEs**		
**Myocarditis**	**Initial Management**	**Follow-up Management**
New onset of cardiac signs or symptoms and / or new laboratory cardiac biomarker elevations (e.g. troponin, CK-MB, BNP) or cardiac imaging abnormalities suggestive of myocarditis.	Withhold avelumab therapy.<br>Hospitalize.<br>In the presence of life threatening cardiac decompensation, consider transfer to a facility experienced in advanced heart failure and arrhythmia management.<br>Cardiology consult to establish etiology and rule-out immune-mediated myocarditis.<br>Guideline based supportive treatment as per cardiology consult.*<br>Consider myocardial biopsy if recommended per cardiology consult.	If symptoms improve and immune-mediated etiology is ruled out, re-start avelumab therapy.<br>If symptoms do not improve/worsen, viral myocarditis is excluded, and immune-mediated etiology is suspected or confirmed following cardiology consult, manage as immune-mediated myocarditis.
Immune-mediated myocarditis	Permanently discontinue avelumab.<br>Guideline based supportive treatment as appropriate as per cardiology consult.*<br>1.0 to 2.0 mg/kg/day prednisone or equivalent<br>Add prophylactic antibiotics for opportunistic infections.	Once improving, taper steroids over at least 1 month.<br>If no improvement or worsening, consider additional immunosuppressants (e.g. azathioprine, cyclosporine A).

Endocrine irAEs		
**Endocrine Disorder**	**Management**	**Follow-up**
Grade 1 or Grade 2 endocrinopathies (hypothyroidism, hyperthyroidism, adrenal insufficiency, type I diabetes mellitus)	Continue avelumab therapy<br>Endocrinology consult if needed<br>Start thyroid hormone replacement therapy (for hypothyroidism), anti-thyroid treatment (for hyperthyroidism), corticosteroids (for adrenal insufficiency) or insulin (for Type I diabetes mellitus) as appropriate.<br>Rule-out secondary endocrinopathies (i.e. hypopituitarism / hypophysitis)	Continue hormone replacement/suppression and monitoring of endocrine function as appropriate.
Grade 3 or Grade 4 endocrinopathies (hypothyroidism, hyperthyroidism, adrenal insufficiency, type I diabetes mellitus)	Withhold avelumab therapy<br>Consider hospitalization<br>Endocrinology consult<br>Start thyroid hormone replacement therapy (for hypothyroidism), anti-thyroid treatment (for hyperthyroidism), corticosteroids (for adrenal insufficiency) or insulin (for type I diabetes mellitus) as appropriate.<br>Rule-out secondary endocrinopathies (i.e. hypopituitarism / hypophysitis)	Resume avelumab once symptoms and/or laboratory tests improve to Grade ≤ 1 (with or without hormone replacement/suppression).<br>Continue hormone replacement/suppression and monitoring of endocrine function as appropriate.
Hypopituitarism/Hypophysitis (secondary endocrinopathies)	If secondary thyroid and/or adrenal insufficiency is confirmed (i.e. subnormal serum FT4 with inappropriately low TSH and/or low serum cortisol with inappropriately low ACTH) :<br>Refer to endocrinologist for dynamic testing as indicated and measurement of other hormones (FSH, LH, GH/IGF-1, PRL, testosterone in men, estrogens in women)<br>Hormone replacement/suppressive therapy as appropriate<br>Perform pituitary MRI and visual field examination as indicated<br><br>If hypophysitis confirmed:<br>Continue avelumab if mild symptoms with normal MRI.<br>Repeat the MRI in 1 month<br>Withhold avelumab if moderate, severe or life-threatening symptoms of hypophysitis and/or abnormal MRI. Consider hospitalization. Initiate corticosteroids (1 to 2 mg/kg/day prednisone or equivalent) followed by corticosteroids taper during at least 1 month.	Resume avelumab once symptoms and hormone tests improve to Grade ≤ 1 (with or without hormone replacement).<br>In addition, for hypophysitis with abnormal MRI, resume avelumab only once shrinkage of the pituitary gland on MRI/CT scan is documented.<br>Continue hormone replacement/suppression therapy as appropriate.

	Add prophylactic antibiotics for opportunistic infections.	
**Other irAEs (not described above)**		
**Grade of other irAEs (NCI-CTCAE v4)**	**Initial Management**	**Follow-up Management**
Grade 2 or Grade 3 clinical signs or symptoms suggestive of a potential irAE	Withhold avelumab therapy pending clinical investigation	If irAE is ruled out, manage as appropriate according to the diagnosis and consider re-starting avelumab therapy<br>If irAE is confirmed, treat as Grade 2 or 3 irAE.
Grade 2 irAE or first occurrence of Grade 3 irAE	Withhold avelumab therapy<br>1.0 to 2.0 mg/kg/day prednisone or equivalent<br>Add prophylactic antibiotics for opportunistic infections<br>Specialty consult as appropriate	If improves to Grade ≤ 1:<br>Taper steroids over at least 1 month and resume avelumab therapy following steroids taper.
Recurrence of same Grade 3 irAEs	Permanently discontinue avelumab therapy<br>1.0 to 2.0 mg/kg/day prednisone or equivalent<br>Add prophylactic antibiotics for opportunistic infections<br>Specialty consult as appropriate	If improves to Grade ≤ 1:<br>Taper steroids over at least 1 month.
Grade 4	Permanently discontinue avelumab therapy<br>1.0 to 2.0 mg/kg/day prednisone or equivalent and/or other immunosuppressant as needed<br>Add prophylactic antibiotics for opportunistic infections<br>Specialty consult.	If improves to Grade ≤ 1:<br>Taper steroids over at least 1 month
Requirement for 10 mg per day or greater prednisone or equivalent for more than 12 weeks for reasons other than hormonal replacement for adrenal insufficiency<br>Persistent Grade 2 or 3 irAE lasting 12 weeks or longer	Permanently discontinue avelumab therapy<br>Specialty consult	

Abbreviations: ACTH=adrenocorticotropic hormone; ADL=activities of daily living; ALT=alanine aminotransferase; AST=aspartate aminotransferase; BNP=B-type natriuretic peptide; CK-MB=creatine kinase MB; CT= computed tomography; FSH=follicle-stimulating hormone; GH=growth hormone; IGF-1=insulin-like growth factor 1; irAE=immune-related adverse event; IV=intravenous; LH=luteinizing hormone; MRI=magnetic resonance imaging; NCI-CTCAE=National Cancer Institute-Common Terminology Criteria for Adverse Events; PRL=prolactin;T4=thyroxine; TSH=thyroid-stimulating hormone; ULN=upper limit of normal.

PREMEDICATION, CONCOMITANT TREATMENTS AND CONTRAINDICATED TREATMENTS

Treatments considered to be necessary for the patient's well-being can be administered at the investigator's discretion (anti-emetic, anti-diarrhoeic etc.).

The indication for primary prophylaxis with G-CSF in arm A (FOLFOX or FOLFIRI and targeted therapy) will be at the discretion of the investigator, L1 and according to the patient's clinical characteristics.

In case of severe neutropenia, i.e. grade 3-4, patients are at high risk of febrile neutropenia and infection especially in the case of concomitant diarrhea. If these symptoms appear, dosage adjustments are planned in the next course of treatment, and the prescription of hematopoietic growth factors should be considered.

Contraindicated treatment (see SPC of the various molecules of the protocol)

With 5FU: yellow fever vaccine, attenuated live vaccine, prophylactic phenytoin. When combined with warfarin more frequent monitoring of INR

With oxaliplatin: all drugs known to prolong the the QTc interval should be used with caution (list on the following website: https://crediblemeds.org/oncosupport/). Drugs that may be associated with rhabdomyolysis.

With avastin: precautions of use with anticoagulant and anti-aggregation
With panitumumab/cetuximab: none

Avelumab: (cf. BI): Vaccination within 4 weeks preceding the start of treatment and throughout the avelumab treatment is prohibited except the administration of inactivated vaccines. Corticosteroids, immunosuppressive.

Irinotecan: contraindication in cases of combination with St. John's wort.

Conditions for discontinuation of treatment

Treatment may be discontinued if the investigator considers this to be necessary, in a case of major toxicity, which does not permit the treatment to be continued, a serious or unexpected event requirement treatment to be discontinued, disease progression, withdrawal of consent, patient lost to view, refusal by patient, pregnancy. In all cases where it is possible (apart from loss to view or withdrawal of consent), the treatment will be discontinued but the patient will continue to be followed-up as part of the protocol.

LOGISTICS OF THE BIOLOGICAL STUDY

For patients who signed the biological informed consent, the details of the biological ancillary study (circulating DNA and tumor sample) is in Appendix 3 of this protocol.

Samples needed

- A sampling of 2 STRECK tubes of 10 ml of blood before the 1st course of treatment and at the first evaluation, i.e. 8 weeks after the randomization.

The STRECK tubes will be used for extracting the DNA from the plasma (circulating tumor DNA) + buffy coat (genetic polymorphism)

Sending tubes, via the DHL box supplied at opening of the center:

Biological Resource Center EPIGENETEC
Unit UMR-S 1147
45 rue des Sts Pères, 75006 PARIS
Directed by Prof. Pierre LAURENT-PUIG

Use only the DHL box containing the DHL form **addressed to the unit INSERM U775.**

After sending this box, the box needed at inclusion of the next patient will be sent by EPIGENETEC.

- Tumor block fixed in paraffin.

Sending blocks or slides, via the max letters provided at opening of the center to:

Biological Resource Center EPIGENETEC
Unit UMR-S 1147

45 rue des Sts Pères, 75006 PARIS
Directed by Prof. Pierre LAURENT-PUIG

In case of questions or logistic problems, contact Claire MULOT at 01 42 86 38 61, claire.mulot@parisdescartes.fr.

Stool samples for the Microbiota study :
This study is intended for patients who have signed the specific consent for the microbiota study.

A sample is collected by patients before the first cure, before the 3rd cure and after disease progression. Kits will be supplied to the centre containing a procedure for carrying out the sampling. The stool samples will be sent to the INRA-MetaGenoPolis structure.

PATIENT MONITORING

Before each administration of treatment

Clinical examination:
Pulse, TA Temperature
Evaluation of toxicity from the preceding cycle according to NCI-scale CT v4.0
Biological tests: CBC, platelets, bilirubin (total and conjugated), ALP, AST, ALT, sodium, potassium, creatinine, creatinine clearance (MDRD).
Determination of urinary protein using test strips and, if the result is positive, determination of protein in 24-hour urine
Pregnancy test every month if the woman is of reproductive age.

Evaluation every 8 weeks

The patients will be evaluated every 8 weeks with:

Clinical examination:
Weight, WHO
Evaluation of toxicity from the preceding cycle according to NCI-scale CTCAE v4.0
Quality of life questionnaire QLQ-C30 version 3.0

Biological tests: CBC, platelets, bilirubin (total and conjugated), PT, ALP, AST, ALT, sodium, magnesium, potassium, calcium, creatinine, creatinine clearance (MDRD), albumin, LDH, TSH

Marker: CEA

Morphological evaluation: TDM - TAP or MRI if contraindication to injected TDM.
Send an anonymized copy of the imaging on CD ROM to the FFCD, 7 bd Jeanne d'Arc, BP 87900, 21079 Dijon Cedex (centralised review for primary, secondary endpoint and ancillary study)

After discontinuation of the treatment:

Within 30 days

To assess the tolerance of the last course of treatment:
Biological tests: CBC, platelets, bilirubin (total and conjugated), PT, ALP, AST, ALT, sodium, magnesium, potassium, calcium, creatinine, creatinine clearance (MDRD), albumin, LDH, TSH
Evaluation of toxicity (NCI-CTCAE v4.0) of the last cycle of treatment

After premature discontinuation for reason other than radiological progression*

Patients will be followed up according to the same methods **every 8 weeks** until clinical or radiological progression:

Clinical examination: weight, WHO
Evaluation of persistent toxicities (including neuropathy) up to progression
TDM-TAP (or MRI)
Send an anonymized copy of the imaging on CD ROM to the FFCD, 7 bd Jeanne d'Arc, BP 87900, 21079 Dijon Cedex (centralised review for secondary endpoint and ancillary study)
Quality of life questionnaire QLQ-C30 version 3.0
CEA markers

** toxicity, withdrawal of consent, loss of sight, patient refusal, investigator's decision*

Patients of childbearing age and their spouses must agree to use effective contraception without interruption for the duration of the treatment and 6 months after administration of the last dose of treatment

After radiological progression

After radiological progression, patients will be followed according to the local practice until death but at least every 3 months. Realisation of complementary examinations is at the investigator decision.

Patients of childbearing age and their spouses must agree to use effective contraception without interruption for the duration of the treatment and 6 months after administration of the last dose of treatment

1ST LINE TREATMENTS AND SUBSEQUENT TREATMENTS

The 1st line treatments and subsequent treatments will be collected in the CRF. For each line, the following information will be collected: type of chemotherapy, number of cycles and start date of chemotherapy and progression.

MANAGEMENT OF SERIOUS ADVERSE EVENTS (SAE)

Safety evaluation parameters
Safety evaluation is via evaluation of the patients' general clinical and biological condition at the consultations and by recording any events occurring between visits. Toxicity will be evaluated using the NCI-CTCAE toxicity scale Version 4.0 (see Appendix 6).
In an emergency, the patient or family or referring physician must call the investigator to report an event.

Definitions
Adverse Event (AE)
An adverse event is a harmful event occurring to a person involved in biomedical research, whether or not this event is linked to the research or the product with which it is concerned.

All adverse events will be reported in the case report in the pages provided for the purpose.

Serious Adverse Event (SAE)
Considered a serious adverse event is any event that corresponds to at least one of the following criteria:
Leading to death
Life-threatening
Leading to hospitalization or extending a period of hospitalization
Leading to permanent disability or serious temporary incapacity
Leading to congenital anomaly, foetal malformation or abortion
Medically significant.

The terms “disability” and “incapacity” cover any temporary or permanent physical or mental handicap which is clinically significant and affects the patient's physical activity and/or quality of life.

Considered as medically significant is any clinical event or laboratory result considered to be serious by the investigator and which does not meet the severity criteria defined above. They may represent a risk to the patient and require medical intervention to prevent the development of one of the severity criteria mentioned above (e.g. overdose, secondary cancers, pregnancies and new events which can be considered to be medically significant)

Pregnancy is a criterion for non-inclusion in this trial and contraceptive measures must be taken throughout the treatment and up to 6 months after treatment. However, if a pregnancy is discovered after inclusion in a patient participating in the trial, this latter will be excluded from the trial. The Sponsor must be informed without delay via the serious adverse event notification sheet (no severity criterion will be ticked in this case). The patient must be monitored until the end of the pregnancy and, whatever the result, it must be reported to the Sponsor. Similarly, if a pregnancy occurs in a partner of a patient included in the trial, the proponent will be informed in the same way and will try, as much as possible, to see the pregnancy to term.

Adverse Event
Any harmful and undesirable reaction to an experimental medicinal product, no matter what the administered dose, or to an experimental element. An adverse reaction is serious if it meets a severity criterion.

Unexpected Serious Adverse Event
An unexpected serious adverse reaction is an event which is not mentioned, or differing in nature, intensity or evolution from the product reference document (or SPC).

New fact
A new fact may be: an unexpected frequency of an expected SAE, an SAE linked to the trial procedure, an inadequate efficacy in life-threatening diseases, or clinical data.

Intensity (or severity)
The intensity criterion must not be confused with the severity criterion which acts as a guide for defining the declaration requirements.
The intensity of the events will be estimated according to the extract of CTC-AE classification Version 4.0 (see Appendix 6). The intensity of the adverse events not listed in this classification will be assessed according to the following qualifiers:
Mild (grade 1): does not affect the patient's daily activity
Moderate (grade 2): disturbs the patient's normal daily activity
Severe (grade 3): prevents the patient's normal daily activity
Very severe (grade 4): requires reanimation measures/is life-threatening
Death (grade 5)

Causality relation
Related: an event is said to be related when a causality relationship between the event and the study product can reasonably be suspected
Unrelated: an event is said to be unrelated when a causality relationship between the event and the study product cannot reasonably be suspected
Doubtful: causality is said to be "doubtful" when there is doubt as to the causality relationship between the event and the study product (a relationship cannot be formally excluded or formally confirmed)

Liability of the Sponsor
On reception of the serious adverse event declaration filled out by the investigator, the Sponsor must issue a decision on the causality relationship between the serious adverse event and the study product(s).
If the serious adverse event is related to one of the study products by the investigator and/or Sponsor (it is therefore a serious adverse event), it must be established whether this event was expected or unexpected.
If it is an unexpected serious adverse event or a new fact, the Sponsor drafts an initial report to be sent to the ANSM, CPP and EMA (via Eudra Vigilance) within 7 days in the event of death or life-threatening effects, or 15 days otherwise.
If it is an expected serious adverse event, it will be collated with a view to drafting half-yearly reports and annual safety reports.

Events not considered serious

Radiological progression without clinical consequences of the disease should not be considered an SAE.

Events potentially related to progression and that can also be secondary to the treatment shall continue to be reported (e.g. thromboembolic events, haemorrhagic phenomena, perforations, sub-occlusion, occlusion, etc.)

Due to the severity of the disease involved in this trial, certain conditions defined as SAEs will be excluded from the SAE declaration procedure, these being:

Hospitalization or surgery linked specifically to treatment of the disease. However, hospitalization or prolongation of hospitalization for a complication of such treatments should be reported as SAE.

Hospitalization to simplify the study treatments or procedures

In this trial, the reference documents are:

For oxalipatin, the Elvorine® Summary of Product Characteristics (appendix 7)
For irinotecan acid, the Campto® Summary of Product Characteristics (appendix 7)
For 5-fluorouracil, the Fluorouracil TEVA® Summary of Product Characteristics (appendix 7)
For folinic acid, the Elvorine® Summary of Product Characteristics (appendix 7)
For panitumumab, the Vectibix® Summary of Product Characteristics (appendix 7)
For bevacizumab, the Avastin® Summary of Product Characteristics (appendix 7)
For cetuximab, the Erbitux® Summary of Product Characteristics (appendix 7)
For aflibercept, the Zaltrap® Summary of Product Characteristics (appendix 7)
For avelumab, the updated investigator brochure will be used. The document and the updates will be provided to the centers.

The versions of the SPCs used for the definition of expected or unexpected character are those in effect at the time of the analysis.
NB: Note that for 5-fluorouracil, venous thromboembolic events will be regarded as expected although not listed in the SPC.

Procedure to follow

The investigator informs the Sponsor of all serious adverse events (expected and unexpected) whether they are imputable to the research or not, which take place during the study or within 30 days of the last treatment administration.

All delayed serious adverse events (occurring after this 30 day period) reasonably considered to be linked to the protocol treatment(s) or research must be declared with no time limit.

The declaration is made by submitting the "notification of a serious adverse event" sheet (see Appendix 9) documented as fully as possible, dated and signed, within 24 working hours following their observance, to the **FFCD Centre de Randomisation-Gestion-Analyse (CRGA) by fax at 03 80 38 18 41**

The investigator is responsible for ensuring appropriate patient follow-up until resolution or stabilization of the event or the patient's death. This may sometimes mean that this follow-up is extended after the patient has withdrawn from the trial.

He/she sends further information to the Sponsor on the SAE declaration form (ticking the “Follow-up” box and incrementing the number in order to specify that it is a follow-up report and not an initial report) within 24 hours of obtaining the information. He/she also includes the latest follow-up on the resolution or stabilization of the SAE.
He/she answers requests for further information in order to document the initial observation.

STATISTICAL ANALYSIS

Judgement criteria

Main efficacy criterion

The primary endpoint is radiographic progression-free survival (PFS). The progression will assessed by the investigator according to RECIST v1.1 criteria in arm A and B, PFS is defined by the time between the date of

randomization and the date of the first radiological progression or the date of death (for whatever reason). Patients alive without radiological progression will be censored on the date of their last CT-scan.

Secondary criteria

For all secondary endpoints, radiological responses will be evaluated by investigator according to the RECIST v1.1 criteria in arm A and B.

In **central review,** the following sensitivity analyses will also be evaluated in different ways:

- according to the RECIST v1.1 criteria in arm A and B.
- according to iRECIST in arm A and B

The secondary endpoints are:

Time To Progression (TTP):
This time is defined by the time between the date of randomization and the date of the first radiological progression. Patients alive or dead without radiological progression will be censored on the date of their latest CT-scan.

Overall survival (OS):
OS is defined by the time between the date of randomization and the date of death (regardless of the cause). Alive patients will be censored at the date of their last news.

Objective Response Rate :
Objective Response rate is defined by patients with partial or complete response.

Time to Best Response (TBR)
This time is defined as the time from the date of randomization and the date of best response under treatment. Patients without imaging (better response non-evaluable, untreated patients) will not be taken into account in the analysis.

The best response under treatment:
The best tumor response will be evaluated throughout the treatment. The response is evaluated according to the various categories: complete, partial, stability, progression or non-evaluable response.

Toxicities:
Toxicity will be evaluated according to NCI-CTC v4.0.

Early tumor shrinkage at 8 weeks:
This endpoint is defined as the relative difference between the sum of the largest diameters of target lesions at 8 weeks and this sum at baseline.
Early decrease corresponds to a relative difference of > 20% and > 30% in RECIST v1.1.

Depth of response:
This criterion is defined as the relative difference between the sum of the largest diameters of target lesions in the NADIR (in the absence of new lesions or progression of non-target lesions) and the sum of the largest diameters of the target lesions at inclusion.

Secondary resection rate:
This rate is defined as the proportion of patients who could benefit from surgery of their metastases (optionally combined with a surgery of the primary tumor) during 2nd line treatment.

Histological response if resection:
This endpoint will be evaluated according to the TRG (Rubbia-Brandt L et al. Annals Oncol 2007), in patients who underwent a secondary resection of their metastases (possibly associated with surgery of the primary tumor). This response is evaluated according to the various categories: TRG1/TRG 2/TRG 3/TRG 4/TRG 5.

Evolution of CEA markers:
The markers will be collected at each treatment cycle. The evolution of the markers will be analysed by a graphic representation of the percentage change from baseline rate.

Quality of life:

Quality of life will be assessed according to the questionnaire of EORTC QLQ-C30.

Progression free survival

Calculating the number of subjects required, statistical hypotheses

The hypotheses used to calculate the number of subjects necessary are:

H_0: The progression-free survival median is not different between 2 arms.
H_1: The progression-free survival median is different between 2 arms. An improvement of 5 months is expected in favor of arm B (Avelumab) (change from 7 to 12 months, HR = 0.58)

Using a fixed design by the Schoenfeld method and considering a bilateral alpha risk of 5% and a power of 80%, 106 events (progression or death) are needed to demonstrate this difference.
With an estimated recruitment rate of 3 patients per month, a follow-up period for each patient of 24 months, and a percentage of lost to follow-up or not evaluable of 15%, **132 patients** must be randomized.

Analysis populations and analyses performed:

safety analyses will be done on the ITT population defined as patients randomized whatever eligibility criteria are.

Analyses of primary and secondary efficacy endpoints will be conducted in the modified intention-to-treat (mITT) population i.e. all CCRm patients with double checked MSI regardless of their eligibility criteria and who have had received at least one dose of treatment in the study. Patients will be analyzed according to treatment received.

A Per-Protocol (PP) analysis of the primary endpoint will also be done. Per-protocol population is defined as all CCRm patients with double checked MSI fulfilling all eligibility criteria who will receive at least one dose of treatment and who will have at least one tumor evaluation.
Safety analyses will also be performed on the modified intention to treat (mITT) population.

Statistical analyses:

Baseline characteristics will be presented by treatment arm and in the overall population on mITT population.

The ***quantitative*** variables will be described by the usual statistics: mean, standard deviation, median, interquartile range, minimum and maximum. They can also be categorised according to cut-offs of the medical literature.

The ***qualitative*** variables will be described using number and percentages.
Comparisons by treatment arm will be performed for the quantitative variables, using a Student or Wilcoxon test (according to the distribution of the variable) and for qualitative variables, using a chi^2 test or a Fisher exact test.

The ***survival endpoint*** will be estimated and plotted using the Kaplan-Meier estimator (Kaplan and Meier, 1958). The median time and the rates at different times will be described with their 95% confidence interval.
Median follow-up time will be calculated using the reverse Kaplan-Meir method.
Comparisons by treatment arm will be conducted using the log-rank test.
The hazard ratio for the treatment effect will be calculated using a Cox model (Cox, 1984). Log-linearity assumptions and risk proportionality will be checked graphically thanks to residuals (Schoenfeld and Martingale residuals).

Safety analyses:

The dose received and the percentages of actual dose received over theoretical dose, as well as the percentage of patients with at least one dose modification or at least one administrative report will be described by treatment arms.

Toxicities will be described by treatment arm with the number and percentage of patients according to the various grades (grade 1-2 versus grade 3-4-5) by types of toxicities (SOC: System Organ Class) ad Preferred-term (PT). A SAE report will be provided by pharmacovigilance department.

STUDY COMMITTEES

Independent committee

An independent committee will be set up, including at least two gastro-oncologists, a statistician or methodologist, and a pharmacovigilance expert.
The committee will meet at least once a year or more often if the Sponsor deems it necessary in view of the analysis of SAEs. The independent committee may also meet at any time during the protocol if the Sponsor judges this to be necessary.
The committee will decide on all the tolerance data transmitted from the centers to the Sponsor (SAEs +/- adverse events). Assessed will be all the patients included in the study up to 2 months before the date of the meeting of the Independent Committee.

Steering committee

A Steering Committee will be set up. The chairman of the Steering Committee of the study will be the coordinator of the study. This committee will also include the co-coordinators, the FFCD project manager of the study, a statistician of the FFCD, and the President of Biological Research Committee. Its mission will be, among others, to take decisions related to the management of the research (amendment, premature closure if necessary etc.). The committee will meet as often as necessary throughout the study. The Steering Committee will take the necessary decisions on substantial amendments to the protocol, closure or extension of the study.

Medical journal

A medical journal will be established to improve the quality of the collected clinical data. In case of discrepancy between the data provided by the investigator and those in the medical journal, requests for clarification will be sent to the investigator by data management.

Biological research committee

A Biological Research Committee will be established and its mission will be to answer questions related to samplings and their sample bank and the organization of their analysis. The committee will meet regularly and report its proposals to the Steering Committee. This committee will include among others the coordinator of the study and a biologist; the chairman of this committee will be Professor Pierre LAURENT PUIG.

BASIC INFORMATION AND JUSTIFICATION FOR THE STUDY

Human tumors escape immunosurveillance in order to progress and one of the major mechanisms is the activation of immune system regulatory checkpoints. PD-L1 (Program Death - Ligand 1) expression by tumors is the most well-known example as PD-L1 is upregulated on a wide range of cancer cells. Interaction between PD-1 and PD-L1 will lead the activated CD8+ T cell to a state of anergy. Blocking the immunological checkpoints mediated by PD-1 has recently emerged as a highly promising option for the treatment of an ever-increasing number of malignancies, including melanoma, non-small cell lung carcinoma, bladder carcinoma, hodgkin lymphoma, triple-negative breast carcinoma, as well as head and neck cancer (Sharma P, Allison JP. Cell 2015). In fact, anti-PD1 and anti-PD-L1 monoclonal antibodies (mAbs), called immune checkpoint inhibitors (ICIs), have consequently been designed to restore T cell activity. Only a fraction of individuals with these neoplasms respond to ICI, and definitive course of treatments are still an exception. However, robust and durable objective responses entailing the complete disappearance of neoplastic lesions and no relapse are not considered impossible anymore.
Multiple anti-PD1 and anti-PD-L1 monoclonal antibodies (mAbs) are under evaluation in digestive cancers (Eléonore de Guillebon et al., WJGO 2016). Nonetheless, there are a few cancer types that appear to be rather refractory to ICI, and that is most of the case of colorectal cancer (CRC). The fact that CRC does not respond to

ICI appears somehow paradoxical, since the first sophisticated analyses of the immunological tumor microenvironment have been performed on CRC specimens, yielding the conclusion that the “immune contexture” has a critical impact on the outcome of the patients (Galon et al., Science 2006).
Approximately 15% of the CRC are deficient for the DNA mismatch repair (dMMR) system inducing a state of genetic instability, also called MSI-H (high microsatellite unstability) CRC. MMR gene (*MLH1*, *MSH2*, *MSH6*, *PMS2*) inactivation is due to either a constitutional mutation in Lynch syndrome or a somatic inactivation in sporadic case (mostly *MLH1* hypermethylation). This deficiency is responsible for a high mutational load (frameshift mutations due to inactivation of MMR system) in MSI-H tumors and the generation of several neo-antigens, which drives a high anti-tumor inmmune response and an abundant peri- and intra-tumor infiltrating lymphocyte (TIL) (Tougeron D, et al. Mod Pathol 2009). In addition, strong PD-L1 expression was found in dMMR CRC as compare to proficient MMR (pMMR) CRC (Droeser RA, et al. Eur J Cancer 2013). Localized dMMR CRC have a better prognosis than pMMR CRC, probably because of this neo-antigens associated with T CD8+ specific immune response. In the metastatic CRC (mCRC) things are a bit different as i) the frequency of dMMR is only 4-7% and ii) the good prognosis conferred by MSI+ status is more controversial (Koopman et al., Br J Cancer 2009).
Defective in mismatch repair system largely increases the incidence of somatic mutations and hence the immunogenicity of cancer cells. Preliminary results suggests that patients with dMMR CRC seem to benefit from the administration of a PD-1-targeting mAb in chemoresistant patients with multiple chemotherapy lines. (Le DT, et al. N Engl J Med 2015) These encouraging results have been recently updated on 28 patients with MSI-H mCRC and an impressive 56% response rate and 89% disease control rate were reported as patients were all pretreated (Le DT et al., ASCO 2016, CSS103). Recently again another anti-PD1 molecule alone or in combination with an anti-CTLA4 mAb was tested in MSI-H mCRC and showed interesting results in heavily pretreated patients with a 56% and 81% disease control rate for the mono and combo therapies respectively. (Overmann et al., ASCO 2016, A3501) CTLA-4 (Cytotoxic T lymphocyte associated antigen 4) is another immune checkpoint expressed on T cells. CTLA-4 transmits an inhibitory signal to T cells to prevent early excessive T cell activation.
Anti-PD1 are now registered for patients with metastatic melanomas and lung carcinomas (Hamid N et al., Engl J Med 2013 ; Topalian et al., N Engl J Med 2012), which are known to have a high level of mutations (Vogelstein B et al., Science 2013). As melanomas and lung carcinomas, dMMR mCRC have high mutation load and abundant TIL within the tumor and these two conditions may be a prerequisite for ICI efficacy. Since ICIs seem as promising in dMMR CRC as in other tumors, the same major challenges will be faced. Expression of PD-1 or PDL1 have also been investigated to predict the efficacy of these new anti-cancer agents, but pathological quantification of these molecules still remains controversial, remembering of the difficult and time consuming work that was necessary to standardize HER2 expression/amplification in breast and gastric cancers. In fact PD-L1 expression seems to correlate with clinical outcome but objective responses have been observed in PD-L1 negative tumors. Moreover, definition of a PD-L1 positive tumor needs standardization, given that the threshold of positivity varies between 1 and 5% across different studies and also given that PD-L1 expression can be analyzed either on tumor cells or on tumor-infiltrating cells (Granier C et al., J OncoPathology 2014). Predictive value of PD-L1 expression and others biomarkers remains to be evaluated in dMMR mCRC treated with ICI.
Another difficulty is evaluation of treatment response since initial progression or appearance of new lesions are not rare and can precede objective response (Topalian SL et al., N Engl J Med 2012). Immune cell infiltration can explain these features. Recently, immune-related response criteria have been defined and await prospective validation (Wolchok JD et al., Clin Cancer Res 2009).

The Avelumab anti-PD-L1 antibody has been recently tested in many different tumor types with promising results and is currently under investigation in phase III trials in gastric cancer, but no data on the efficacy of this immune checkpoint inhibitor in dMMR mCRC are currently available. In addition, only anti-PD1 mAbs used has been investigated in dMMR CRC and not anit-PD-L1 mAbs. Moreover, only results of anti-PD1 mAbs in chemoresistant mCRC (third line or more) have been reported up until now.

We thus propose here to test the efficacy and safety of Avelumab as a second line treatment in dMMR mCRC patients who have failed to a standard first line chemotherapy +/- targeted therapy.

ADMINISTRATIVE CONSIDERATIONS

TRIAL SPONSOR

The study sponsor is the Fédération Francophone de Cancérologie Digestive (FFCD). The study was registered under number EudraCT 2016-004575-49.

REMINDER OF CURRENTLY APPLIED TEXTS

This test will take place according to French law, in accordance with the ethical principles of the Helsinki Declaration of 1964 and its revisions, Good Clinical Practice of the International Conference on Harmonization (ICH-E6, 7/17/96), with the European Directive (2001/20/EC) on the conduct of clinical trials, the modified Huriet Law (12.20.98) on the Protection of Persons participating in Biomedical Research and the provisions of the National Commission on Computer Technology and Freedom [Commission Nationale Informatique et Libertés] (Act No. 94-548 of 07/01/94 supplementing law No. 78-17 dated 6/01/78).

CIVIL LIABILITY INSURANCE

An insurance policy was taken out by the Sponsor on 27/02/2017 under number 137681, in accordance with Article L 1121-10 of the Public Health Code (Appendix 9).

REQUEST FOR CPP AND ANSM AUTHORIZATION

This protocol received the approval of the CPP *[Comité de Protection des Personnes]* (Protection of persons committee) on 27/04/2017 (Appendix 12).
This protocol received a favorable decision from the ANSM *[Agence Nationale de Sécurité du médicament et des produits de santé]* (French national agency for medicines and health products safety) on 11/07/2017 (Appendix 13).

OBTAINING THE PATIENT'S CONSENT

The investigator undertakes to collect the patient's informed clinical and biological consent in writing (information sheets and informed consent forms in Appendices 1 and 2) before including the patient in the study. A copy of these consent forms must be kept by the investigator for 15 years, to be presented to the governing authorities in the event of an inspection. The original must be given to the patient.

In accordance with the recommendations of the Cancer Plan (Measure 5.1.), this document was submitted to the Committee of Patients for Clinical Research (CPRC) *[Comité de Patients pour la Recherche Clinique]* of the National League Against Cancer *[Ligue Nationale Contre le Cancer]*.

INFORMING HOSPITAL MANAGEMENTS AND RESEARCH AGREEMENT

Before instigating the study, hospital managements will be informed by the Sponsor of the investigator's interest in taking part in this trial.
A research contract without additional cost will be drawn up between the administrator of the investigation center and the sponsor.

DATA ARCHIVING

The files will remain confidential and can only be consulted under the responsibility of the doctors in charge of the patients. In the event of inspection, the Sponsor and the health authorities will have direct access to these documents.
At the end of the trial, the study documents will be kept by the investigator for 15 years.

COMPUTER SUPPORT

In accordance with the text of law n° 78-17 of 6 January 1978 modified by the law of 9 August 2004 concerning computers, files and freedom, the trial data will be censured in a computer databank at the FFCD RMAC (Randomization-Management-Analysis Center), excluding patient identity information.

PROCESSING OF THE DATA

The FFCD RMAC will be responsible for data management and analysis.

MONITORING, QUALITY ASSURANCE AND INSPECTIONS BY THE AUTHORITIES

The investigator agrees in advance that the files of patients included can be consulted by a person authorised by the FFCD and/or the health authorities, in order to proceed with an audit. The on site file visits which will be planned in agreement with the investigator may be performed during or after the trial inclusion period.
This protocol will be monitored by the FFDC mobile CRAs.

RULES OF PUBLICATION

They will comply with those drawn up by the PRODIGE group (Appendix 10).

APPENDICES

APPENDIX 1: CLINCIAL INFORMED CONSENT

APPENDIX 2: BIOLOGICAL INFORMED CONSENT

APPENDIX 3: BIOLOGICAL STUDY

Ancillary study on blood and tumor samples (primary and/or metastasic) to investigate predictive and prognostic factors for response to treatment.
As a minimum, this study includes the determination of an immunoscore at tumor tissue level, as well as tumor DNA and RNA analyses and ctDNA analyses (baseline and variation under therapy).
The main objective is to generate hypotheses for future biomarkers predictive of response to immune checkpoint inhibitors, notably based on ctDNA (at baseline and ctDNA decrease under treatment) but also on tumor IHC (PD-L1, PD-1, PD-L2, CD8, CD4, CD3, FoxP3), analysis of mutational load and hypermethylated status. in addition to somatic mutations and immunoscore
.
Microbiota ancillary study (stool sampling) with the aim of studying the relationship between the composition of the intestinal microbiota (before and during treatment) and the antitumor response to avelumab or chemotherapy. The results of this study could open up new prospects for the manipulation of intestinal microbiota (e.g. fecal transplantation or microbiota supplementation), with the potential to improve the identification of responders to immune checkpoint inhibitors, and to enhance their efficacy and tolerability.

APPENDIX 4: WHO PERFORMANCE INDEX – CLEARANCE CALCULATION

GENERAL CONDITION – WHO SCALE

0 = able to carry on all pre-disease activities without restriction.

1 = restricted in physically strenuous activity, but ambulatory and able to carry out light work.

2 = ambulatory and capable of self-care but unable to carry out any work activities. In bed less than 50 % of the time.

3 = capable of just a few personal care activities. Bed-ridden or in a wheelchair more than 50% of the time.

4 = incapable of taking care of him/herself, permanently bed-ridden or in a wheelchair.

CLEARANCE:

MDRD (*Modification of the Diet in Renal Disease*) formula (Levey, 2000):
186.3 × (creatinine (in mmol/L)/88.4) × 1154 age-0203 (x 0.742 if female x 1.21 if black skin)

APPENDIX 5: RECIST CRITERIA VERSION 1.1 AND IMMUNE RELATED RECIST

"New response evaluation criteria in solid tumors: Revised RECIST guideline (version 1.1)" E.A. Eisenhauer, P. Therasse, J. Bogaerts, L.H. Schwartz, D. Sargent, R. Ford, J. Dancey, S. Arbuck, S. Gwyther, M. Mooney, L. Rubinstein, L. Shankar, L. Dodd, R. Kaplan, D. Lacombe, J. Verweij ;
Eur J Cancer, 4 5 (2 0 0 9) 2 2 8 –2 4 7.

Lesions on inclusion:
Lesions and lymph nodes are classified separately as being measurable or non-measurable.

Measurable disease
For a lesion to be judged measurable, at least one of its dimensions must be accurately measurable (the longest dimension, in the measurement table, must be reported).
To be measurable, the lesions must have a minimum measurement of
≥ 10 mm on scanning (with a maximum CT bandwidth of 5 mm)
≥ 10 mm on clinical examination (measurable with callipers) (lesions which cannot be measured precisely must be listed as non-measurable)
20 mm on a chest X-ray
For a malignant lymph node to be considered pathological and measurable, it must have a smallest axis of ≥ 15 mm (the smallest axis being the one perpendicular to the largest dimension of the node). Only the length of this smallest axis will be reported, both on entry and during follow-up.

Non-measurable disease
All other lesions, including small lesions (greatest diameter < 10 mm on a scan or lymph nodes with the smallest axis ≥ 10 mm and < 15 mm) as well as lesions not actually measurable: leptomeningeal disease, ascites, pleurisy, pericarditis, inflammatory breast disease, pulmonary or cutaneous carcinomatous lymphangitis, abdominopelvic masses detected by clinical examination but not confirmed by imaging, and cystic lesions.
NB: bone lesions, simple cystic lesions and lesions previously treated locally require particular consideration (see comments below).

Target lesions
Target lesions are selected from the measurable lesions presented by the patients on entry to the study. **A maximum of 5 target lesions are selected in all, with a maximum of 2 target lesions per organ.** Target lesions are selected to represent all the invaded organs, by choosing the biggest lesions (in their greatest dimension) which can also be monitored throughout the trial using the method used at the initial examination. Lymph nodes can be considered to be target lesions if their smallest axis (measured by CT-scan) is ≥ 15 mm.

The sum of the diameters of these target lesions (longest axis for lesions and smallest for lymph nodes) will be monitored throughout the trial to evaluate response or progression.

Non-target lesions
All other lesions are identified as non-target lesions and are also noted at inclusion. They are not measured but are monitored throughout the trial.

Treatment response criteria:
Target lesions:

Complete response (CR): Disappearance of all the lesions. Furthermore, all the lymph nodes (target or non-target) must have reached < 10 mm along their *smallest* axis.

Note: lymph nodes selected as target lesions must always be measured (smallest anatomical axis used for the BASELINE examination), even if they shrink in size during the study and their smallest axis becomes < 10 mm. From then on, when the lymph nodes are used as target lesions, the "sum" of their dimensions is not necessarily zero, even with a complete response, because a normal lymph node is defined as having a smallest axis of < 10 mm. To obtain a complete response each node must have reached a dimension of < 10 mm along its smallest axis.

Partial response (PR): At least 30% reduction in the sum of the diameters of target lesions relative to the initial sum of diameters (BASELINE examination).

Progression (PD): ≥ 20% increase in the sum of the diameters of target lesions relative to the smallest sum of diameters observed during the study (NADIR), including the baseline visit. As well as this relative 20% increase, this sum must increase by at least 0.5 cm.
NB: the appearance of one or more new lesions is also considered progression.
Note: if there is progression relative to the NADIR and a response relative to the BASELINE examination, then progression takes precedence.

Stabilization (SD): Neither PR (or CR) nor PD.

Non-target lesions

Complete response: Disappearance of all the non-target lesions and normalization of tumoral markers. All lymph nodes must have reached a small diameter of < 10 mm.
Incomplete response - Stabilization: Persistence of at least one non-target lesion and/or tumoral marker above normal.
Progression: **Definite** increase in size of non-target lesions or development of a new lesion.

Overall response:

Target lesions	Non-target lesions	New lesion		Overall response
CR	CR	No	=	**CR**
CR	No CR/No PD	No	=	**PR**
CR	Not evaluated	No	=	**PR**
PR	No PD or not all evaluated	No	=	**PR**
SD	No PD or not all evaluated	No	=	**SD**
Not all evaluated	No PD	No	=	**Cannot be evaluated**
PD	Indifferent	Yes or no	=	**PD**
Indifferent	PD	Yes or no	=	**PD**
Indifferent	Indifferent	Yes	=	**PD**

Comments on the measurability of lesions on entry
Bone lesions:
Imaging by bone scintigraphy, PET scan and "plain films" are not considered adequate for measuring bone lesions. However, these techniques can be used to confirm the presence or disappearance of bone lesions.
Lytic or mixed lytic-osteoblastic bone lesions, which contain an identifiable soft tissue component, can be considered to be measurable lesions as long as they can be measured using cross-sectional imaging techniques such as CT or MRI, and the soft tissue component fulfills the conditions for measurability given above.
Cystic lesions:
Lesions corresponding to the diagnosis of simple cyst by X-ray are not considered to be malignant lesions (neither measurable nor non-measurable)
Malignant cystic lesion can be included as measurable lesions as long as they meet the measurability criteria defined above. However, if the patient has other non-cystic lesions, these will be given precedence when choosing target lesions.
Lesions previously treated locally:
Lesions located in a region which has been previously irradiated or treated with another locoregional treatment are not usually considered to be measurable, except for lesions which have progressed since the local treatment. The study protocol must detail the specific conditions to be met in order to consider such lesions as being measurable.

IRECIST criteria of the immune response

APPENDIX 6: EVALUATION OF TOXICITY (NCI CTC V4.0)

EVALUATION OF TOXICITY NCI-CTC V4.0

http://evs.nci.nih.gov/ftp1/CTCAE/About.html

then click on "Files: Data on "CTCAE 4.03 2010-06-14.xls"

APPENDIX 7: SUMMARY PRODUCT CHARACTERISTICS OF THE STUDY (OXALIPLATIN, IRINOTECAN, 5FU, AF, TARGETED TREATMENTS)

PRODUCT FEATURE SUMMARY - FLUOROURACIL TEVA 1000 mg/20mL®

SUMMARY OF PRODUCT CHARACTERISTICS - ELVORINE®

SUMMARY OF PRODUCT CHARACTERISTICS – ELOXATINE®

SUMMARY OF PRODUCT CHARACTERISTICS – CAMPTO®

SUMMARY OF PRODUCT CHARACTERISTICS – VECTIBIX®

SUMMARY OF PRODUCT CHARACTERISTICS - AVASTIN®

SUMMARY OF PRODUCT CHARACTERISTICS - ERBITUX®

SUMMARY OF PRODUCT CHARACTERISTICS – ZALTRAP®

In order to obtain the SmPCs mentioned above please click on the following link:

http://base-donnees-publique.medicaments.gouv.fr/

Information on AVELUMAB will be provided in the AVELUMB Investigator Brochure

APPENDIX 8: QUALITY OF LIFE QLQ-C30

PRODIGE 54 – SAMCO: EORTC QLQ-C30 (version 3.0)

We are interested in some things about you and your health. Please answer all of the questions yourself by **circling the number** that best applies to you. There are no "right" or "wrong" answers. The information that you provide will remain strictly confidential.

Please specify:

The first three letters of your surname: ↦↦↦

The first two letters of your first name: ↦↦

Date of birth: ↕O↕O↕⇕⇕O

Today's date: ↕O↕O↕⇕⇕O

	Not at all	A little	Quite a bit	Very much
1. Do you have any trouble performing strenuous activities, like carrying a heavy shopping bag or a suitcase?	1	2	3	4
2. Do you have any trouble taking a long walk?	1	2	3	4
3. Do you have any trouble taking a short walk outside of the house?	1	2	3	4
4. Do you need to stay in bed or a chair during the day?	1	2	3	4
5. Do you need help with eating, dressing, washing yourself or using the toilet?	1	2	3	4

During the past week:

	Not at all	A little	Quite a bit	Very much
6. Have you been limited in doing either your work or other daily activities?	1	2	3	4
7. Have you been limited in pursuing your hobbies or other leisure time activities?	1	2	3	4
8. Have you been short of breath?	1	2	3	4
9. Have you been in pain?	1	2	3	4
10. Have you had need to rest?	1	2	3	4
11. Have you had trouble sleeping?	1	2	3	4
12. Have you felt weak?	1	2	3	4
13. Have you had a loss of appetite?	1	2	3	4
14. Have you felt nauseated (sick)?	1	2	3	4
15. Have you vomited?	1	2	3	4
16. Have you been constipated?	1	2	3	4
17. Have you had diarrhea?	1	2	3	4
18. Have you been tired?	1	2	3	4
19. Has pain interfered with your daily activities?	1	2	3	4
20. Have you had difficulty in concentrating on things, like reading a newspaper or watching television?	1	2	3	4
21. Have you felt tense?	1	2	3	4
22. Have you felt worried?	1	2	3	4
23. Have you felt irritable?	1	2	3	4
24. Have you felt depressed?	1	2	3	4
25. Have you had difficulty remembering things?	1	2	3	4
26. Has your physical condition or medical treatment interfered with your family life?	1	2	3	4
27. Has your physical condition or medical treatment interfered with your social activities (for example going out with friends, going to the cinema etc.)?	1	2	3	4
28. Has your physical condition or medical treatment caused you financial difficulties?	1	2	3	4

For the following questions please circle the number between 1 and 7 that best applies to you

29. How would you rate your overall health during the past week?

1	2	3	4	5	6	7
Very poor						Excellent

30. How would you rate your overall quality of life during the past week?

1	2	3	4	5	6	7
Very poor						Excellent

© Copyright 1999 EORTC Quality of Life Study Group, version 3.0 All rights reserved. All rights reserved. English version

APPENDIX 9: SERIOUS ADVERSE EVENT DECLARATION FORM

**PRODIGE 54 – FFCD 1603 – SAMCO** T ☐ M ☐<br>**SERIOUS ADVERSE EVENT REPORT FORM (SAE)** CRA Initials ↕⇕○	**Page 1/3**

SPONSOR : FFCD **PRINCIPAL INVESTIGATOR : Pr Julien TAIEB**

Study title : MULTICENTER RANDOMIZED PHASE II STUDY COMPARING THE EFFECTIVENESS AND TOLERANCE OF AVELUMAB VERSUS STANDARD 2nd LINE TREATMENT CHEMOTHERAPY IN PATIENTS WITH COLORECTAL METASTATIC CANCER WITH MICROSATELLITE INSTABILITY (MSI) **N° EudraCT** : 2016-004575-49

Author of the declaration : Dr ☐ – Pr ☐ - CRA ☐ - Other ☐, specify : ……………………………….

Name : .... Center : ....

Phone : .... Fax :..

SAE n° : ↕○ **Type of report :** ☐ **initial** ☐ **follow-up n° :** ↕○

Date of report : ↕○↕○⇕⇕⇕○

SPACE RESERVED FOR DATA CENTER (CRGA)

Date of reception : ↕○↕○⇕⇕⇕○ **Sponsor reference for the event :**...............................

Patient N° ↕⇕○ **Patient's initials :** ↕○ ↦ **Sex :** ☐ Female ☐ Male

Date of birth : ↕○↕○⇕⇕⇕○ **Inclusion date :** ↕○↕○⇕⇕⇕○

☐ **Arm A – Chemotherapy +/- targeted therapy**

☐ **Arm B - Avelumab**

Weight (kg) : ↦↦↦ **Height (cm) :** ↦↦↦

**Serious adverse event :**	**Date of start :** ↕○↕○⇕⇕⇕○<br>**Date of end :** ↕○↕○⇕⇕⇕○

**Seriousness criteria**	**Grade/severity**	**Outcome**
☐ hospitalization (or prolongation)<br>☐ medically significant<br>☐ durable or significant disability or incapacity<br>☐ life-threatening<br>☐ death<br>☐ congenital anomaly or fetal malformation	↦<br>**Coded as NCI-CTC 4.0**<br>**If not applicable, specify :**<br>1 = mild<br>2 = moderate<br>3 = severe<br>4 = life-threatening<br>5 = death due to SAE	☐ recovered/resolved without sequelae<br>☐ recovered/resolved with sequelae<br>☐ recovering/resolving<br>☐ not recovered/resolved<br>☐ death

If hospitalization Date of admission : ≐λ≐λ≐∐∐λ ongoing ☐ Date of discharge : ≐λ≐λ≐∐∐λ

If death Date of death : ≐λ≐λ≐∐∐λ Death cause ________________________________

Specify : ☐ Death related to SAE ☐ Death for which SAE may have contributed ☐ Death not related to SAE

Description
Please describe below the chronological sequence of events including the history of the disease and the relevant concomitant diseases existing in the context of the Serious Adverse Event.

**PRODIGE 54 – FFCD 1603 – SAMCO** T ☐ M ☐<br>**SERIOUS ADVERSE EVENT REPORT FORM (SAE) CRA Initials ↕⇕○**	**Page 2/3** **SAE n°: ↕○**<br>initial ☐ follow-up ☐<br>**Patient N° : ↕⇕○**

If arm A, specify which chemotherapy is used : **FOLFOX** ☐ **FOLFIRI** ☐
Administration of targeted therapy (*): No targeted therapy ☐ Aflibercept ☐ Bevacizumab ☐ Cetuximab ☐ Panitumumab ☐

Drug	Administration	Last dose	SERIOUS ADVERSE EVENT REPORT FORM (SAE)
**Avelumab**<br>☐ Not applicable	Date of first administration :<br>↕○↕○⇕⇕○<br>Date of last administration before SAE :<br>↕○↕○⇕⇕○<br>Cycle n°: ↦↦	________ mg	☐ Dose not changed<br>☐ Dose reduced, specify : new dose : _________ mg<br>☐ Temporary withdrawal, specify date of reintroduction : ↕○↕○⇕⇕○<br>☐ Definitive withdrawal, specify date : ↕○↕○⇕⇕○
**Oxaliplatin**<br>☐ Not applicable	Date of first administration :<br>↕○↕○⇕⇕○<br>Date of last administration before SAE :<br>↕○↕○⇕⇕○<br>Cycle n°: ↦↦	________<br>mg	☐ Dose not changed<br>☐ Dose reduced, specify : new dose : _________ mg<br>☐ Temporary withdrawal, specify date of reintroduction : ↕○↕○⇕⇕○<br>☐ Definitive withdrawal, specify date : ↕○↕○⇕⇕○
**Irinotecan**<br>☐ Not applicable	Date of first administration :<br>↕○↕○⇕⇕○<br>Date of last administration before SAE :<br>↕○↕○⇕⇕○<br>Cycle n°: ↦↦	________<br>mg	☐ Dose not changed<br>☐ Dose reduced, specify : new dose : _________ mg<br>☐ Temporary withdrawal, specify date of reintroduction : ↕○↕○⇕⇕○<br>☐ Definitive withdrawal, specify date : ↕○↕○⇕⇕○
**Folinic acid**<br>☐ Not applicable	Date of first administration :<br>↕○↕○⇕⇕○<br>Date of last administration before SAE :<br>↕○↕○⇕⇕○<br>Cycle n°: ↦↦	D-L ☐ L ☐<br>________<br>mg	☐ Dose not changed<br>☐ Dose reduced, specify : new dose : _________ mg<br>☐ Temporary withdrawal, specify date of reintroduction : ↕○↕○⇕⇕○<br>☐ Definitive withdrawal, specify date : ↕○↕○⇕⇕○
**5-FU bolus**<br>☐ Not applicable	Date of first administration :<br>↕○↕○⇕⇕○<br>Date of last administration before SAE :<br>↕○↕○⇕⇕○<br>Cycle n°: ↦↦	________<br>mg	☐ Dose not changed<br>☐ Dose reduced, specify : new dose : _________ mg<br>☐ Temporary withdrawal, specify date of reintroduction : ↕○↕○⇕⇕○<br>☐ Definitive withdrawal, specify date : ↕○↕○⇕⇕○
**5-FU infusion**<br>☐ Not applicable	Date of first administration :<br>↕○↕○⇕⇕○<br>Date of last administration before SAE :<br>↕○↕○⇕⇕○<br>Cycle n°: ↦↦	________<br>mg	☐ Dose not changed<br>☐ Dose reduced, specify : new dose : _________ mg<br>☐ Temporary withdrawal, specify date of reintroduction : ↕○↕○⇕⇕○<br>☐ Definitive withdrawal, specify date : ↕○↕○⇕⇕○

**Targeted therapy (*)**<br>☐ Not applicable	Date of first administration :<br>↕○↕○⇕⇕○<br>Date of last administration before SAE :<br>↕○↕○⇕⇕○<br>Cycle n°: ↦↦	_________ mg	☐ Dose not changed<br>☐ Dose reduced, specify : new dose : _________ mg<br>☐ Temporary withdrawal, specify date of reintroduction : ↕○↕○⇕⇕○<br>☐ Definitive withdrawal, specify date : ↕○↕○⇕⇕○

Disparition of event after stop or dose reduced of suspected drugs :
Yes ☐ No ☐ Unknown ☐ Not applicable ☐

Recurrence of event after reintroduction of suspected drugs :
Yes ☐ No ☐ Unknown ☐ Not applicable ☐

Concomitants drugs : (regular treatment of the patient or other drugs received within 15 days)

**Drugs**	**Date of start**	**Ongoing**	**Date of end**	**Dose**	**Indication**
	↕○↕○⇕⇕○	☐	↕○↕○⇕⇕○		
	↕○↕○⇕⇕○	☐	↕○↕○⇕⇕○		
	↕○↕○⇕⇕○	☐	↕○↕○⇕⇕○		
	↕○↕○⇕⇕○	☐	↕○↕○⇕⇕○		
	↕○↕○⇕⇕○	☐	↕○↕○⇕⇕○		
	↕○↕○⇕⇕○	☐	↕○↕○⇕⇕○		
	↕○↕○⇕⇕○	☐	↕○↕○⇕⇕○		
	↕○↕○⇕⇕○	☐	↕○↕○⇕⇕○		
	↕○↕○⇕⇕○	☐	↕○↕○⇕⇕○		
	↕○↕○⇕⇕○	☐	↕○↕○⇕⇕○		

Causality assesment

**Avelumab :**	☐ **related**	☐ **not related**	☐ **doubtfully related** or :	☐ **not applicable**
**Oxaliplatine :**	☐ **related**	☐ **not related**	☐ **doubtfully related** or :	☐ **not applicable**
**Irinotecan :**	☐ **related**	☐ **not related**	☐ **doubtfully related** or :	☐ **not applicable**
**Folinic acid :**	☐ **related**	☐ **not related**	☐ **doubtfully related** or :	☐ **not applicable**
**5-FU bolus :**	☐ **related**	☐ **not related**	☐ **doubtfully related** or :	☐ **not applicable**
**5-FU infusion :**	☐ **related**	☐ **not related**	☐ **doubtfully related** or :	☐ **not applicable**
**Targeted therapy :**	☐ **related**	☐ **not related**	☐ **doubtfully related** or :	☐ **not applicable**

If the causality assessment between SAE and study drugs are « not related », which is, to your opinion, the cause of SAE ? (tick the appropriate box(es))

☐ Progression of metastatic colorectal cancer

☐ Preexisting condition, specify : ______________________________

☐ Concomitant drug, specify which one : ______________________________

☐ Other illness, specify : ____________________________

☐ Other, specify : ___________________________________

PLEASE ATTACH ANONYMIZED HOSPITALIZATION REPORT, AND, IF NEEDED BIOLOGICAL TESTS, COMPLEMENTARY EXAMS…

Form to fax at Data Center CRGA Dijon Fax : 03 80 38 18 41

DATE : **NAME :** **SIGNATURE :**

APPENDIX 10: RULES OF PUBLICATION OF PRODIGGE TRIALS

PRODIGE RULES OF PUBLICATION

The publication rules used for this study will be those in application at the time of the last inclusion.

(Partnership Version 3, May 2012)

PRODIGE RULES OF PUBLICATION

The publication of PRODIGE trials by a high-quality journal is an essential objective for therapeutic progress. This publication takes place under the responsibility of the PRODIGE Coordination Committee (CPP) which decides:

- on the time of publication of the preliminary results and the final results of a study.
  All information from testing is confidential, at least until suitable analysis and control by the Sponsor, the coordinating investigator and the trial statistician are complete.
- On the composition of an Editorial Committee (usually 7 members at most).

The CPP can delegate these jobs to the trial coordinator.

The CPP validates the choices made and ensures that the deadlines are met. The absence of the CPP response within one month of submission to the Editorial Committee is taken as acceptance.

1. The Editorial Committee consists of:

- The steering committee as defined in the internal regulation.
- The most important contributors

For cooperative, national or international trials, if other associations have provided at least 10% of the total number, the Editorial Committee will include a representative, designated among the investigators, from each of the other associations.

A coordinator (for a country or association) who will not have included any patients will not be on the Editorial Committee, nor will an author of the publication, but they will be thanked at the end of the article.

2. The principal author agrees to submit for publication within a deadline determined by the CPP. This deadline should not exceed one year after the trial is closed. If this author cannot meet the deadline, the CPP will appoint a new author who will become the principal author. To help write the articles based on trials, a medical author may be used and writing workshops organised for the main author, in association with the statistician.

3. Before each publication, the project manager of the study sends the CCP the list of authors accompanied by the table of inclusions by investigation center.
The CPP validates the number and order of authors before each release, in compliance with PRODIGE publishing rules.
Remotely from the CCP meetings, validation will be done by e-mail within 7 days, where absence of response is deemed to be endorsement.

4. **Title of publication or oral communications:** the name of the trial must be PRODIGE XX, possibly followed by the name given by the Sponsor group.

5. **The publication authors** are ordered according to the work done and the number of patients included:

- The principal editor
- A limited number of investigators (1 per center) in their order of participation, and generally just one per center, but for some centers the Editorial Committee may decide to include 2 investigators. This rule can be weighted to allow certain small and medium-sized centers which worked hard to include as many patients as possible, to be included as authors. The CPP will validate this weighting so that nobody is left out.
- The last author is usually the coordinator of the trial or even the principal author, or a person who has had a decisive influence in the design and/or conduct of the trial (this can be the co- coordinator). In case of discussion, the CPP will decide.
- The maximum number of authors allowed by the journals will be used.
- No matter how many patients were included, there will be at least one author representing one of the 2 partners (FFCD or UNICANCER GI)

- For a derivative publication or accessory work, the authors may be different from those of the first article and reflect the speciality covered by the article; e.g.: In RCT trials, an article dedicated to radiotherapy can be signed by radiotherapists who are co-investigators at centers which included patients. The last author of this derivative publication is thus the first signatory of the first article.
- The Prodige partnership is mentioned in the title or after the authors. In cooperative trials, the first association mentioned is the one which initiated the trial and the others are mentioned on condition that they included at least 5% of patients, in the order of their participation.
- For trials promoted or managed by the FFCD, a member of Inserm unit U 866 will be the second to last author and indicated as having "equally contributed", if he/she is not the principal author, to ensure that the Inserm work is taken into account.
- For trials promoted or managed by UNICANCER, a representative of the sponsor will be included among the authors.
- The statistician will be one of the authors, usually after the 3rd place. He/she can be the 1st or 2nd author of a derived publication.

All participants not included as authors will be cited at the end of the article. The managers of the study (project manager, data manager) are also cited.
He/she can be one of the authors if the CPP feels this is justified.

The partners are acknowledged. The patients and their families are acknowledged.

The authors and the Sponsor receive a copy to critique before it is sent to a journal. They agree to reply within 15 working days so that their opinion can be taken into account (30 days during the summer recess).

6. **Oral presentation** of the trial results:

With CPP and Management Committee approval an investigator can present all or part of the results orally, in his/her own name. The authors are generally the same as for the written article, but the order of authors for articles and oral presentations may vary, and also vary according to the conferences where the presentation takes place. In some cases (multidisciplinary studies or pathological, biological, echoendoscopic studies or imaging at the same time as a therapeutic trial, for example) other authors may be chosen according to their work. The name of the study will remain PRODIGE XX (cf. § 3) and the other associations, if any, will be cited.

7. **These rules must be included in the appendices of the PRODIGE trial protocols**

11: INSURANCE ATTESTATION

sham
PARTAGEONS PLUS QUE L'ASSURANCE

ATTESTATION D'ASSURANCE

RESPONSABILITÉ CIVILE
PROMOTEUR DE RECHERCHES INTERVENTIONNELLES relevant de l'article L 1121-1, 1° du Code de la santé publique

Loi n°2012-300 du 5 mars 2012 et textes d'application subséquent

SOCIÉTÉ HOSPITALIÈRE D'ASSURANCES MUTUELLES
18, rue Edouard Rochet - 69372 LYON CEDEX 08

Atteste que la **FEDERATION FRANCAISE DE CANCEROLOGIE DIGESTIVE**
FACULTE DE MEDECINE
BP 87900
21079 DIJON

A souscrit sous le n° **137681** un contrat d'assurance de la Responsabilité Civile Promoteur d'une Recherche interventionnelle relevant de l'article L 1121-1, 1° (Recherche interventionnelle comportant une intervention sur la personne non justifiée par sa prise en charge habituelle) du Code de la santé publique-conforme aux dispositions de l'article R 1121-4 du même code, afin de couvrir les obligations mises à leur charge en application de l'article L.1121-10 du même Code.

Le contrat couvre la recherche intitulée :

« PRODIGE xx (FFCD 1603) – SAMCO
Etude de phase II multicentrique randomisée comparant l'efficacité et la tolérance de l'AVELUMAB versus un traitement standard en 2ème ligne chez les patients avec un cancer colorectal métastatique avec instabilité microsatellitaire (MSI) » (Pr Julien TAIEB)

Dates prévisionnelles de début et de fin de la recherche : **Q1 2017 – Q2 2024**

Nombre prévisionnel de personnes qu'il est prévu d'inclure : **118**

La garantie s'exerce pour les recherches réalisées exclusivement en France métropolitaine et dans les départements et territoires d'Outre-mer.

La présente attestation ne constitue toutefois qu'une présomption d'assurance à la charge de la Société avant validation par les autorités compétentes.

Fait et Certifié, à LYON, 27/02/2017

Philippe BIDARD P/C Quentin GILLY
Souscription et vie des contrats
Direction établissements privés et professionnels de santé

SHAM - Société Hospitalière d'Assurances Mutuelles
18 rue Édouard Rochet - 69372 LYON Cedex 08
Tél : +33 (0)4 72 75 50 25 - Fax : +33 (0)4 72 74 22 32 - www.sham.fr

APPENDIX 12: FAVORABLE DECISION BY THE CPP

COMITE DE PROTECTION DES PERSONNES
SUD MEDITERRANEE III
Président: T. LAVABRE-BERTRAND. Vice-Président: J.P. BROUILLET

Référence CPP à rappeler:	2017.04.03 bis	Nîmes, le:	27 avril 2017
Lors de sa séance du:	05 avril 2017	Présidée par Mme ou M:	T. LAVABRE-BERTRAND

En présence des membres suivants: Mmes et MM:			Membres titulaires		Membres suppléants
1er Collège	Personnes qualifiées en recherche biomédicale	X	J.P. BROUILLET<br>S. DROUPY<br>D. MOTTET		J-Y. LEFRANT<br>R. DE TAYRAC<br>B. KEZACHIAN
	Compétents en biostatistique/épidémiologie		N. MOLINARI	X	C. DEMATTEI
	Médecins généralistes		M. GARCIA	X	P. SERAYET
	Pharmaciens hospitaliers		A. MOURGUES	X	G. LEGUELINEL
	Infirmiers	X	G. BAVILLE		F. BUHLER
2e Collège	Compétents en questions éthiques	X	T. LAVABRE-BERTRAND		P. BOURQUARD
	Psychologues	X	A. MAIZIERE-PROUST		C. AYELA
	Travailleurs sociaux		N		N
	Compétents en matière juridique	X	E. TOULOUSE-MULLER<br>N	X	C. ROLLAND<br>M. GRIT
	Représentants d'associations agréées de malades et usagers du système de santé	X<br>X	A-M. JOUBERT<br>P. BALMELLE		N<br>N
Personnes cooptées	Pédiatre		N		N
	Spécialiste pour défaut de consentement		N		N

Les membres suivants s'étant retirés: Mmes et MM:	

Le comité de protection des personnes Sud Méditerranée III a examiné les informations relatives à un projet référencé localement sous le numéro ci-dessus, et identifié par le numéro ci-dessous, relatif à:	X	Recherche interventionnelle de type 1 et 2
		Recherche non interventionnelle de type 3
		Utilisation d'éléments et produits du corps humain
		Collection d'échantillons biologiques

Numéro d'enregistrement:	EudraCT	2016-004575-49	ANSM	

Intitulé du projet:	"Prodige xx-ffcd 1603-samco étude de phase II multicentrique randomisée comparant l'efficacité et la tolérance de l'avelumab versus un traitement standard en 2eme ligne chez les patients avec cancer colorectal métastatique et instabilité microsatellitaire (MSI)"
Promoteur	Fédération Francophone de Cancérologie Digestive
Investigateur principal ou coordonnateur:	PR TAIEB
Lieu de recherche (si soumis à autorisation):	

Au titre d'une demande d'avis concernant:	X	Projet initial		Dans le cadre de:		Première soumission
		Modification substantielle N°			X	Nouvelle soumission d'un projet modifié en réponse aux observations du comité

Date de réception du projet visé	17 mars 2017

	Le comité, ayant examiné ou réexaminé le projet soumis, exprime en séance plénière l'avis ci-contre:	X	Favorable
			Défavorable
X	Le projet ayant fait l'objet de réserves mineures lors de la délibération initiale, et celles-ci ayant été prises en compte, le comité exprime ce jour l'avis ci-contre:		Différé<br>P2P (sans 2ème passage)<br>2P (2ème passage)

Date de prise d'effet du présent avis:	27 avril 2017

Le président:	X	Le vice-président:		Le président de séance:	

Adresser la correspondance à : CPP SUD-MEDITERRANEE III, UFR MEDECINE 186, chemin du Carreau de Lanes CS 83021 30908 NIMES Cedex 2
Secrétariat : Mlle CABRERA Téléphone/Fax : 04 66 02 81 55
e-mail : cpp.sudmediterranee3@gmail.com

Page 1 sur 2

11/07/2017 16:47 +33155873492 ANSM/BIOVA PAGE 01/02

ansm
Agence nationale de sécurité du médicament et des produits de santé

AUTORISATION D'ESSAI CLINIQUE DE MEDICAMENT A USAGE HUMAIN

Nombre de pages : 1
(incluant la page de garde)

Envoi par Télécopie

Date :

**Identifiants de l'essai clinique**					
Titre	MULTICENTER RANDOMIZED PHASE II STUDY COMPARING THE EFFECTIVENESS AND TOLERANCE OF AVELUMAB VERSUS STANDARD 2nd LINE TREATMENT CHEMOTHERAPY IN PATIENTS WITH COLORECTAL METASTATIC CANCER WITH MICROSATELLITE INSTABILITY (MSI)				
Promoteur	Federation Francophone de Cancerologie Digestive			Réf. CPP	
Réf. Promoteur	FFCD-1603 (SAMCO)	N° EudraCT	2016-004575-49	Réf. ANSM	**170149A-12**

**Expéditeur**	**Destinataire** (demandeur : nom / société / tél.)
**ANSM / Direction des médicaments en oncologie, hématologie, immunologie et néphrologie**<br>Pôle Oncologie solide	Jérémie BEZ<br>Federation Francophone de Cancerologie Digestive<br>+330 3 80 39 34 83
Dossier suivi par : ANNICK NJONGA<br>Tél : 33 (0) 1 55 87 34 97 / 34 63 - Fax : 33 (0) 1 55 87 34 52<br>Mel : aec-essaiscliniques@ansm.sante.fr	Fax **03 80 38 18 41**

**CPP destinataire en copie**	**Sud-Méditerranée III (Nîmes)**	**Fax**	04.66.02.81.80
**INCA destinataire en copie**		**Fax**	**01 41 10 14 45**

Vu le code de la santé publique et notamment l'article L. 1123-8, et les dispositions réglementaires prises pour son application, et vu le dossier de demande d'autorisation d'essai clinique adressé à l'Agence nationale de sécurité du médicament et des produits de santé (ANSM) ;

Vu les compléments versés par le promoteur en date des 23, 29 et 30 juin 2017 et du 7 juillet 2017 et notamment le protocole de l'essai cité en objet modifié (version 1.1 datée du 7 juillet 2017), suite à la demande de l'ANSM;

L'autorisation mentionnée à l'article L. 1123-8 du code de la santé publique est accordée pour l'essai clinique cité en objet

Je vous demande de transmettre toute demande de modifications concernant ce dossier par courriel adressé à la boite : ams-essaiscliniques@ansm.sante.fr . Lors de l'envoi de ces dossiers, je vous demande de veiller à reporter dans l'objet du message la mention : **MSA/170149A-12** pour les MS soumises pour autorisation ou pour les dossiers mixtes (comportant des modifications soumises pour autorisation et d'autres pour information).

Si vous ne recevez pas toutes les pages de cette télécopie, veuillez contacter le secrétariat de la Direction Produit ONCOH/ Equipe ONCO au : 33 (0) 1 55 87 34 97.

Le Chef produits oncologie

11 JUIL. 2017

Vincent GAZIN

Confidentialité
Cette transmission est à l'attention exclusive du(des) destinataires ci-dessus mentionné(s) et peut contenir des informations privilégiées et/ou confidentielles. Si vous n'êtes pas le destinataire voulu ou une personne mandatée pour lui remettre cette transmission, vous avez reçu ce document par erreur et toute utilisation, révélation, copie ou communication de son contenu est interdite. Si vous avez reçu cette transmission par erreur, veuillez nous en informer par téléphone immédiatement et nous retourner le message original par courrier. Merci.

Confidentiality
This transmission is intended to the addressee(s) listed above only and may contain preferential or/and confidential information. If you are not the intended recipient, you are hereby notified that you have received the document by mistake and any use, disclosure, copying or communication of the content of this transmission is prohibited. If you have received this transmission by mistake, please call us immediately and return the original message by mail. Thank you.

code : Q18ADOC004 v02

143/147, bd Anatole France - F-93285 Saint-Denis cedex - tél. +33 (0)1 55 87 30 00 - www.ansm.sante.fr **Page 1 sur 1**
