# Supplementary material for: Early ctDNA and Survival in Metastatic Colorectal Cancer Treated With Immune Checkpoint Inhibitors: A Secondary Analysis of the SAMCO-PRODIGE 54 Randomized Clinical Trial
Source: JAMA Oncol. 2025 Jun 18;11(8):874–82. doi: 10.1001/jamaoncol.2025.1646 (PMC12177728; doi:10.1001/jamaoncol.2025.1646)
Supplement: Supplement 2. — eMethods. Cell free DNA extraction and analysis eFigure 1. PFS (A) and OS (B) according to ctDNA status at baseline (positive versus negative) eFigure 2. PFS (A) and OS (B) according to ctDNA concentration at baseline (above and below median; median= 1.7ng/mL) eFigure 3. PFS (A) and OS (B) according to ctDNA response eFigure 4. DNA concentrations in ng/mL at baseline (V1) and after one month of treatment (V2) according to treatment group and treatment response e Figure 5. Early ΔctDNA variations between V1 and V2, disease control according to RECIST and overall survival eTable 1. Relation of ctDNA response to standard prognostic variables for PFS in MSI mCRC patients treated with immunotherapy eTable 2. Univariable analysis of PFS based on key clinical and molecular variables [file jamaoncol-e251646-s002.pdf]

Supplemental Online Content

Taïeb J, Sullo FG, Lecanu A, et al. Early ctDNA and Survival in Metastatic Colorectal Cancer Treated With Immune Checkpoint Inhibitors: A Secondary Analysis of the SAMCO-PRODIGE 54 Randomized Clinical Trial. *JAMA Oncol.* Published online June 18, 2025. doi:10.1001/jamaoncol.2025.1646

eMethods. Cell free DNA extraction and analysis

eFigure 1. PFS (A) and OS (B) according to ctDNA status at baseline (positive versus negative)

eFigure 2. PFS (A) and OS (B) according to ctDNA concentration at baseline (above and below median; median= 1.7ng/mL)

eFigure 3. PFS (A) and OS (B) according to ctDNA response

eFigure 4. DNA concentrations in ng/mL at baseline (V1) and after one month of treatment (V2) according to treatment arm and treatment response

e Figure 5. Early ΔctDNA variations between V1 and V2, disease control according to RECIST and overall survival

eTable 1. Relation of ctDNA response to standard prognostic variables for PFS in MSI mCRC patients treated with immunotherapy

eTable 2. Univariable analysis of PFS based on key clinical and molecular variables

This supplemental material has been provided by the authors to give readers additional information about their work.

© 2025 Taïeb J et al. *JAMA Oncol*.

eMETHODS: Cell free DNA extraction and analysis

Cell-free DNA (cfDNA) was extracted using the QIAamp Circulating Nucleic Acid Kit (Qiagen, Hilden, Germany) from 5 mL of plasma according to the manufacturer's instructions and quantified using the Qubit 2.0 Fluorometer (Thermo Fisher, Waltham, MA, USA). The extracted DNA was bisulfite converted using the EZ DNA Methylation-Gold Kit (Zymo Research, Irvine, CA, USA). Following bisulfite conversion, DNA was screened for the presence of hypermethylated regions of WIF1 and NPY genes by droplet-based digital PCR (MetddPCR) as previously described[12]. A methylation-insensitive target on the albumin (ALB) gene was used as an internal control for each ddPCR reaction. MetddPCR was perform using the QX200™ system and droplet thermocycling performed using the C1000 Touch Thermal Cycler (Bio-Rad, Hercules, CA, USA). Data were analyzed using the QuantaSoft™ Analysis Pro software v1.7.4. The Limit of Blank (LOB), defined as the number of false positive events in ddPCR testing, had been previously established, and samples were classified as positive when the number of positive droplets exceeded the LOB[11,14].

© 2025 Taïeb J et al. *JAMA Oncol*.

eFigure 1: PFS (A) and OS (B) according to ctDNA status at baseline (positive versus negative)

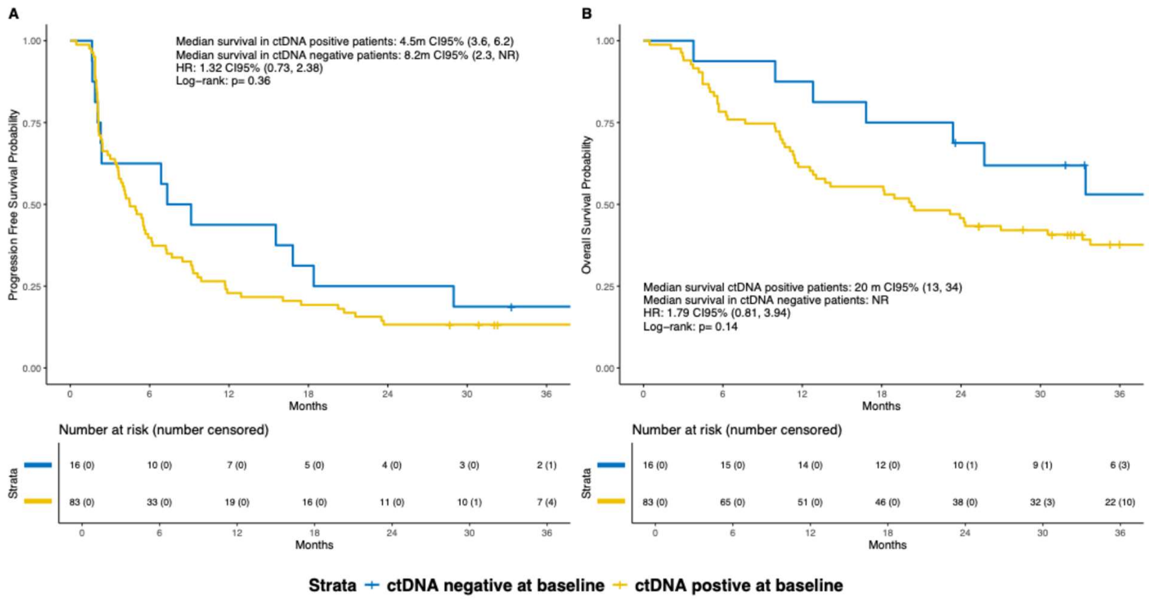

© 2025 Taïeb J et al. *JAMA Oncol*.

eFigure 2: PFS (A) and OS (B) according to ctDNA concentration at baseline (above and below median; median= 1.7ng/ml)

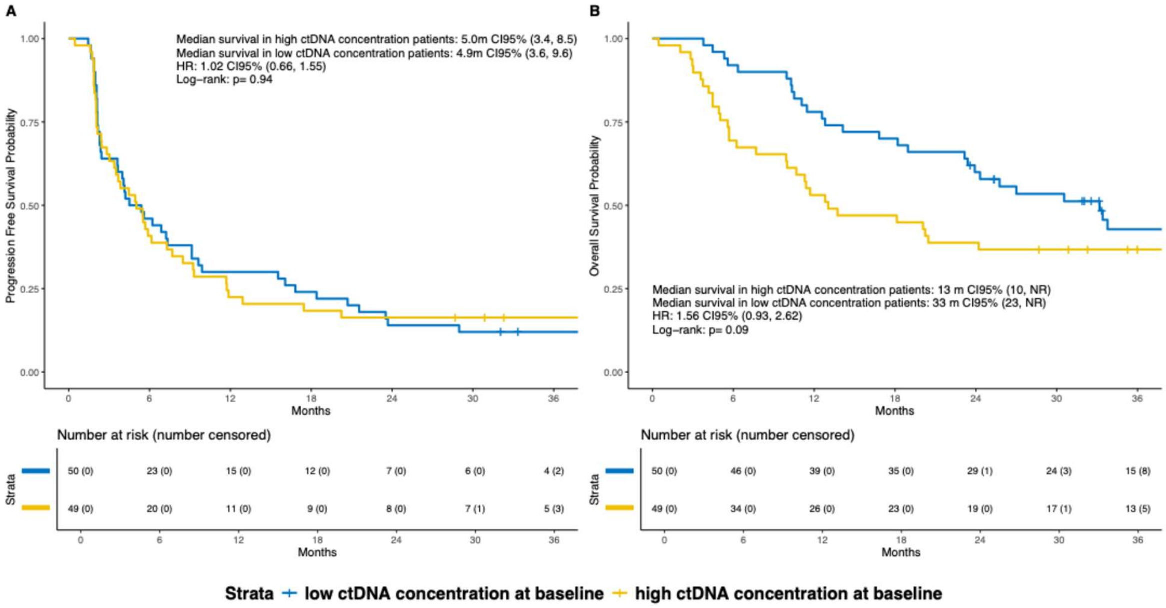

© 2025 Taïeb J et al. *JAMA Oncol*.

eFigure 3: PFS (A) and OS (B) according to ctDNA response. The two groups good and bad ctDNA responders were separated based on a median threshold of 86% ctDNA decrease.

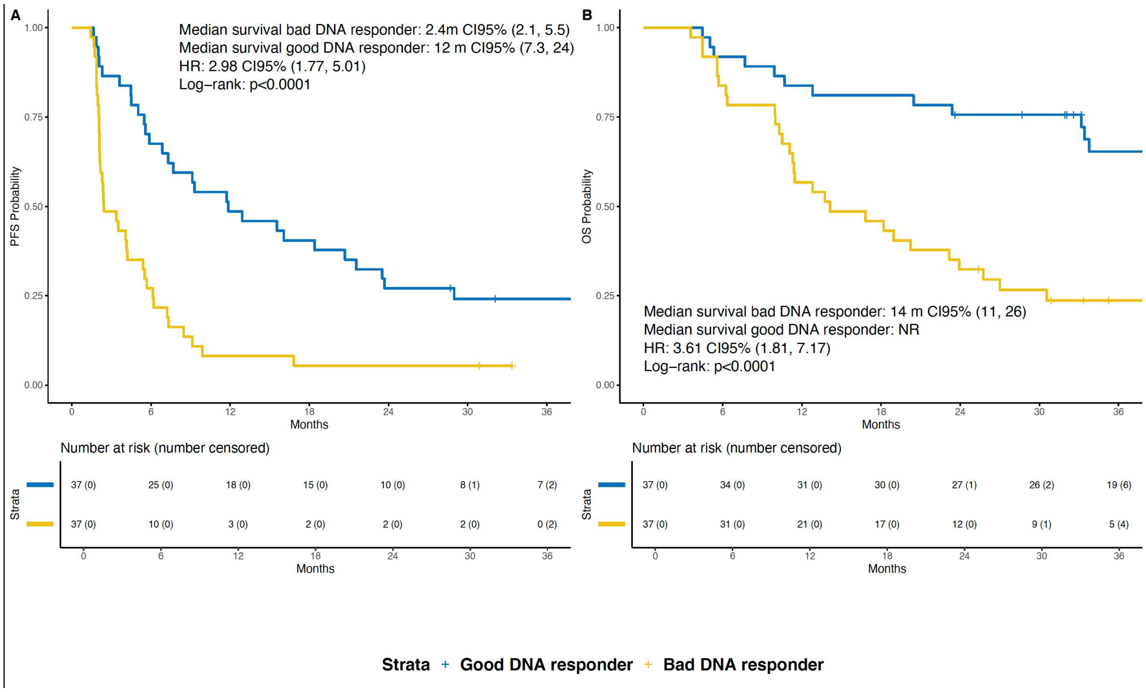

© 2025 Taïeb J et al. *JAMA Oncol*.

eFigure 4: DNA concentrations in ng/mL at baseline (V1) and after one month of treatment (V2) according to treatment arm and treatment response. Each patient is represented by a dot. Patients achieving disease control (CR+PR+SD) are represented in blue while those with achieving progressive disease (PD) as best response are represented in red. The numbers in brackets indicate the number of corresponding patients. Lines are in blue for good ctDNA responders and in yellow for bad ctDNA responders, the two groups were separated based on a median threshold of 86% ctDNA decrease.

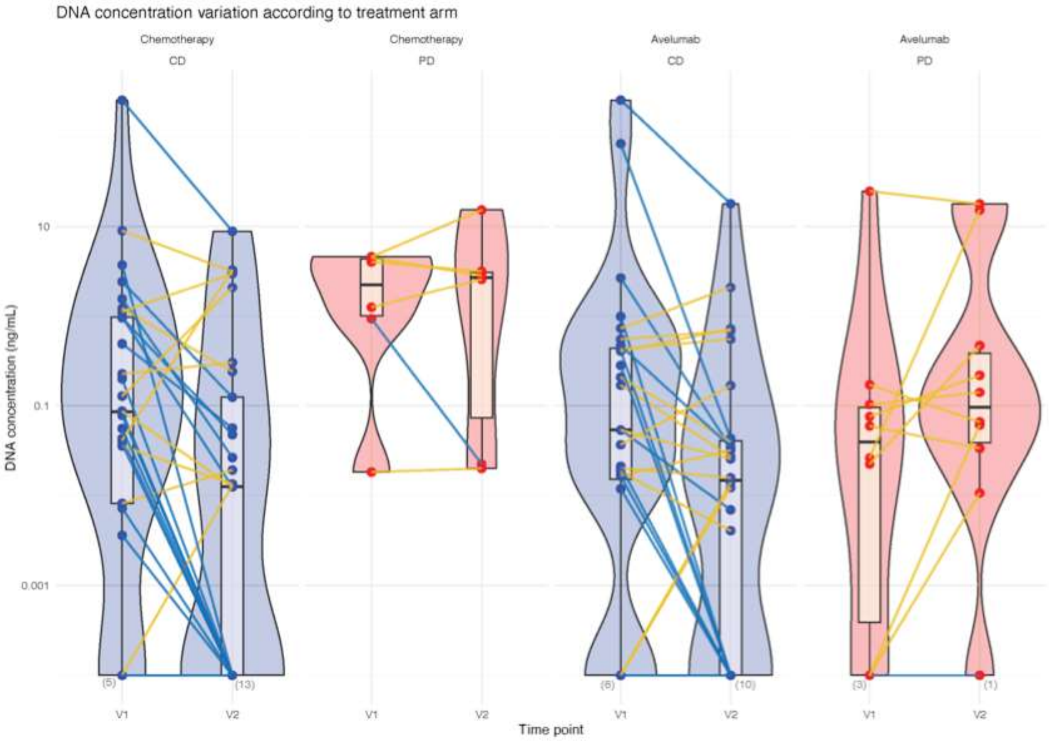

© 2025 Taïeb J et al. *JAMA Oncol*.

eFigure 5: Early ΔctDNA variations between V1 and V2, disease control according to RECIST and overall survival (D). Kaplan- Meyer curves of OS representing patients with assessable V1 and V2 ctDNA in the overall population (n= 74), stratified by the presence or absence of disease control, according to RECIST criteria. The yellow curve represents patients with a good ctDNA response and disease control (CR, PR and SD). The green curve represents patients with good ctDNA response and PD. The light blue curve represents patients with bad ctDNA response and disease control. The red curve represents patients with bad ctDNA response and PD. Two-sided Log-rank p = 0.00015.

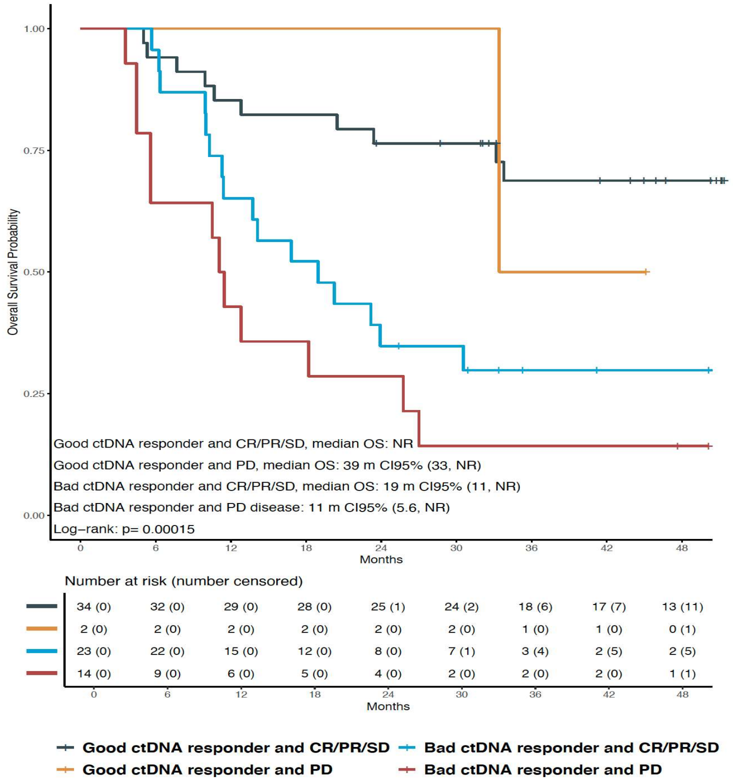

© 2025 Taïeb J et al. *JAMA Oncol*.

eTable 1. Relation of ctDNA response to standard prognostic variables for PFS in MSI mCRC patients treated with immunotherapy.

**Characteristic**	**Good DNA responder**, N = 37[1]	**Bad DNA responder**, N = 37[1]	**p-value**[2]
Liver metastases	15 (41%)	14 (38%)	0.8
Number of metastic sites			0.7
≤2	12 (35%)	10 (30%)	
>2	22 (65%)	23 (70%)	
Unknown	3	4	
Log transform CEA	0.87 (0.40, 1.30)	1.20 (0.74, 1.76)	0.079
Unknown	2	1	
Neutrophiles Lymphocytes Ratio (NLR)	2.28 (1.72, 3.23)	3.43 (2.36, 5.35)	0.014
Treatment arm			0.2
Chemotherapy	21 (57%)	15 (41%)	
Avelumab	16 (43%)	22 (59%)	

[1]n (%); Median (IQR)

[2]Pearson's Chi-squared test; Wilcoxon rank sum test; Wilcoxon rank sum exact test

© 2025 Taïeb J et al. *JAMA Oncol*.

eTable 2: Univariable analysis of PFS based on key clinical and molecular variables**.**

	All				Chemotherapy				Avelumab			
**Variable**	**N**	**HR[1]**	**95% CI[1]**	**P-value**	**N**	**HR[1]**	**95% CI[1]**	**P-value**	**N**	**HR[1]**	**95% CI[1]**	**P-value**
Sex	99				46				53			
Male		—	—			—	—			—	—	
Female		1.06	0.69, 1.63	0.776		0.89	0.49, 1.63	0.708		1.26	0.68, 2.35	0.465
Age	99	1.00	0.99, 1.02	0.619	46	1.01	0.98, 1.04	0.478	53	1.00	0.98, 1.02	0.825
Performance status	99				46				53			
0		—	—			—	—			—	—	
1 or 2		1.14	0.74, 1.75	0.544		1.42	0.78, 2.58	0.250		0.99	0.53, 1.84	0.979
Liver metastases	99				46				53			
no		—	—			—	—			—	—	
yes		1.49	0.97, 2.30	**0.067**		1.88	1.03, 3.43	**0.039**		1.18	0.63, 2.21	0.606
Number of metastatic sites	91				41				50			
≤2		—	—			—	—			—	—	
>2		1.47	0.91, 2.36	0.115		1.48	0.76, 2.92	0.251		1.41	0.72, 2.76	0.315
Log transform CEA	94	1.60	1.25, 2.04	**<0.001**	45	1.91	1.38, 2.65	**<0.001**	49	1.51	1.04, 2.19	**0.031**
Neutrophiles Lymphocytes Ratio (NLR)	99	1.07	1.01, 1.13	**0.028**	46	1.09	1.00, 1.18	**0.046**	53	1.05	0.97, 1.14	0.245
Albumin at inclusion	98	0.97	0.92, 1.01	0.129	45	0.95	0.89, 1.02	0.170	53	0.97	0.91, 1.04	0.410
LDH at inclusion	92	1.00	1.00, 1.00	0.619	42	1.00	1.00, 1.00	0.246	50	1.00	1.00, 1.00	0.847
GGT at inclusion	98	1.00	1.00, 1.00	0.262	45	1.00	1.00, 1.00	0.187	53	1.00	1.00, 1.00	0.381
BRAF mutation	99				46				53			
no		—	—			—	—			—	—	
yes		1.42	0.92, 2.19	0.112		1.48	0.78, 2.80	0.228		1.46	0.79, 2.71	0.229
ctDNA status before treatment	99				46				53			
negative		—	—			—	—			—	—	
positive		1.32	0.73, 2.38	0.364		1.35	0.53, 3.47	0.527		1.09	0.50, 2.36	0.835
ctDNA concentration before treatment	99				46				53			
low		—	—			—	—			—	—	
high		1.02	0.66, 1.55	0.945		1.39	0.76, 2.52	0.281		0.73	0.39, 1.37	0.330
Δ ctDNA	74				36				38			
Good ctDNA responders		—	—			—	—			—	—	
Bad ctDNA responders		2.98	1.77, 5.01	**<0.001**		2.09	1.03, 4.21	**0.040**		4.22	1.77, 10.1	**0.001**

[1]HR = Hazard Ratio, CI = Confidence Interval

© 2025 Taïeb J et al. *JAMA Oncol*.
