# Supplementary material for: Early ctDNA and Survival in Metastatic Colorectal Cancer Treated With Immune Checkpoint Inhibitors: A Secondary Analysis of the SAMCO-PRODIGE 54 Randomized Clinical Trial
Source: JAMA Oncol. 2025 Jun 18;11(8):874–82. doi: 10.1001/jamaoncol.2025.1646 (PMC12177728; doi:10.1001/jamaoncol.2025.1646)
Supplement: Supplement 3. — Data Sharing Statement [file jamaoncol-e251646-s003.pdf]

Data Sharing Statement

Taïeb. Early ctDNA and Survival in Metastatic Colorectal Cancer Treated With Immune Checkpoint Inhibitors. *JAMA Oncol*. Published June 18, 2025.
doi:10.1001/jamaoncol.2025.1646

Data
Data available: Yes
Data types: Deidentified participant data
How to access data: Individual data can be request to Pierre Laurent-Puig pierre.laurent-puig@u-paris.fr
When available: With publication

Supporting Documents
Document types: Statistical/analytic code
How to access documents: Individual data can be request to Pierre Laurent-Puig pierre.laurent-puig@u-paris.fr
When available: With publication

Additional Information
Who can access the data: researchers whose proposed use of the data has been approved
Types of analyses: The data will be made available for any purpose, including but not limited to the replication of results, exploratory analyses, and meta-analyses.
Mechanisms of data availability: After investigator and sponsor approval
